# Design, synthesis, and bioevaluation of pyrazole-containing tubulin polymerisation inhibitors based on conformational constraint strategy

**DOI:** 10.1080/14756366.2025.2545004

**Published:** 2025-09-23

**Authors:** Zhongqiao Sun, Jiahao Wang, Fengwei Li, Liancheng Huang, Shide Zheng, Qi Guan, Zhaohua Wang, Weige Zhang

**Affiliations:** ^a^ School of Pharmaceutical Engineering, Shenyang Pharmaceutical University, Shenyang, China; ^b^ Department of Otorhinolaryngology & Head and Neck Surgery, Dalian Municipal Friendship Hospital, Dalian, China

**Keywords:** Tubulin polymerisation inhibitor, conformational constraint strategy, pyrazole derivative, anti-tumor activity, anti-migration

## Abstract

Based on conformational preference of SMART analogues and conformational constraint strategy, two series of new tubulin polymerisation inhibitors (**4a **−** 4k** and **5a **−** 5h**/**6a **−** 6h**) were designed *via* hydrogen bonding, steric effect (for **4a **−** 4k**) and ring-closing approach by fused five- and seven-membered ring (for **5a **−** 5h**/**6a **−** 6h**) which was first adopted in the design of new SMART analogues. Among these compounds, **4k** and **5a** showed potent activities with IC_50_ values of 15 nM and 6 nM against PC-3 cell line. Mechanism studies indicated that **4k** and **5a** could inhibit tubulin polymerisation, arrest cell cycle at G_2_/M phase, induce cell apoptosis, and inhibit cell migration and colony formation. Molecular docking suggested that compounds **4k** and **5a** could bind into the colchicine binding site at the pose similar to DAMA-colchicine. Western blot assays revealed that **4k** and **5a** regulated the expression of cell cycle and apoptosis-related proteins. Prediction of physicochemical properties indicated good drug-likeness of **4k** and **5a**.

## Graphical abstract

**Figure:**
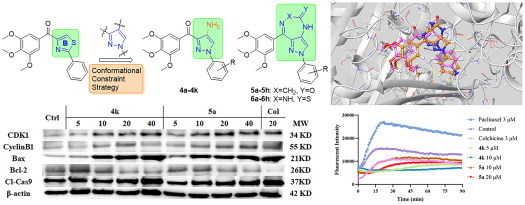

## Introduction

**Figure 1.**
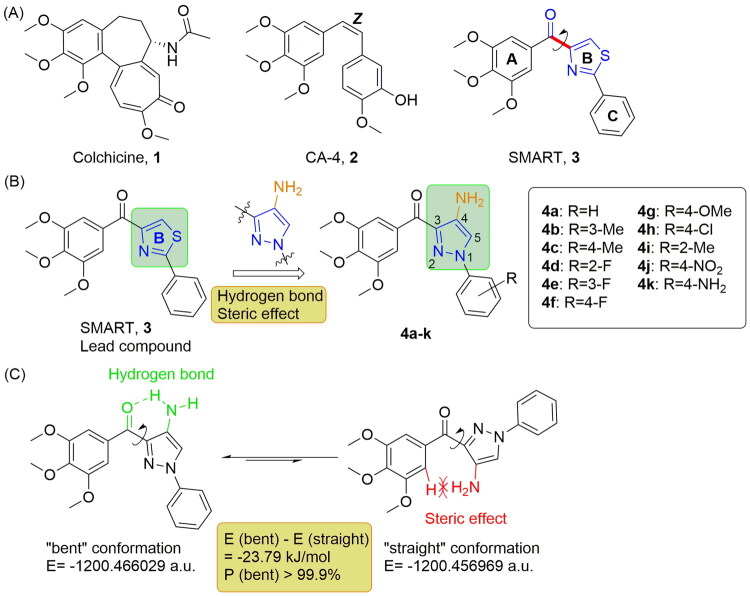
(A) Structures of three colchicine binding site inhibitors. (B) Structures and design of compound **4a**−**k**. (C) Energy and proportion of two conformations of **4a**. Hydrogen bond and steric effect were shown. The proportion of bent conformation can be calculated by these formulas: *E*(bent) − *E*(straight) = − *RT*ln*K*, *P*(bent) = [*n* (bent)]/[*n*(straight) + *n*(bent)] = *K*/(*K* + 1), 1 a.u. = 2625.5 kJ/mol.

Microtubules are long hollow polar filaments assembled from α- and β-tubulin dimer subunits^1–3^, playing key roles in various cellular functions, including cell motility, intracellular transport, cytokinesis, and cellular morphogenesis^4^^,^^5^. Because of these vital functions, microtubule becomes one of the most significant targets for anti-tumor drugs^6^. Microtubules targeted agents can be divided into two types: microtubule stabilisation agents (e.g. paclitaxel and laulimalide site agents) and microtubule destabilisation agents (e.g. colchicine and vinblastine site agents)^7^. Colchicine (**1**) and its analogues such as combretastatin A-4 (CA-4, **2**), known as colchicine binding site inhibitors (CBSIs), have attracted much attention due to their high activities against cancer cell lines, simple structures, and immunity to multidrug resistance mediated by P-glycoprotein (P-gp)^8^^,^^9^ (Figure 1(A)).

4-Substituted methoxybenzoyl-aryl-thiazole (SMART, **3**, Figure 1(A)), a representative CBSI reported by Li and co-workers, exhibits potent antiproliferative effects on a range of cancer cell lines with reduced neurotoxicity compared to vincristine^10^^,^^11^. With SMART as lead compound, numerous compounds involving the replacement of the thiazole (B-ring) of SMART with other aromatic heterocyclic rings have been designed and synthesised. An earlier investigation by our research group underscored the critical role of the conformational preference caused by B-rings on the bioactivity of SMART analogues^12^. The bent-conformer was the dominant conformation with higher activities, while the rotation of C-C bond between carbonyl group and B-ring (red bond in **3**, Figure 1(A)) to straight-conformer often led to a decrease in antiproliferative effects. Conformational constraint strategy is an efficient method to stabilise the active conformation of molecule^13^^,^^14^, and also useful in the design of novel SMART analogues. Introduction of intramolecular hydrogen bond (usually between substituent group on B-ring and carbonyl linkage) or steric effect could increase the percentage of “bent” conformation. Similarly, ring-closing approach involved B-ring could directly keep the “bent” conformation of the molecule, which might enhance the activity.

Pyrazole ring appears in multiple drugs that cover various pharmacological properties including anti-inflammatory, anti-tumor, antidepressant, etc^15–17^. Hence, we firstly designed (4-amino-1-aryl-1*H*-pyrazol-3-yl)(3,4,5-trimethoxyphenyl) methanone (**4a**−**k**) as a new series of SMART analogues. Pyrazole moiety was introduced into **4a**−**k** as B-ring to replace the thiazole moiety of SMART, and an amino group was introduced on the C4-position of pyrazole moiety to increase the proportion of “bent” conformation by hydrogen bond and steric effect (Figure 1(B,C)). Molecular energy calculation (DFT computation) by Gaussian 09 software showed that compound **4a** had a considerable proportion (>99.9%) of “bent” conformation (Figure 1(C)), which might exhibit potent activity.

**Figure 2.**
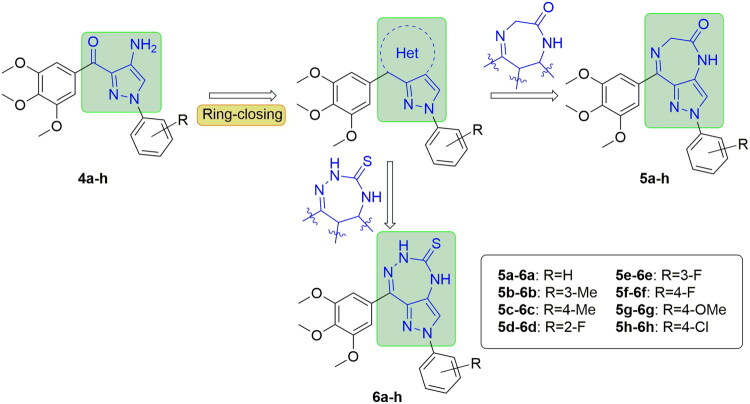
Structures and design of target compounds **5a**−**h** and **6a**−**h**.

Ring-closing is another common approach of conformational constraint strategy in medicinal chemistry^18^^,^^19^. In our previous works, some CBSIs that contain fused five-/five-membered or five-/six-membered ring scaffold were designed and synthesised with the application of the approach^19–21^. Diazepine is a widely used template which presents in several sedative-hypnotic drugs including diazepam, alprazolam, and olanzapine^22–24^. In recent years, some compounds with diazepine ring were discovered to exhibit bioactivities other than sedation, such as anti-asthma and anticancer activities^25^^,^^26^. Thus, the diazepine moiety was introduced, and a new series of CBSIs containing 2,6-dihydropyrazolo[4,3-*e*][1,4]diazepin-5(4*H*)-one scaffold (**5a**−**h**) was designed. Triazepine also presents in various compounds which demonstrate varied pharmacological properties including antimicrobial, immunosuppressive, and anticancer activities^27–29^. Hence, more CBSIs with a novel 2,6-dihydropyrazolo[4,3-*e*][1,2,4]triazepine-5(4*H*)-thione scaffold (**6a**−**h**) were designed. The two scaffolds replaced the carbonyl linkage and B-ring of **4a**−**h** through ring-closing approach to restrict the active conformation (Figure 2).

Herein, two series of compounds (**4a**−**k** and **5a**−**h**/**6a**−**h**) were designed as novel CBSIs through conformational constraint strategy of SMART analogues. This work provided a new perspective for further design of new tubulin polymerisation inhibitors.

## Results and discussion

### Chemistry

**Scheme 1.**
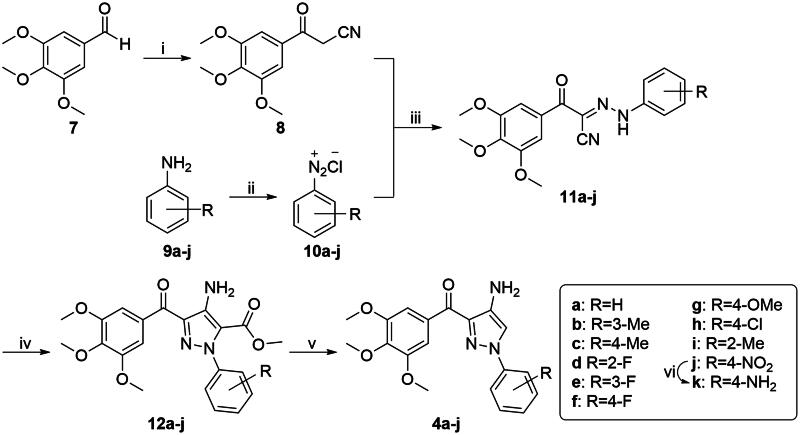
Reagents and conditions: (i) KOH, CuCl_2_·2H_2_O, CH_3_CN, DMA, O_2_ atmosphere, 25 °C, 36 h; (ii) Conc. HCl, NaNO_2_ aq., H_2_O, 0 − 5 °C, 0.5 h; (iii) AcONa, EtOH/H_2_O, 0 − 5 °C, 2 h; (iv) BrCH_2_COOCH_3_, K_2_CO_3_, DMF, 130 °C, 2 h; (v) a) NaOH, H_2_O, 80 °C, 3 h; b) Conc. HCl, *n*-PrOH, 80 °C, 30 min; and (vi) SnCl_2_·2H_2_O, EtOH, reflux, 3 h.

The synthetic route of target compounds **4a**−**k** was shown in Scheme 1. The 3,4,5-trimethoxybenzaldehyde (**7**) reacted with acetonitrile in the presence of copper chloride and potassium hydroxide in *N*,*N*-dimethylacetamide under oxygen atmosphere at room temperature to afford 3-oxo-3–(3,4,5-trimethoxyphenyl)propanenitrile (**8**)^30^. Substituted diazonium salts (**10a**−**j**) were synthesised from the corresponding phenylamines (**9a**−**j**). Subsequently, compound **8** was coupled with different diazonium salts (**10a**−**j**) to give corresponding hydrazone derivatives (**11a**−**j**). 4-Amino-1-aryl-3–(3,4,5-trimethoxybenzoyl)-1*H*-pyrazole-5-carboxylates (**12a**−**j**) were formed *via* cyclisation of **11a**−**j** with methyl bromoacetate based on a previous reported work^31^. Then, **12a**−**j** were subsequently hydrolysed and decarboxylated to obtain desired compounds **4a**−**j**. Compound **4k** was synthesised by nitro reduction of **4j**.

**Scheme 2.**
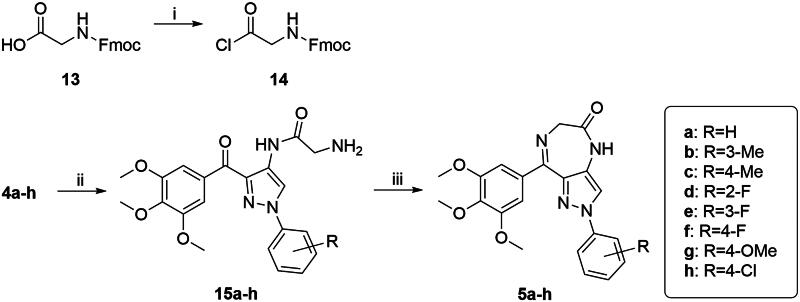
Reagents and conditions: (i) SOCl_2_, CH_2_Cl_2_, reflux, 2 h; (ii) a) **14**, CH_2_Cl_2_, rt, 3 h; b) piperidine, rt, 0.5 h; and (iii) AcOH, EtOH, reflux, 3 h.

The synthetic route of target compounds **5a**−**h** was shown in Scheme 2. Compounds **5a−h** could be synthesised from **4a**−**4h**. Acyl chloride **14** was obtained by the reaction of Fmoc-glycine (**13**) and thionyl chloride in CH_2_Cl_2_ at room temperature. Compounds **15a**−**h** were afforded through acylation of amino group on the C4-position of pyrazole of **4a**−**h** with **14** and subsequently the deprotection of Fmoc group in CH_2_Cl_2_ at room temperature. The desired compounds **5a**−**h** were formed by cyclodehydration of **15a**−**h** in ethanol containing 5% of acetic acid under reflux.

**Scheme 3.**
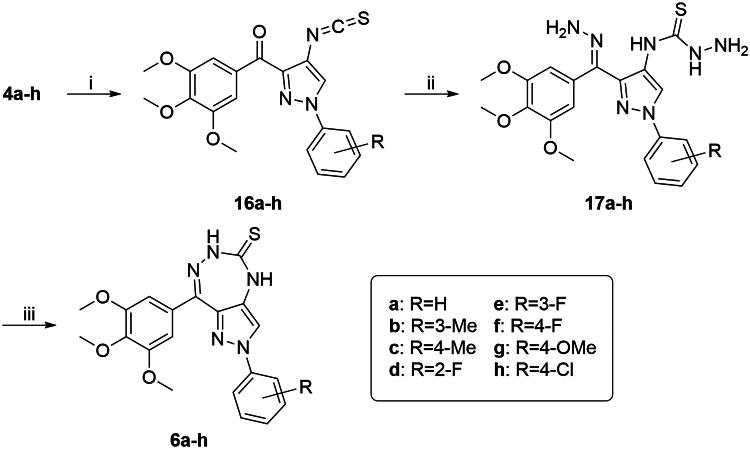
Reagents and conditions: (i) CSCl_2_, CH_2_Cl_2_/NaHCO_3_ aq., rt, 1 h; (ii) N_2_H_4_·H_2_O (80%), EtOH, reflux, 3 h; and (iii) TsOH·H_2_O, EtOH, reflux, 3 h.

The synthetic route of target compounds **6a**−**h** was shown in Scheme 3. Compounds **16a**−**h** containing isothiocyanato group were obtained by treatment of **4a**−**h** with thiophosgene in CH_2_Cl_2_/NaHCO_3_ aq. at room temperature^32^. Hydrazinecarbothioamide derivatives (**17a**−**h**) were synthesised using **16a**−**h** with excess hydrazine hydrate in ethanol under reflux. The desired compounds **6a**−**h** were obtained by intramolecular cyclisation of **17a**−**h** with TsOH·H_2_O as catalyst in ethanol at reflux^33^.

### In vitro antiproliferative activity

The *in vitro* antiproliferative activities of all target compounds **4a**−**k**, **5a**−**h**, and **6a**−**h** were assayed by MTT method using human gastric cancer cell line SGC-7901 and human breast cancer cell line MCF-7. Colchicine was used as the positive control. The result was shown in Table 1.

**Table 1.** IC_50_ of compounds **4a**−**k**, **5a**−**h,** and **6a**−**h** against MCF-7 and SGC-7901 cells.

Compounds	IC_50_ (μM) ± *SD*^a^	
	SGC-7901	MCF-7
**4a**	0.040 ± 0.003	0.034 ± 0.005
**4b**	0.095 ± 0.011	0.224 ± 0.060
**4c**	0.234 ± 0.033	3.90 ± 0.205
**4d**	0.086 ± 0.004	0.404 ± 0.036
**4e**	0.439 ± 0.037	0.350 ± 0.023
**4f**	0.092 ± 0.005	0.162 ± 0.010
**4g**	**0.022 ± 0.002**	0.068 ± 0.012
**4h**	0.318 ± 0.020	0.098 ± 0.007
**4i**	0.166 ± 0.004	2.20 ± 0.196
**4j**	0.040 ± 0.006	0.391 ± 0.083
**4k**	**0.017 ± 0.002**	**0.031 ± 0.005**
**5a**	**0.018 ± 0.003**	1.78 ± 0.617
**5b**	0.424 ± 0.076	0.665 ± 0.052
**5c**	0.117 ± 0.010	1.381 ± 0.433
**5d**	0.053 ± 0.008	1.08 ± 0.381
**5e**	0.949 ± 0.017	3.01 ± 0.534
**5f**	0.137 ± 0.025	1.86 ± 0.438
**5g**	0.841 ± 0.024	2.86 ± 0.435
**5h**	0.772 ± 0.004	0.857 ± 0.090
**6a**	2.74 ± 0.041	2.98 ± 0.462
**6b**	0.366 ± 0.065	0.749 ± 0.181
**6c**	0.151 ± 0.024	0.123 ± 0.020
**6d**	0.087 ± 0.008	0.398 ± 0.079
**6e**	5.12 ± 0.099	6.90 ± 0.815
**6f**	10.0 ± 0.078	8.42 ± 0.954
**6g**	2.16 ± 0.098	1.57 ± 0.243
**6h**	0.485 ± 0.030	0.662 ± 0.070
Colchicine^b^	0.031 ± 0.006	0.043 ± 0.016

^a^IC_50_: 50% inhibitive concentration after 72 h of drug exposure, as determined by MTT assay. Each experiment was carried out in triplicate.

^b^Colchicine was used as positive control.

The boldface means the high activity data which we want to highlight.

Most of the compounds in two series exhibited moderate to high antiproliferative activities. Among **4a**−**k**, compounds **4a**, **4 g**, and **4k** showed higher activities, which suggested that no substitution or substitution of polar group at the *para*-position of C-ring could increase the activity. This view could also be supported by potent activities of **4f** and **4j**. Compound **4k** showed the highest activity with the IC_50_ values of 0.017 ± 0.002 μM against SGC-7901 cell line and 0.031 ± 0.005 μM against MCF-7 cell line.

Compounds **5a**−**h** also showed moderate to high activities against cancer cell lines. The higher activities of **5a**, **5c**, **5d**, and **5f** than other compounds in **5a**−**h** suggested that no substitution or substitution of small group at the *ortho*-/*para*-position of C-ring could increase the activity of compounds in this series. Meanwhile, *meta*-substitution or large substituent (**5b**, **5e**, and **5g**) might decrease the activity. Compound **5a** showed the highest activity among series **5a**−**h** with the IC_50_ values of 0.018 ± 0.003 μM against SGC-7901 cells.

Among **6a**−**h**, compounds with *para*-methyl (**6c**) or *ortho*-fluoro (**6d**) substitution showed high activities (IC_50_ values of 0.151, 0.087 μM against SGC-7901 cell line and 0.123, 0.398 μM against MCF-7 cell line, respectively), while *meta*- or *para*-fluoro substitution (**6e** and **6f**) led to the decrease of activity.

In general, series **4a**−**k** exhibited similar potency as series **5a**−**h** against SGC-7901 cell line and better potency against MCF-7 cell line. Series **6a**−**h** showed the lowest activities among the two series. Compound **4k** showed the highest activities against SGC-7901 and MCF-7 cell lines.

Additional cell lines including human colon cancer cell line HCT-116, human non-small cell lung cancer cell line A549, and human prostate cancer cell line PC-3 were brought to test the antiproliferative activities of potent compounds **4k** and **5a**. As shown in Table 2, compound **4k** exhibited high activities against three additional cell lines with the IC_50_ values of 0.010 μM against HCT-116 cell line. Meanwhile, compound **5a** exhibited high activities against A549 and PC-3 cell lines (with IC_50_ values of 0.063 and 0.006 μM) but less potency against HCT-116 (0.163 μM) cell line. Despite the diversity of activities against different cell lines, compound **5a** showed the highest activities against PC-3 cells with the IC_50_ values of 0.006 μM.

**Table 2.** IC_50_ of compounds **4k** and **5a** against HCT-116, A549 and PC-3 cells.

Compounds	IC_50_ (μM) ± *SD*^a^		
	HCT-116	A549	PC-3
**4k**	0.010 ± 0.001	0.040 ± 0.012	0.015 ± 0.004
**5a**	0.163 ± 0.038	0.063 ± 0.017	0.006 ± 0.002
Colchicine^b^	0.039 ± 0.008	0.043 ± 0.015	0.015 ± 0.003

^a^IC_50_: 50% inhibitive concentration after 72 h of drug exposure, as determined by MTT assay. Each experiment was carried out in triplicate.

^b^Colchicine was used as positive control.

The toxicity of compounds **4k** and **5a** was also tested by using human umbilical vein endothelial cells (HUVEC) and human liver cells (L02). The result in Table 3 showed that the SI-H (selectivity indexes of HUVEC, IC_50_ in normal cells HUVEC/IC_50_ in cancer cells PC-3) of **4k**, **5a**, and colchicine were 39.11, 147.7, and 13.95, respectively, which indicated lower toxicities of **4k** and **5a** than that of colchicine. Moreover, the SI-L (selectivity indexes of L02) of **5a** was 35.00, which might indicate lower toxicity of **5a** than **4k** and colchicine.

**Table 3.** IC_50_ of compounds **4k** and **5a** against HUVEC and L02 cells.

Compounds	IC_50_ (μM) ± *SD*^a^			
	HUVEC	SI-H^c^	L02	SI-L^d^
**4k**	0.570 ± 0.167	39.11	0.025 ± 0.008	1.713
**5a**	0.860 ± 0.229	147.7	0.204 ± 0.056	35.00
Colchicine^b^	0.210 ± 0.078	13.95	0.012 ± 0.003	0.797

^a^IC_50_: 50% inhibitive concentration after 72 h of drug exposure, as determined by MTT assay. Each experiment was carried out in triplicate.

^b^Colchicine was used as positive control.

^c^SI-H = IC_50_ (HUVEC)/IC_50_ (PC-3).

^d^SI-L = IC_50_ (L02)/IC_50_ (PC-3).

## Computational studies

### Molecular docking study

**Figure 3.**
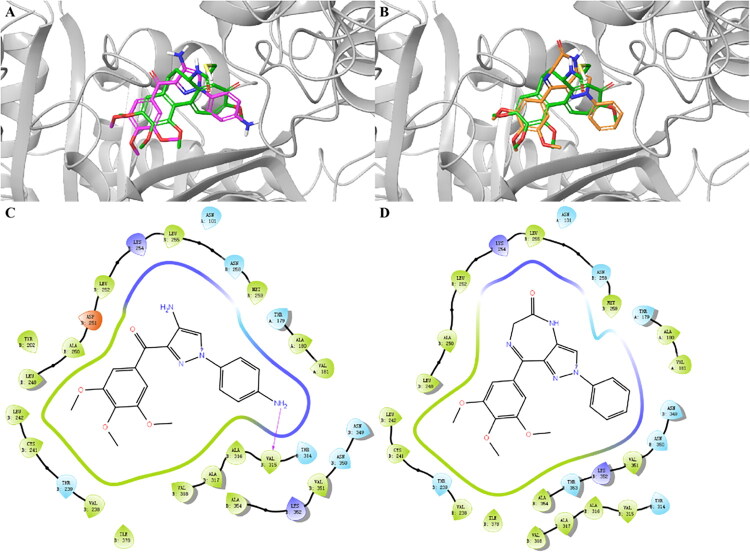
(A, B) Binding poses of **4k** (pink), **5a** (orange) compared with DAMA-colchicine (green) at the colchicine binding site of tubulin (PDB: 1SA0). (C) Ligand-acceptor interaction between **4k** and tubulin. Pink arrow represents the hydrogen bond. (D) Ligand-acceptor interaction between **5a** and tubulin.

The most potent compounds **4k** and **5a** were chosen to explain the potency through molecular interactions. The docking results of compounds with DAMA-colchicine-tubulin complex (PDB: 1SA0) were shown in Figure 3. Both **4k** and **5a** could bind with α and β-tubulin at colchicine binding site which is similar to the pose of DAMA-colchicine. The A-rings and C-rings of **4k** and **5a** occupied the two hydrophobic grooves of the binding site (Figure 3(C,D)). Hydrophobic interactions between compounds **4k**/**5a** and several important residues (such as β-Cys241, β-Ala250, β-Val315, β-Lys352, α-Val181) could also be observed. Moreover, the binding site had enough space to accommodate the amino group on B-ring of **4k** and the diazepine ring of **5a**. Notably, a hydrogen bond which was formed by the amino group on C-ring of **4k** with β-Val315 was observed. These interactions might enhance the interaction between **4k**/**5a** and tubulin, thus increasing the antiproliferative activity of the compounds.

### Molecular dynamic simulation study

**Figure 4.**
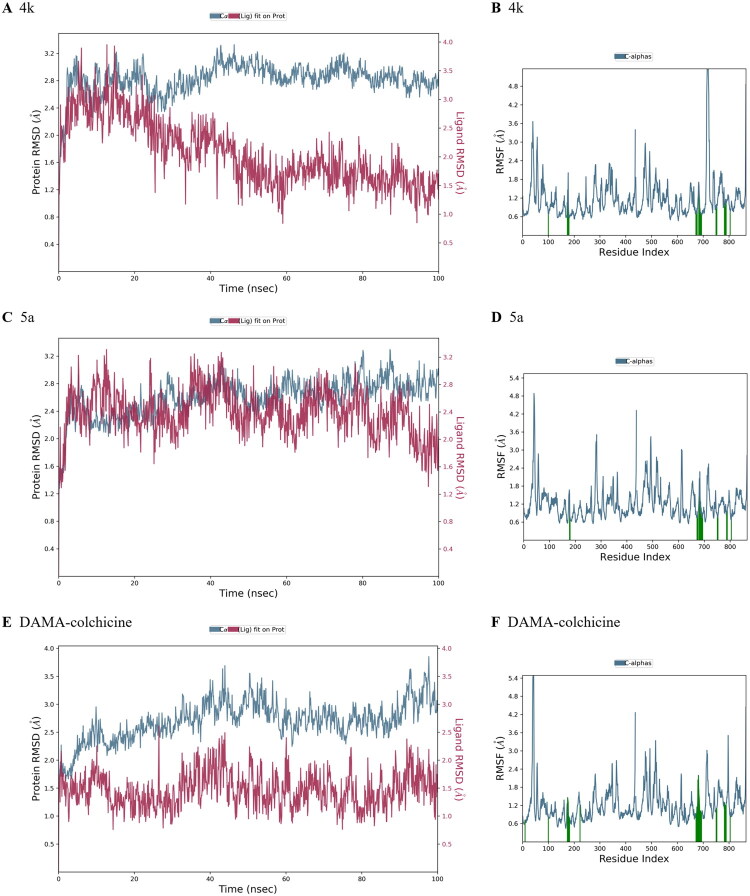
RMSD and RMSF analysis of three binding models. Green lines in RMSF analysis represented ligand contacts. (A) RMSD of tubulin and compound **4k** in binding model. (B) RMSF of tubulin atoms in binding model with compound **4k**. (C) RMSD of tubulin and compound **5a** in binding model. (D) RMSF of tubulin atoms in binding model with compound **5a**. (E) RMSD of tubulin and DAMA-colchicine in binding model. (F) RMSF of tubulin atoms in binding model with DAMA-colchicine.

To further investigate the binding mode, three 100 ns molecular dynamic simulations were conducted for tubulin (PDB: 1SA0) with docked poses of compounds **4k**, **5a**, and native ligand DAMA-colchicine. The root mean square deviations (RMSD) and root mean square fluctuations (RMSF) data were collected for analysis. As shown in Figure 4, the RMSD values of **4k** and **5a** were around 1.5 Å and 2.0 Å after 100 ns simulation, which demonstrated good stability of two binding model. The RMSF values of tubulin atoms with ligand **4k**, **5a**, and DAMA-colchicine exhibited similar fluctuations and ligand contacts (represented by green lines), which suggested similar binding mode between the three ligand (**4k**, **5a**, and DAMA-colchicine) and tubulin.

### Physicochemical properties

The physicochemical properties of compounds **4k**, **5a** and SMART (lead compound) were predicted *via* SwissADME website (http://www.swissadme.ch/index.php) to assess their drug-likeness. As shown in Table 4, the measured values of compounds **4k**, **5a** were in the range of drug-like properties, which suggested that compounds **4k**, **5a** exhibited good drug-likeness without violation of Lipinski’s Rule of Five^34^. In addition, the predicted LogS value of **4k** (−4.73) and **5a** (−4.11) were higher than that of SMART (−5.71), indicating better water solubilities of **4k** and **5a** compared to SMART.

**Table 4.** Prediction of physicochemical properties of SMART, **4k**, and **5a**.^a^

Compounds	MW	cLogP	HBD	HBA	TPSA	RB	LogS
Standard	<500	<5	<5	<10	<140	<10	–
**4k**	368.39	1.96	2	5	114.62	6	−4.73
**5a**	392.41	1.46	1	6	86.97	5	−4.11
SMART	355.41	3.70	0	5	85.89	6	−5.71

^a^MW: molecular weight (g/mol); cLogP: consensus LogP (average of iLOGP, XLOGP3, WLOGP, MLOGP, and Silicos-IT Log P); HBD: hydrogen bond donor; HBA: hydrogen bond acceptor; TPSA: topological polar surface area (Å^2^); RB: rotatable bond; LogS: Ali topological method Log S.

### In silico toxicity study

The toxicity data of compounds **4k** and **5a** were predicted by ADMETlab 3.0 website (https://admetlab3.scbdd.com/). As shown in Table 5, compound **4k** exibited less toxicity among several toxicity related properties including hERG-10, SkinSen, EI, and H-HT than SMART, while **5a** exhibited less toxicity on hERG-10, DILI, Ames, EI, and Resp. The result suggested the toxicity decrease of compounds **4k** and **5a** in some certain aspects compared with SMART.

**Table 5.** Prediction of toxicity data of SMART, **4k**, and **5a**.^a^

Compd	hERG-10	DILI	Ames	SkinSen	EI	Resp	H-HT
**4k**	0.514	0.956	0.945	0.182	0.401	0.904	0.571
**5a**	0.510	0.799	0.440	0.638	0.088	0.472	0.822
SMART	0.572	0.949	0.506	0.427	0.783	0.771	0.640

^a^hERG-10: hERG blockers (10 μm); DILI: drug induced liver injury; Ames: AMES mutagenicity; SkinSen: skin sensitisation; EI: eye irritation; Resp: Respiratory; H-HT: human hepatotoxicity. The output value is the probability of being positive, within the range of 0 to 1.

### Inhibition of tubulin polymerisation

**Figure 5.**
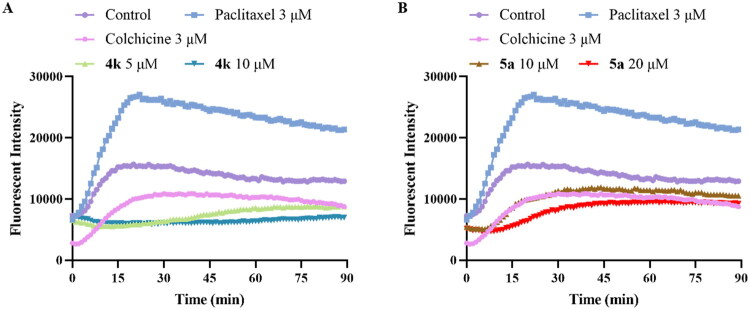
Effects of **4k** (**A**) and **5a** (**B**) on tubulin polymerisation. Tubulin had been incubated with **4k** (5 μM and 10 μM), **5a** (10 μM and 20 μM), paclitaxel (3 μM), colchicine (3 μM) or vehicle DMSO.

To investigate the effect of compounds **4k** and **5a** on microtubule system, their tubulin polymerisation inhibition activities were evaluated using *in vitro* tubulin polymerisation assay. Colchicine was used as positive control for inhibition of tubulin polymerisation, while paclitaxel was used as negative control. As shown in Figure 5, when tubulin was treated with **4k** (5 μM and 10 μM), **5a** (10 μM and 20 μM) or colchicine (3 μM), the increasing tendency of the fluorescence intensity slowed down, which indicated the inhibition of tubulin polymerisation. Compound **4k** at 5 μM and 10 μM exhibited higher potency than colchicine at the concentration of 3 μM, while **5a** at 20 μM also demonstrated superior inhibitory potency compared to colchicine (3 μM). Besides, compounds **4k** and **5a** showed dose-dependent inhibition of tubulin polymerisation, as higher concentration showed stronger inhibitory activity. In contrast, paclitaxel enhanced microtubule polymerisation. These results suggested that **4k** and **5a** act as potent tubulin polymerisation inhibitors, likely targeting the colchicine-binding site.

### Immunofluorescence study

**Figure 6.**
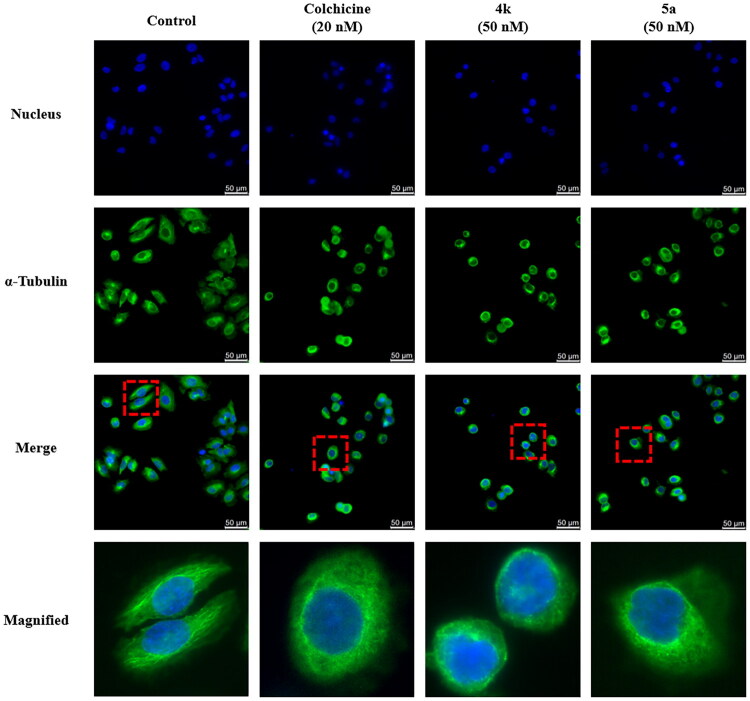
**4k**, **5a**, and colchicine influenced microtubule structure in PC-3 cells. Cells were treated with colchicine (20 nM), **4k** (50 nM), **5a** (50 nM), or vehicle for 24 h, fixed, and stained with anti-α-tubulin-FITC specific antibodies followed by DAPI. α-Tubulin was shown in green, while DNA was shown in blue.

To evaluate the effects of **4k** and **5a** on intracellular microtubule, immunofluorescence assay was carried out to observe the changes of microtubule network in cell. PC-3 cells were chosen in this test, and colchicine was used as positive control. As shown in Figure 6, in comparison with control group, most of the cells treated with colchicine (20 nM), **4k** (50 nM), or **5a** (50 nM) showed shape changes compared to the control group, and the microtubule network was destroyed. The results indicated that compounds **4k** and **5a** inhibited tubulin polymerisation in cancer cells similarly to the colchicine.

### Cell cycle study

**Figure 7.**
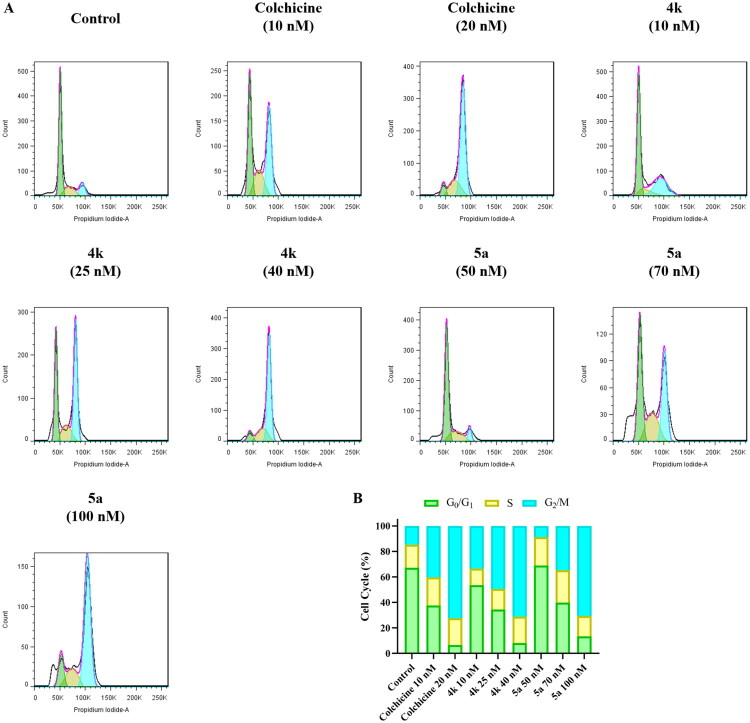
(A) Cell cycle distribution of PC-3 cells treated with colchicine (10 nM and 20 nM), **4k** (10 nM, 25 nM, and 40 nM), **5a** (50 nM, 70 nM and 100 nM), or vehicle for 24 h. Green: G_0_/G_1_ phase. Yellow: S phase. Cyan: G_2_/M phase. (B) Column stacking diagram of cell cycle distribution.

The G_2_/M phase of cell cycle involves mass physiological activities of microtubules, and tubulin polymerisation inhibitors could arrest cell cycle in G_2_/M phase by inhibiting microtubule polymerisation. To investigate the effect of **4k** and **5a** on cell cycle, flow cytometry was used to analyse the cell cycle of PC-3 cells which were treated with colchicine (10 nM, 20 nM), **4k** (10 nM, 25 nM, 40 nM), **5a** (50 nM, 70 nM, 100 nM), or vehicle. As shown in Figure 7, after treating with different concentration of **4k** and **5a**, the proportion of cells in G_2_/M phase increased from 15% to 33–71% (for **4k** at 10–40 nM), and from 15% to 35% or 71% (for **5a** at 70 or 100 nM), respectively. The result showed that compounds **4k** and **5a** could arrest PC-3 cells in G_2_/M phase which was similar to colchicine.

**Figure 8.**
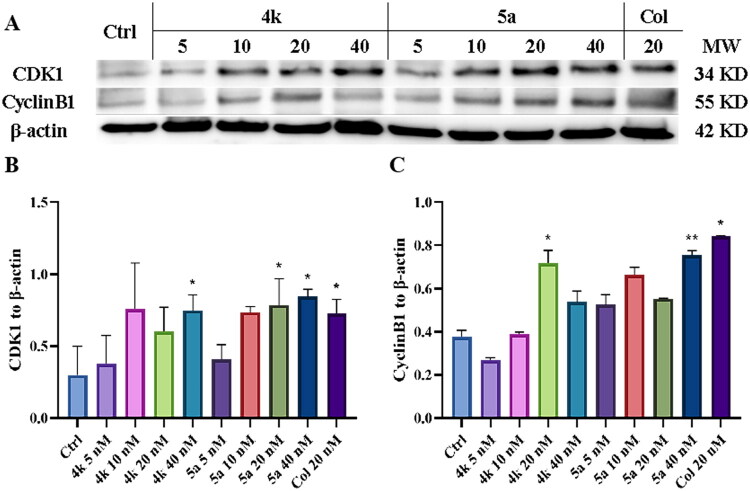
(A) Western blot assay for cell cycle-related proteins CDK1 and Cyclin B1. (B, C) Quantitative analysis of CDK1 and Cyclin B1. Ctrl: Control. Results were expressed as mean ± *SD*, *p < 0.05, **p < 0.01.

Additionally, western blot assay was employed to measure the expression level of cell cycle-related proteins cyclin-dependent kinase 1 (CDK1) and Cyclin B1. As shown in Figure 8, compounds **4k** and **5a** could increase the level of CDK1 at the concentrations of 40 and 20 nM, as well as the level of Cyclin B1 at the concentrations of 20 and 40 nM, which are about one to three times the value of IC_50_. These results suggested that **4k** and **5a** disrupt microtubule dynamics, leading to G_2_/M phase arrest and dysregulation of cell cycle-related proteins.

### Cell apoptosis analysis

Tubulin polymerisation inhibition is proved to further induce apoptosis in cancer cells^35–38^. An MTT assay was made on PC-3 cell line to test the cell viability after treated with different concentrations of compounds **4k** and **5a** after 24 h which was the same period as immunofluorescence assay. The result showed decreases in cell viability after cells treated with **4k** (from 91.6% to 41.6% at the concentrations of 0.51 nM to 1111 nM) and **5a** (from 76.6% to 39.6% at the concentrations of 0.51 nM to 1111 nM), which suggested cell apoptosis caused by tubulin polymerisation inhibition.

**Figure 9.**
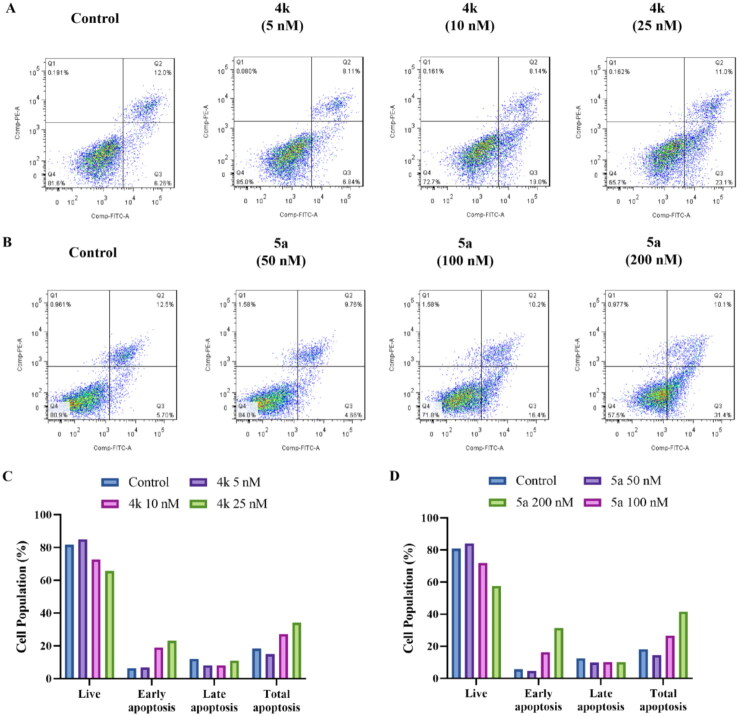
(A, B) Cell apoptosis distribution of PC-3 cells treated with **4k** (5 nM, 10 nM, and 25 nM), **5a** (50 nM, 100 nM, and 200 nM), or vehicle for 48 h. (C, D) The percentage of live and apoptosis cells in each concentration of **4k** or **5a**.

To investigate the effect of **4k** and **5a** on cell apoptosis, flow cytometry was used to analyse the cell apoptosis of PC-3 cells. Cells treated with colchicine (10 nM, 20 nM), **4k** (5 nM, 10 nM, 25 nM), **5a** (50 nM, 100 nM, 200nM) or vehicle were stained using Annexin V-FITC/PI cell apoptosis detection kit. Early apoptosis cells could only be stained by Annexin V-FITC, while late apoptosis cells could be stained by both Annexin V-FITC and PI. As shown in Figure 9, compound **4k** could increase the percentage of apoptosis cells from 18.3% to 27.1% (10 nM) and 34.1% (25 nM), and compound **5a** could also increase the percentage of apoptosis cells from 18.2% to 26.6% (100 nM) and 41.5% (200 nM).

**Figure 10.**
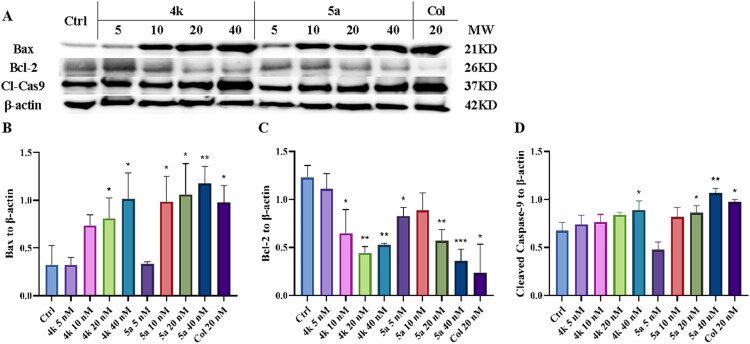
(A) Western blot assay for cell apoptosis-related proteins Bax, Bcl-2 and cleaved caspase-9. (B–D) Quantitative analysis of Bax, Bcl-2 and cleaved caspase-9. Ctrl: control. Results were expressed as mean ± *SD*, *p < 0.05, **p < 0.01, ***p < 0.001.

The expression of cell apoptosis-related proteins (Bax, Bcl-2, and cleaved caspase-9) after treatment of **4k** and **5a** was also measured by western blot assay. As shown in Figure 10, compounds **4k** and **5a** could increase the level of Bax, cleaved caspase-9 and decrease the level of Bcl-2 in a dose-dependent manner. The expression of Bax in PC-3 cells was significantly increased after treating with 20, 40 nM of **4k** or 10, 20, 40 nM of **5a**, while the expression of Bcl-2 was significantly decreased after treating with 10, 20, 40 nM of **4k** or 5, 20, 40 nM of **5a**. The results indicated that compounds **4k** and **5a** could significantly induce cell apoptosis by modulating apoptosis-related proteins.

## Cell migration assays

**Figure 11.**
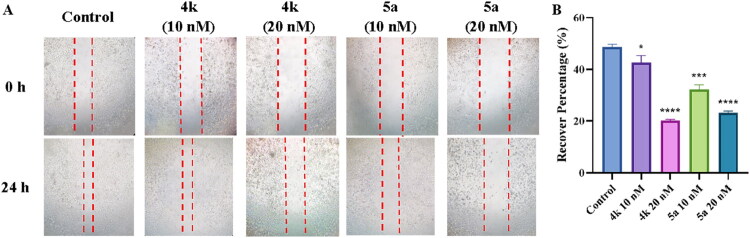
(A) Pictures showed the effects of compounds **4k** and **5a** on cell migration. (B) The recover percentage of scratches in each group after 24 h. Length was measured by Fiji/ImageJ software. Results were expressed as mean ± *SD*, *p < 0.05, ***p < 0.001, ****p < 0.0001.

To investigate the effect of **4k** and **5a** on cell migration, wound healing assay was used on PC-3 cells. As shown in Figure 11, when PC-3 cells were treated with 10 nM, 20 nM of **4k** or 10 nM, 20 nM of **5a**, it demonstrated a decrease in migration activity with the recover percentage of 42.7% (10 nM of **4k**), 20.2% (20 nM of **4k**), 32.3% (10 nM of **5a**) and 23.3% (20 nM of **5a**) compared to the control group (48.7%).

**Figure 12.**
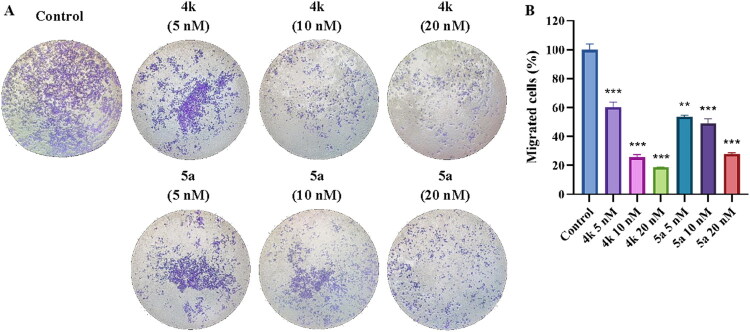
(A) Images of the transwell migration assay. Cells were treated with **4k** (5 nM, 10 nM, and 20 nM), **5a** (5 nM, 10 nM, and 20 nM) and vehicle for 24 h and were stained with crystal violet solution. (B) Results of relative migrated cells percentage compared to the untreated cells. Results were expressed as mean ± *SD*, **p < 0.01, ***p < 0.001.

Transwell migration assay was used on PC-3 cells to further investigate the effect of **4k** and **5a** on cell migration. As shown in Figure 12, the percentage of migrated cells had remarkable decreases after treating with **4k** (5, 10, and 20 nM) or **5a** (5, 10, and 20 nM) for 24h, exhibiting 60.4%, 25.7%, 18.7% (for **4k**) or 53.6%, 49.1%, 27.8% (for **5a**), respectively, which was presented as a dose-dependent manner. These results indicated the inhibitory effects of **4k** and **5a** on PC-3 cell migration.

### Colony formation assay

**Figure 13.**
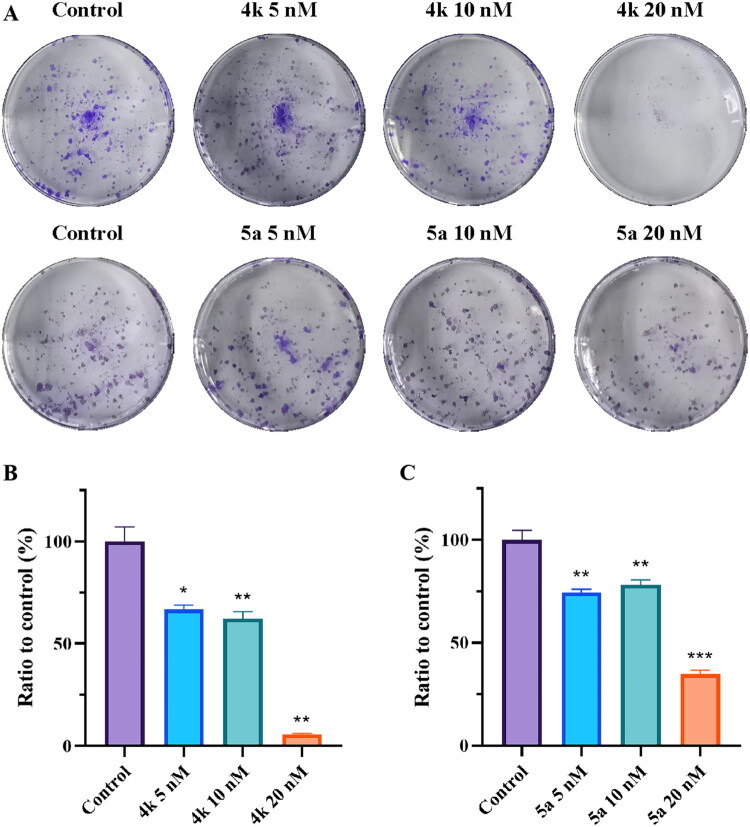
(A) Colony formation assay of PC-3 cells treated with different concentrations of compounds **4k** and **5a**. Cells were treated with **4k** (5 nM, 10 nM, and 20 nM), **5a** (5 nM, 10 nM, and 20 nM) and vehicle for 24 h and were continuously cultured for another one week in fresh medium containing 10% FBS. (B, C) Results of the ratio of each concentration to control. Results were expressed as mean ± *SD*, *p < 0.05, **p < 0.01, ***p < 0.001.

Colony formation assay was selected to assess the effect of **4k** and **5a** on clonogenic formation of PC-3 cells. As shown in Figure 13, compounds **4k** and **5a** could inhibit colony formation in a dose-dependent manner. Remarkably, the colony formation of PC-3 cells was nearly completely inhibited (5.7% compared to control) after treated with 20 nM of **4k**. Colony formation of PC-3 cells treated with 20 nM of **5a** was also inhibited to 34.9% compared to the control group. The results suggested that compounds **4k** and **5a** could inhibit colony formation of PC-3 cells, demonstrating the potent activities of **4k** and **5a** in disrupting colony formation of PC-3 cells.

## Conclusion

In this work, two new series of tubulin polymerisation inhibitors (**4a **−** 4k** and **5a **−** 5h**/**6a **−** 6h**) were designed based on the conformational constraint strategy of SMART analogues. Compounds **4a **−** 4k** were designed *via* hydrogen bonding and steric effect, and compounds **5a **−** 5h**/**6a **−** 6h** were designed *via* ring-closing approach by fused five- and seven-membered ring which was first used in the design of new SMART analogues. All target compounds were synthesised using proper manners. MTT assay was used to evaluate the *in vitro* antiproliferative activities of all target compounds against SGC-7901 and MCF-7 cell lines. Additional cell lines including HCT-116, A549, PC-3 and HUVEC were brought to test the antiproliferative activities of potent compounds **4k** and **5a**. Among these compounds, **4k** and **5a** showed the highest antiproliferative activities with the IC_50_ values of 0.015 ± 0.004 and 0.006 ± 0.002 μM against PC-3 cell line, and exhibited less toxicity against human normal cell HUVEC. Molecular docking study showed that both **4k** and **5a** could bind to the colchicine binding site as the “bent” conformation and at the poses similar to DAMA-colchicine. Further molecular dynamic simulations exhibited the similarity of the binding model between tubulin and the three ligand (**4k**, **5a** and DAMA-colchicine). Prediction of physicochemical properties exhibited good drug-likeness of **4k** and **5a** without violation of Lipinski’s Rule of Five, as well as better water solubilities than SMART. *In silico* toxicity study suggested the toxicity decrease of compounds **4k** and **5a** in some certain aspects compared with SMART. At last, a series of mechanism studies showed that compounds **4k** and **5a** inhibited the polymerisation of tubulin, arrested PC-3 cells in G_2_/M phase, induced cell apoptosis, inhibited cell migration and prevented clonogenic formation of PC-3 cells. Additional western blot assays revealed that compounds **4k** and **5a** could upregulate the expression of CDK1, cyclin-B1, Bax, cleaved caspase-9, and downregulate the expression of Bcl-2. These results proved that compounds **4k** and **5a** are both potent tubulin polymerisation inhibitors. This work also demonstrated the use of ring-closing approach by fused five- and seven-membered ring, which has set a base for further research of new CBSIs.

## Experimental section

### Chemistry

All reagents and solvents were obtained from commercially available sources and used without purification. Reactions were monitored by analytical thin-layer chromatography (TLC) with silica gel plates (HSGF-254, 0.25 mm layer, Qingdao Haiyang Chemical Co. Ltd.) under UV light (wavelength: 254 nm and 365 nm). Flash column chromatography was performed using column chromatography (200–300 mesh, Qingdao Haiyang Chemical Co. Ltd.). Melting point was measured (uncorrected) on a hot-stage microscope (Beijing Taike, X-4).^1^H NMR (400 MHz) and ^13^C NMR (100 MHz) spectra were tested on a Bruker AVANCE 400 spectrometer by using DMSO-*d*_6_ or CDCl_3_ (contains 0.03%(v/v) TMS) as solvent at room temperature. High-resolution mass spectra (HRMS) were determined by a Agilent Accurate-Mass Q-TOF 6500 (Agilent Co. Ltd.) instrument equipped with electrospray ionisation (ESI).

### Synthetic procedure for 3-oxo-3–(3,4,5-trimethoxyphenyl)propanenitrile (8)

To a mixture of 3,4,5-trimethoxybenzaldehyde (**7**) (5 g, 26.31 mmol), potassium hydroxide (4.42 g, 78.78 mmol) and CuCl_2_·2H_2_O (224 mg, 1.32 mmol) in DMA (17.5 ml) was added acetonitrile (13.7 ml, 263.10 mmol), and the mixture was stirred under O_2_ atmosphere at room temperature for 24 h. The excess acetonitrile was removed under reduced pressure, and the resulting mixture was diluted with water (30 ml), then acidified by 1 mol/l HCl solution until pH below 3. The precipitate was filtered off, washed with water, then dried to obtain the crude product of 3-oxo-3–(3,4,5-trimethoxyphenyl)propanenitrile (**8**) which was used directly for the next step without any purification.

### General synthetic procedure for aryldiazonium chloride (10a − j)

To 2 ml of water in 50 ml round bottom flask was added aryl amine (**9a**−**j**) (4.06 mmol) and hydrochloric acid (1.35 ml conc. hydrochloric acid diluted to 5 ml, 16.24 mmol) dropwise under stirring at 0 °C, then NaNO_2_ solution (308 mg in 5 ml water, 4.47 mmol) was added dropwise under same condition. The solution was stirred at 0 °C for another 30 min to obtain corresponding aryldiazonium chloride solution (**10a**−**j**) which was used for the next step without any purification.

### General synthetic procedure for 2-oxo-N-aryl-2–(3,4,5-trimethoxyphenyl)acetohydrazonoyl cyanide (11a − j)

To a mixture of 3-oxo-3–(3,4,5-trimethoxyphenyl)propanenitrile (**8**) (954 mg, 4.06 mmol) and sodium acetate (1.105 g, 8.12 mmol) in 9 ml of ethanol and 3 ml of water was added aryldiazonium chloride solution (**10a**−**j**) (12 ml, 4.06 mmol) which was mentioned above dropwise under stirring at 0 °C, then the mixture was stirred at 0 °C for another 2 h. The mixture was filtered to get the yellow solid (**11a**−**j**), and the solid was washed with water to get the crude product which was used directly for the next step without any purification.

### General synthetic procedure for methyl 4-amino-1-aryl-3–(3,4,5-trimethoxybenzoyl)-1H-pyrazole-5-carboxylate (12a − j)

To a solution of 2-oxo-*N*-aryl-2–(3,4,5-trimethoxyphenyl)acetohydrazonoyl cyanide (**11a**−**j**) (2.00 mmol) in DMF (6 ml) was added potassium carbonate (622 mg, 4.50 mmol) and methyl bromoacetate (0.38 ml, 4.01 mmol), then the mixture was stirred at 130 °C for 2 h. The mixture was cooled to room temperature and poured into water (50 ml), then was extracted with ethyl acetate (20 ml × 3). The combined organic layer was washed with water (30 ml) and brine, and dried over anhydrous Na_2_SO_4_. Then the solvent was removed under reduced pressure to obtain the crude product (**12a**−**j**) which was used for the next step without any purification.

### General synthetic procedure for (4-amino-1-aryl-1H-pyrazol-3-yl)(3,4,5-trimethoxyphenyl) methanone (4a − j)

4-Amino-1-aryl-3–(3,4,5-trimethoxybenzoyl)-1*H*-pyrazole-5-carboxylate (**12a**−**j**) (1.00 mmol) was added to solution of sodium hydroxide (200 mg, 5.00 mmol) in water (10 ml), and the mixture was stirred at 80 °C for 3 h. The mixture was cooled to room temperature, then 2 M HCl was added dropwise until pH = 2, and the precipitate was formed. The precipitate was collected by filtration, washed with water, and dried to get 4-amino-1-aryl-3–(3,4,5-trimethoxybenzoyl)-1*H*-pyrazole-5-carboxylic acid which was used for the next step without purification.

4-Amino-1-aryl-3–(3,4,5-trimethoxybenzoyl)-1*H*-pyrazole-5-carboxylic acid (1.00 mmol) was added to solution of *n*-propanol (10 ml) and conc. HCl (1 ml), and the solution was stirred at 80 °C for 30 min. The solution was cooled to room temperature, and sat. NaHCO_3_ solution was added until pH = 7. The precipitate was collected by filtration, washed with water, and dried to get the crude product (**4a**−**j**) . The crude product was purified by column chromatography (petroleum ether: ethyl acetate= 3:2) to get the pure product.

(4-Amino-1-phenyl-1*H*-pyrazol-3-yl)(3,4,5-trimethoxyphenyl)methanone (**4a**). Yellow solid. Yield 61%. Mp 119–120 °C.^1^H NMR (400 MHz, CDCl_3_): δ 7.78 (s, 2H), 7.65–7.63 (m, 2H), 7.48 (s, 1H), 7.41–7.37 (m, 2H), 7.28–7.23 (m, 1H), 3.89–3.88 (m, 9H); ^13^C NMR (100 MHz, CDCl_3_) δ 187.8, 152.7 (2 C), 142.1, 139.9, 139.0, 137.8, 132.7, 129.5 (2 C), 127.1, 118.9, 112.6, 108.2 (2 C), 61.0, 56.2 (2 C).

(4-Amino-1–(3-methylphenyl)-1*H*-pyrazol-3-yl)(3,4,5-trimethoxyphenyl)methanone (**4b**). Yellow solid. Yield 61%. Mp 153–154 °C. ^1^H NMR (400 MHz, CDCl_3_): δ 7.78 (s, 2H), 7.48–7.47 (m, 1H), 7.44 (s, 1H), 7.40–7.37 (m, 1H), 7.24 (t, *J* = 7.9 Hz, 1H), 7.06–7.03 (m, 1H), 3.88–3.87 (m, 9H), 2.32 (s, 3H); ^13^C NMR (100 MHz, CDCl_3_) δ 187.7, 152.7 (2 C), 142.1, 139.9, 139.6, 138.9, 137.8, 132.8, 129.3, 127.9, 119.6, 115.9, 112.6, 108.2 (2 C), 60.9, 56.1 (2 C), 21.5.

(4-Amino-1–(4-methylphenyl)-1*H*-pyrazol-3-yl)(3,4,5-trimethoxyphenyl)methanone (**4c**). Yellow solid. Yield 68%. Mp 111–112 °C. ^1^H NMR (400 MHz, CDCl_3_): δ 7.78 (s, 2H), 7.48–7.47 (m, 1H), 7.44 (s, 1H), 7.40–7.37 (m, 1H), 7.24 (t, *J* = 7.9 Hz, 1H), 7.06–7.03 (m, 1H), 3.88–3.87 (m, 9H), 2.32 (s, 3H); ^13^C NMR (100 MHz, CDCl_3_) δ 187.7, 152.7 (2 C), 142.0, 138.7, 137.8, 137.7, 137.1, 132.8, 130.0 (2 C), 118.8 (2 C), 112.6, 108.2 (2 C), 60.9, 56.2 (2 C), 21.0.

(4-Amino-1–(2-fluorophenyl)-1*H*-pyrazol-3-yl)(3,4,5-trimethoxyphenyl)methanone (**4d**). Yellow solid. Yield 57%. Mp 158–159 °C. ^1^H NMR (400 MHz, CDCl_3_): δ 7.74 (s, 2H), 7.60–7.57 (m, 2H), 7.40 (s, 1H), 7.10–7.06 (m, 2H), 3.88–3.87 (m, 9H); ^13^C NMR (100 MHz, CDCl_3_) δ 187.8, 161.5 (d, *J* = 247.6 Hz), 152.7 (2 C), 142.2, 139.0, 138.0, 136.3 (d, *J* = 2.9 Hz), 132.7, 120.6 (d, *J* = 8.3 Hz), 116.4 (d, *J* = 23.3 Hz), 112.7, 108.2 (2 C), 60.9, 56.2 (2 C).

(4-Amino-1–(3-fluorophenyl)-1*H*-pyrazol-3-yl)(3,4,5-trimethoxyphenyl)methanone (**4e**). Yellow solid. Yield 59%. Mp 157–158 °C. ^1^H NMR (400 MHz, CDCl_3_): δ 7.76 (s, 2H), 7.46 (s, 1H), 7.43–7.32 (m, 3H), 6.98–6.93 (m, 1H), 3.89–3.88 (m, 9H); ^13^C NMR (100 MHz, CDCl_3_) δ 187.7, 163.3 (d, *J* = 247.3 Hz), 152.7 (2 C), 142.3, 141.3 (d, *J* = 10.3 Hz), 139.3, 137.9, 132.5, 130.9 (d, *J* = 9.4 Hz), 114.1 (d, *J* = 3.3 Hz), 114.0, 113.8, 108.2 (2 C), 106.6 (d, *J* = 26.8 Hz), 61.0, 56.2 (2 C).

(4-Amino-1–(4-fluorophenyl)-1*H*-pyrazol-3-yl)(3,4,5-trimethoxyphenyl)methanone (**4f**). Yellow solid. Yield 64%. Mp 143–144 °C. ^1^H NMR (400 MHz, CDCl_3_): δ 7.81 (s, 2H), 7.69–7.65 (m, 2H), 7.48 (s, 1H), 7.18–7.14 (m, 1H), 3.95–3.94 (m, 9H); ^13^C NMR (100 MHz, CDCl_3_) δ 187.8, 161.5 (d, *J* = 247.3 Hz), 152.7 (2 C), 142.2, 139.1, 138.0, 136.3 (d, *J* = 3.0 Hz), 132.7, 120.7 (d, *J* = 8.2 Hz, 2 C), 116.4 (d, *J* = 22.9 Hz, 2 C), 112.7, 108.2 (2 C), 61.0, 56.2 (2 C).

(4-Amino-1–(4-methoxyphenyl)-1*H*-pyrazol-3-yl)(3,4,5-trimethoxyphenyl)methanone (**4 g**). Yellow solid. Yield 46%. Mp 108–109 °C. ^1^H NMR (400 MHz, CDCl_3_) δ 7.83 (s, 2H), 7.63–7.57 (m, 2H), 7.48 (s, 1H), 7.00 − 6.93 (m, 2H), 4.53 (s, 2H), 3.95 (s, 9H), 3.85 (s, 3H). ^13^C NMR (100 MHz, CDCl_3_) δ 187.71, 158.77, 152.64 (2 C), 141.96, 138.59, 137.54, 133.59, 132.83, 120.42 (2 C), 114.62 (2 C), 113.00, 108.12 (2 C), 60.94, 56.19 (2 C), 55.61. HRMS calcd for C_20_H_21_N_3_O_5_ [M + Na]^+^: 406.1373, found 406.1371.

(4-Amino-1–(4-chlorophenyl)-1*H*-pyrazol-3-yl)(3,4,5-trimethoxyphenyl)methanone (**4h**). Yellow solid. Yield 62%. Mp 153–154 °C. ^1^H NMR (400 MHz, DMSO-*d*_6_): δ 7.95 (s, H), 7.90 (d, *J* = 8.9 Hz, 2H), 7.74 (s, 2H), 7.60 (d, *J* = 8.9 Hz, 2H), 3.88 (s, 6H), 3.79 (s, 3H); ^13^C NMR (100 MHz, DMSO-*d*_6_) δ 186.9, 152.8 (2 C), 141.9, 138.7, 138.5, 133.0, 131.6, 130.1 (2 C), 120.6 (2 C), 113.0, 108.2 (2 C), 60.6, 56.4 (2 C).

(4-Amino-1–(2-methylphenyl)-1*H*-pyrazol-3-yl)(3,4,5-trimethoxyphenyl)methanone (**4i**). Yellow solid. Yield 61%. Mp 92–93 °C. ^1^H NMR (400 MHz, CDCl_3_) δ 7.75 (s, 2H), 7.35–7.26 (m, 5H), 3.91 (s, 3H), 3.90 (s, 6H), 2.37 (s, 3H). ^13^C NMR (100 MHz, CDCl_3_) δ 188.21, 152.67 (2 C), 142.00, 139.80, 138.78, 136.62, 133.15, 132.96, 131.57, 128.74, 126.72, 125.62, 117.44, 108.02 (2 C), 60.89, 56.19 (2 C), 18.34. HRMS calcd for C_20_H_21_N_3_O_4_ [M + Na]^+^: 390.1424, found 390.1414.

(4-Amino-1–(4-nitrophenyl)-1*H*-pyrazol-3-yl)(3,4,5-trimethoxyphenyl)methanone (**4j**). Orange solid. Yield 66%. Mp 225–226 °C. ^1^H NMR (400 MHz, DMSO-*d*_6_) δ 8.44–8.36 (m, 2H), 8.16–8.11 (m, 2H), 8.10 (s, 1H), 7.73 (s, 2H), 3.89 (s, 6H), 3.80 (s, 3H). ^13^C NMR (100 MHz, DMSO-*d*_6_) δ 187.05, 152.88 (2 C), 145.70, 144.14, 142.06, 139.81, 139.30, 132.65, 125.99 (2 C), 119.27 (2 C), 113.22, 108.16 (2 C), 60.66, 56.43 (2 C). HRMS calcd for C_19_H_18_N_4_O_6_ [M + Na]^+^: 421.1119, found 421.1121.

### Synthetic procedure for (4-amino-1–(4-aminophenyl)-1H-pyrazol-3-yl)(3,4,5-trimethoxyphenyl)methanone (4k)

To a solution of (4-amino-1–(4-nitrophenyl)-1*H*-pyrazol-3-yl)(3,4,5-trimethoxyphenyl) methanone (**4j**) (199 mg, 0.50 mmol) in 5 ml ethanol was added SnCl_2_·2H_2_O (564 mg, 2.50 mmol), and the mixture was stirred under reflux for 3 h. The excess SnCl_2_·2H_2_O was filtered off, then the solvent was removed to obtain the crude product (**4k**) which was purified by column chromatography on silica gel (petroleum ether: ethyl acetate= 1:1) to afford the pure product. Yellow solid. Yield 81%. Mp 139–140 °C. ^1^H NMR (400 MHz, CDCl_3_) δ 7.83 (s, 2H), 7.51–7.44 (m, 2H), 7.41 (s, 1H), 6.77–6.68 (m, 2H), 3.94 (s, 9H). ^13^C NMR (100 MHz, CDCl_3_) δ 187.69, 152.62 (2 C), 145.86, 141.82, 138.15, 137.77, 132.99, 131.92, 120.53 (2 C), 115.36 (2 C), 112.73, 108.10 (2 C), 60.94, 56.17 (2 C). HRMS calcd for C_19_H_20_N_4_O_4_ [M + Na]^+^: 391.1377, found 391.1379.

### Synthetic procedure for (9H-fluoren-9-yl)methyl(2-chloro-2-oxoethyl)carbamate (14)

(((9H-fluoren-9-yl)methoxy)carbonyl)glycine (**13**) (743 mg, 2.50 mmol) was added to 20 ml of anhydrous dichloromethane, then SOCl_2_ (1 ml, 13.77 mmol) was added, and the mixture was stirred at 38 °C for 2 h. The solvent and excess SOCl_2_ was evaporated *in vacuo* to obtain the crude product (**14**) which was used for the next step without purification.

### General synthetic procedure for 2-amino-N-(1-aryl-3–(3,4,5-trimethoxybenzoyl)-1H-pyrazol-4-yl) acetamide (15a − h)

(4-amino-1-aryl-1*H*-pyrazol-3-yl)(3,4,5-trimethoxyphenyl) methanone (**4a**−**h**) (0.25 mmol) was added to 5 ml of dichloromethane, then (9*H*-fluoren-9-yl)methyl(2-chloro-2-oxoethyl)carbamate (**14**) (118 mg, 0.38 mmol) was added to the solution. The mixture was stirred for 2 h at room temperature and the progress was monitored by TLC. When the reaction was complete, 0.5 ml of piperidine was added to the mixture, and the mixture was stirred for another 30 min. The mixture was added water (30 ml), and was extracted with dichloromethane(15 ml × 3). The combined organic layer was washed with water (30 ml) and brine, dried over anhydrous Na_2_SO_4_, and evaporated *in vacuo* to obtain the crude product (**15a**−**h**) which was used for the next step without purification.

### General synthetic procedure for 2-aryl-8–(3,4,5-trimethoxyphenyl)-2,6-dihydropyrazolo[4,3-e][1,4]diazepin-5(4H)-one (5a − h)

2-Amino-*N*-(1-aryl-3–(3,4,5-trimethoxybenzoyl)-1*H*-pyrazol-4-yl) acetamide (**15a**−**h**) (0.25 mmol) was added to 5 ml solution of 5% AcOH in EtOH, and the solution was stirred under reflux for 3 h. Then 20 ml of saturated NaHCO_3_ was added to quench the reaction, and EtOH was evaporated *in vacuo*. The mixture was extracted with dichloromethane (10 ml × 3), and the combined layer was washed with brine and dried over anhydrous Na_2_SO_4_. The solvent was removed *in vacuo* to get the crude product (**5a**−**h**), and the crude product was purified by column chromatography (dichloromethane: ethyl acetate= 3:1) to get the pure product.

2-Phenyl-8–(3,4,5-trimethoxyphenyl)-2,6-dihydropyrazolo[4,3-*e*][1,4]diazepin-5(4*H*)-one (**5a**). Light- yellow solid. Yield 73%. Mp 241–242 °C. ^1^H NMR (400 MHz, CDCl_3_) δ 9.23 (s, 1H), 7.94 (s, 1H), 7.76–7.70 (m, 2H), 7.50 (t, *J* = 7.9 Hz, 2H), 7.38 (t, *J* = 7.4 Hz, 1H), 7.32 (s, 2H), 4.53 (s, 2H), 3.90 (s, 3H), 3.88 (s, 6H). ^13^C NMR (100 MHz, DMSO-*d*_6_) δ 167.84, 162.90, 152.79 (2 C), 140.56, 140.11, 139.62, 132.67, 130.18 (2 C), 127.82, 127.62, 119.25 (2 C), 118.16, 106.97 (2 C), 60.52 (d, *J* = 3.3 Hz), 57.99, 56.25 (d, *J* = 3.4 Hz, 2 C). HRMS calcd for C_21_H_20_N_4_O_4_ [M + Na]^+^: 415.1377, found 415.1395.

2–(3-Methylphenyl)-8–(3,4,5-trimethoxyphenyl)-2,6-dihydropyrazolo[4,3-*e*][1,4]diazepin-5(4*H*)-one (**5b**). Light-yellow solid. Yield 73%. Mp 235–236 °C. ^1^H NMR (400 MHz, CDCl_3_) δ 9.81 (s, 1H), 7.94 (s, 1H), 7.56 (s, 1H), 7.50 (dd, *J* = 8.1, 2.2 Hz, 1H), 7.36 (t, *J* = 7.9 Hz, 1H), 7.32 (s, 2H), 7.18 (d, *J* = 7.6 Hz, 1H), 4.53 (s, 2H), 3.90 (s, 3H), 3.88 (s, 6H), 2.42 (s, 3H). ^13^C NMR (100 MHz, CDCl_3_) δ 169.56, 164.44, 152.83 (2 C), 140.77, 140.54, 139.94, 139.39, 132.48, 129.52, 128.63, 126.48, 120.00, 117.18, 116.36, 107.07 (2 C), 60.91, 57.25, 56.19 (2 C), 21.49. HRMS calcd for C_22_H_22_N_4_O_4_ [M + Na]^+^: 429.1533, found 429.1550.

2–(4-Methylphenyl)-8–(3,4,5-trimethoxyphenyl)-2,6-dihydropyrazolo[4,3-*e*][1,4]diazepin-5(4*H*)-one (**5c**). Light-yellow solid. Yield 75%. Mp 226–227 °C. ^1^H NMR (400 MHz, CDCl_3_) δ 9.89 (s, 1H), 7.92 (s, 1H), 7.60 (d, *J* = 8.1 Hz, 2H), 7.31 (s, 2H), 7.27 (d, *J* = 8.4 Hz, 2H), 4.52 (s, 2H), 3.89 (s, 3H), 3.87 (s, 6H), 2.40 (s, 3H). ^13^C NMR (100 MHz, CDCl_3_) δ 169.58, 164.48, 152.81 (2 C), 140.61, 140.48, 137.87, 137.18, 132.55, 130.23 (2 C), 126.44, 119.14 (2 C), 117.08, 107.05 (2 C), 60.90, 57.27, 56.19 (2 C), 21.01. HRMS calcd for C_22_H_22_N_4_O_4_ [M + Na]^+^: 429.1533, found 429.1552.

2–(2-Fluorophenyl)-8–(3,4,5-trimethoxyphenyl)-2,6-dihydropyrazolo[4,3-*e*][1,4]diazepin-5(4*H*)-one (**5d**). Light-yellow solid. Yield 72%. Mp 227–228 °C. ^1^H NMR (400 MHz, CDCl_3_) δ 9.55 (s, 1H), 8.00 (d, *J* = 2.1 Hz, 1H), 7.84 (td, *J* = 7.9, 1.6 Hz, 1H), 7.38 (dddd, *J* = 8.8, 6.7, 4.8, 1.8 Hz, 1H), 7.32–7.25 (m, 4H), 4.53 (s, 2H), 3.90 (s, 3H), 3.89 (s, 6H). ^13^C NMR (100 MHz, CDCl_3_) δ 169.41, 164.20, 155.25, 152.84 (2 C), 152.76, 140.75 (d, J = 39.6 Hz), 132.48, 129.37 (d, *J* = 8.0 Hz), 127.70 (d, *J* = 9.2 Hz), 126.20, 125.20 (d, *J* = 3.8 Hz), 124.38, 121.03 (d, *J* = 9.9 Hz), 117.25 (d, *J* = 20.2 Hz), 106.97 (2 C), 60.90, 57.19, 56.19 (2 C). HRMS calcd for C_21_H_19_FN_4_O_4_ [M + Na]^+^: 433.1283, found 433.1301.

2–(3-Fluorophenyl)-8–(3,4,5-trimethoxyphenyl)-2,6-dihydropyrazolo[4,3-*e*][1,4]diazepin-5(4*H*)-one (**5e**). Light-yellow solid. Yield 71%. Mp 228–229 °C. ^1^H NMR (400 MHz, CDCl_3_) δ 9.87 (s, 1H), 7.96 (s, 1H), 7.54–7.41 (m, 3H), 7.29 (s, 2H), 7.10–7.04 (m, 1H), 4.53 (s, 2H), 3.90 (s, 3H), 3.87 (s, 6H). ^13^C NMR (100 MHz, CDCl_3_) δ 169.57, 164.50, 164.27, 162.04, 152.86 (2 C), 141.26, 140.76, 140.66, 140.62, 132.35, 131.19, 131.10, 126.78, 117.16, 114.78, 114.57, 114.41, 107.15, 107.00 (2 C), 106.89, 60.92, 57.24, 56.22 (2 C). HRMS calcd for C_21_H_19_FN_4_O_4_ [M + Na]^+^: 433.1283, found 433.1298.

2–(4-Fluorophenyl)-8–(3,4,5-trimethoxyphenyl)-2,6-dihydropyrazolo[4,3-*e*][1,4]diazepin-5(4*H*)-one (**5f**). Light-yellow solid. Yield 73%. Mp 212–213 °C. ^1^H NMR (400 MHz, CDCl_3_) δ 9.80 (s, 1H), 7.92 (s, 1H), 7.73–7.66 (m, 2H), 7.27 (s, 2H), 7.21–7.14 (m, 2H), 4.52 (s, 2H), 3.90 (s, 3H), 3.87 (s, 6H). ^13^C NMR (100 MHz, CDCl_3_) δ 169.48, 164.38, 163.11, 160.64, 152.85 (2 C), 140.81 (d, *J* = 40.3 Hz), 135.73 (d, *J* = 3.2 Hz), 132.45, 126.64, 121.11 (d, *J* = 8.4 Hz, 2 C), 117.24, 116.66 (d, *J* = 23.1 Hz, 2 C), 107.07 (2 C), 60.92, 57.24, 56.23 (2 C). HRMS calcd for C_21_H_19_FN_4_O_4_ [M + Na]^+^: 433.1283, found 433.1298.

2–(4-Methoxyphenyl)-8–(3,4,5-trimethoxyphenyl)-2,6-dihydropyrazolo[4,3-*e*][1,4]diazepin-5(4*H*)-one (**5 g**). Light-yellow solid. Yield 72%. Mp 164–165 °C. ^1^H NMR (400 MHz, CDCl_3_) δ 9.06 (s, 1H), 7.84 (s, 1H), 7.65–7.59 (m, 2H), 7.31 (s, 2H), 7.02–6.96 (m, 2H), 4.52 (s, 2H), 3.90 (s, 3H), 3.88 (s, 6H), 3.86 (s, 3H). ^13^C NMR (100 MHz, CDCl_3_) δ 169.17, 164.35, 159.24, 152.81 (2 C), 140.51, 133.01, 132.55, 126.27, 120.88 (2 C), 117.01, 114.79 (2 C), 107.04 (2 C), 60.91, 57.18, 56.22 (2 C), 55.65. HRMS calcd for C_22_H_22_N_4_O_5_ [M + Na]^+^: 445.1482, found 445.1501.

2–(4-Chlorophenyl)-8–(3,4,5-trimethoxyphenyl)-2,6-dihydropyrazolo[4,3-*e*][1,4]diazepin-5(4*H*)-one (**5h**). Light-yellow solid. Yield 71%. Mp 189–190 °C. ^1^H NMR (400 MHz, CDCl_3_) δ 9.66 (s, 1H), 7.94 (s, 1H), 7.70–7.65 (m, 2H), 7.48–7.43 (m, 2H), 7.28 (s, 2H), 4.52 (s, 2H), 3.90 (s, 3H), 3.87 (s, 6H). ^13^C NMR (100 MHz, CDCl_3_) δ 169.48, 164.30, 152.85 (2 C), 141.18, 140.63, 137.91, 133.53, 132.36, 129.89 (2 C), 126.73, 120.33 (2 C), 117.00, 107.04 (2 C), 60.93, 57.20, 56.23 (2 C). HRMS calcd for C_21_H_19_ClN_4_O_4_ [M + Na]^+^: 449.0987, found 449.1000.

### General synthetic procedure for (4-isothiocyanato-1-aryl-1H-pyrazol-3-yl)(3,4,5-trimethoxyphenyl)methanone (16a − h)

To the solution of (4-amino-1-aryl-1*H*-pyrazol-3-yl)(3,4,5-trimethoxyphenyl)methanone (**4a**−**h**) (1.00 mmol) in 5 ml dichloromethane was added 5 ml of saturated NaHCO_3_, then thiophosgene (0.091 μL, 1.19 mmol) was slowly added to the mixture under vigorous stirring at room temperature. Then the reaction continued stirring for another 1 h. The organic layer was separated, washed with water (10 ml) and brine, and then concentrated *in vacuo* to get the crude product (**16a**−**h**) which was used for the next step without purification.

### General synthetic procedure for N-(3-(hydrazineylidene(3,4,5-trimethoxyphenyl)methyl)-1-aryl-1H-pyrazol-4-yl)hydrazinecarbothioamide (17a − h)

To the solution of (4-isothiocyanato-1-aryl-1*H*-pyrazol-3-yl)(3,4,5-trimethoxyphenyl)methanone (**16a**−**h**) (1.00 mmol) in 5 ml ethanol was added hydrazine hydrate (80%, 1.2 ml, 19.79 mmol), and the solution was stirred under reflux for 3 h. Then the solvent was removed under vacuum, then the residue was co-evaporated with toluene (7 ml × 4) to get the crude product (**17a**−**h**) which was used for the next step without purification.

### General synthetic procedure for 2-aryl-8–(3,4,5-trimethoxyphenyl)-2,6-dihydropyrazolo[4,3-e][1,2,4]triazepine-5(4H)-thione (6a − h)

To the solution of TsOH·H_2_O (228 mg, 1.20 mmol) in 10 ml of ethanol was added *N*-(3-(hydrazineylidene(3,4,5-trimethoxyphenyl)methyl)-1-aryl-1*H*-pyrazol-4-yl)hydrazinecarbothioamide (**17a**−**h**) (1.00 mmol), and the solution was stirred under reflux for 3 h. The reaction was cooled to room temperature, then was added 25 ml of water, and saturated NaHCO_3_ was added dropwise under stirring until pH > 7. The mixture was filtered, and the precipitate was washed with water and dried to get crude product (**6a**−**h**). The crude product was purified by column chromatography (dichloromethane: ethyl acetate= 5:1) to obtain the pure product.

2-Phenyl-8–(3,4,5-trimethoxyphenyl)-2,6-dihydropyrazolo[4,3-*e*][1,2,4]triazepine-5(4*H*)-thione (**6a**). Light-yellow solid. Yield 23%. Mp 194–195 °C. ^1^H NMR (400 MHz, CDCl_3_) δ 9.23 (s, 1H), 7.94 (s, 1H), 7.76–7.70 (m, 2H), 7.50 (t, *J* = 7.9 Hz, 2H), 7.38 (t, *J* = 7.4 Hz, 1H), 7.32 (s, 2H), 4.53 (s, 2H), 3.90 (s, 3H), 3.88 (s, 6H). ^13^C NMR (100 MHz, DMSO-*d*_6_) δ 186.13, 153.17, 152.82 (2 C), 139.67, 139.35, 138.69, 130.63, 130.17 (2 C), 128.78, 127.77, 119.03 (2 C), 117.51, 106.81 (2 C), 60.59, 56.38 (2 C). HRMS calcd for C_20_H_19_N_5_O_3_S [M + Na]^+^: 432.1101, found 432.1115.

2–(3-Methylphenyl)-8–(3,4,5-trimethoxyphenyl)-2,6-dihydropyrazolo[4,3-*e*][1,2,4]triazepine-5(4*H*)-thione (**6b**). Light-yellow solid. Yield 21%. Mp 221–222 °C. ^1^H NMR (400 MHz, DMSO-*d*_6_) δ 10.70 (d, *J* = 2.0 Hz, 1H), 10.33 (d, *J* = 2.1 Hz, 1H), 7.94 (s, 1H), 7.59 (t, *J* = 1.9 Hz, 1H), 7.55 (dd, *J* = 8.0, 2.3 Hz, 1H), 7.39 (t, *J* = 7.8 Hz, 1H), 7.19 (d, *J* = 4.9 Hz, 3H), 3.80 (s, 6H), 3.74 (s, 3H), 2.37 (s, 3H). ^13^C NMR (100 MHz, DMSO-*d*_6_) δ 186.12, 153.13, 152.81 (2 C), 139.81, 139.68, 139.34, 138.55, 130.61, 129.98, 128.72, 128.43, 119.48, 117.47, 116.20, 106.82 (2 C), 60.59, 56.34 (2 C), 21.44. HRMS calcd for C_21_H_21_N_5_O_3_S [M + Na]^+^: 446.1257, found 446.1273.

2–(4-Methylphenyl)-8–(3,4,5-trimethoxyphenyl)-2,6-dihydropyrazolo[4,3-*e*][1,2,4]triazepine-5(4*H*)-thione (**6c**). Light-yellow solid. Yield 22%. Mp 214–215 °C. ^1^H NMR (400 MHz, DMSO-*d*_6_) δ 10.66 (d, *J* = 2.1 Hz, 1H), 10.31 (d, *J* = 2.1 Hz, 1H), 7.92 (s, 1H), 7.64 (d, *J* = 8.4 Hz, 2H), 7.31 (d, *J* = 8.2 Hz, 2H), 7.16 (s, 2H), 3.79 (s, 6H), 3.73 (s, 3H), 2.34 (s, 3H). ^13^C NMR (100 MHz, DMSO-*d*_6_) δ 186.23, 153.29, 152.81 (2 C), 139.67, 138.36, 137.28, 137.16, 130.64, 130.53 (2 C), 128.70, 118.93 (2 C), 117.39, 106.81 (2 C), 60.59, 56.36 (2 C), 20.93. HRMS calcd for C_21_H_21_N_5_O_3_S [M + Na]^+^: 446.1257, found 446.1278.

2–(2-Fluorophenyl)-8–(3,4,5-trimethoxyphenyl)-2,6-dihydropyrazolo[4,3-*e*][1,2,4]triazepine-5(4*H*)-thione (**6d**). Light-yellow solid. Yield 17%. Mp 154–155 °C. ^1^H NMR (400 MHz, DMSO-*d*_6_) δ 10.59 (s, 1H), 10.40 (s, 1H), 7.79–7.71 (m, 2H), 7.53–7.45 (m, 2H), 7.37 (ddd, *J* = 8.4, 4.7, 3.1 Hz, 1H), 7.13 (s, 2H), 3.78 (s, 6H), 3.72 (s, 3H). ^13^C NMR (100 MHz, DMSO-*d*_6_) δ 186.07, 155.37, 152.95, 152.81 (2 C), 139.67, 138.86, 130.62, 130.20 (d, *J* = 8.2 Hz), 128.34, 127.62 (d, *J* = 9.2 Hz), 125.97 (d, *J* = 3.7 Hz), 125.11, 121.03 (d, *J* = 7.3 Hz), 117.67 (d, *J* = 20.0 Hz), 106.82 (2 C), 60.57, 56.35 (2 C). HRMS calcd for C_20_H_18_FN_5_O_3_S [M + Na]^+^: 450.1007, found 450.1017.

2–(3-Fluorophenyl)-8–(3,4,5-trimethoxyphenyl)-2,6-dihydropyrazolo[4,3-*e*][1,2,4]triazepine-5(4*H*)-thione (**6e**). Light-yellow solid. Yield 19%. Mp 108–109 °C. ^1^H NMR (400 MHz, DMSO-*d*_6_) δ 10.76 (s, 1H), 10.39 (s, 1H), 8.04 (s, 1H), 7.71–7.62 (m, 2H), 7.56 (td, *J* = 8.2, 6.2 Hz, 1H), 7.25–7.19 (m, 1H), 7.16 (s, 2H), 3.79 (s, 6H), 3.74 (s, 3H). ^13^C NMR (100 MHz, DMSO-*d*_6_) δ 185.99, 164.15, 161.72, 152.83, 152.80 (2 C), 140.73 (d, *J* = 10.5 Hz), 139.67, 139.09, 132.04 (d, *J* = 9.3 Hz), 130.50, 128.84, 117.79, 114.92, 114.32 (d, *J* = 20.9 Hz), 106.75 (2 C), 106.41 (d, *J* = 26.8 Hz), 60.56 (d, *J* = 4.0 Hz), 56.34 (d, *J* = 4.4 Hz, 2 C).

2–(4-Fluorophenyl)-8–(3,4,5-trimethoxyphenyl)-2,6-dihydropyrazolo[4,3-*e*][1,2,4]triazepine-5(4*H*)-thione (**6f**). Light-yellow solid. Yield 18%. Mp 196–197 °C. ^1^H NMR (400 MHz, DMSO-*d*_6_) δ 10.73–10.67 (s, 1H), 10.34 (s, 1H), 7.95 (s, 1H), 7.84–7.76 (m, 2H), 7.37 (t, *J* = 8.8 Hz, 2H), 7.14 (s, 2H), 3.78 (s, 6H), 3.73 (s, 3H). ^13^C NMR (100 MHz, DMSO-*d*_6_) δ 186.23, 153.21, 152.82 (2 C), 139.68, 138.73, 135.96, 130.61, 128.81 (2 C), 121.35, 121.26, 117.81, 116.94 (d, *J* = 23.1 Hz, 2 C), 106.79 (2 C), 60.59, 56.39 (2 C).

2–(4-Methoxyphenyl)-8–(3,4,5-trimethoxyphenyl)-2,6-dihydropyrazolo[4,3-*e*][1,2,4]triazepine-5(4*H*)-thione (**6 g**). Light-yellow solid. Yield 20%. Mp 186–187 °C. ^1^H NMR (400 MHz, DMSO-*d*_6_) δ 10.44 (s, 1H), 10.27 (s, 1H), 7.86 (s, 1H), 7.70–7.64 (m, 2H), 7.15 (s, 2H), 7.09–7.04 (m, 2H), 3.79 (d, *J* = 2.0 Hz, 9H), 3.73 (s, 3H). ^13^C NMR (100 MHz, DMSO-*d*_6_) δ 186.38, 158.86, 153.46, 152.80 (2 C), 139.66, 138.10, 132.94, 130.67, 128.65, 120.74 (2 C), 117.53, 115.19 (2 C), 106.80 (2 C), 60.58, 56.37 (2 C), 55.95. HRMS calcd for C_21_H_21_N_5_O_4_S [M + Na]^+^: 462.1206, found 462.1225.

2–(4-Chlorophenyl)-8–(3,4,5-trimethoxyphenyl)-2,6-dihydropyrazolo[4,3-*e*][1,2,4]triazepine-5(4*H*)-thione (**6h**). Light-yellow solid. Yield 21%. Mp 152–153 °C. ^1^H NMR (400 MHz, DMSO-*d*_6_) δ 10.37 (s, 1H), 7.99 (s, 1H), 7.83–7.77 (m, 2H), 7.60–7.55 (m, 2H), 7.13 (s, 2H), 3.78 (s, 6H), 3.73 (s, 3H). ^13^C NMR (100 MHz, DMSO-*d*_6_) δ 186.03, 152.98, 152.83 (2 C), 139.68, 138.98, 138.19, 131.86, 130.58, 130.09 (2 C), 128.89, 120.69 (2 C), 117.62, 106.78 (2 C), 60.59, 56.39 (2 C).

### Biology

#### Cell culture

All cell lines tested in this study were purchased from the American Type Culture Collection (ATCC, Manassas, VA). SGC-7901 (human gastric adenocarcinoma), HCT-116 (human colon carcinoma), and A549 (human pulmonary carcinoma) cells were cultured in RPMI-1640 medium containing 10% foetal serum (FBS) and 1% penicillin-streptomycin solution (100×). MCF-7 (human breast carcinoma) cells were cultured in DMEM medium containing 10% foetal serum (FBS) and 1% penicillin-streptomycin solution (100×). PC-3 (human prostate carcinoma) cells were cultured in Ham’s F-12K medium containing 10% foetal serum (FBS) and 1% penicillin-streptomycin solution (100×). All cells were cultured at 37 °C in a humidified atmosphere containing 5% CO_2_.

#### MTT assay

Cells grown in logarithmic phase were harvested and seeded onto 96-well cell plates at a density of 4 ∼ 5 × 10^3^ cells per well (various from cell type) and were cultured for 24 h. Different concentrations of compounds were added into the wells, and the cells were cultured for another 72 h. The medium was removed, then 20 μL of MTT (5 mg/mL in PBS) was added to each well and the cells were incubated for 4 h. 150 μL of DMSO was added to each well to dissolve the formazan, and the absorbance at 490 nm of each well was measured by a microplate reader. IC_50_ values were calculated by SPSS software.

#### In vitro tubulin polymerisation assay

The BK001 kit used in this test was purchased from Cytoskeleton Inc. (Denver, CO, USA). Compounds **4k** or **5a** with a range of concentrations, paclitaxel, colchicine or vehicle was treated with a solution of tubulin protein in ice-cold G-PEM buffer (80 mM PIPES, 2 mM MgCl_2_, 0.5 mM EGTA, 1 mM GTP, and 15% glycerol). Then the mixtures were transferred to 37 °C and tubulin assembly was monitored (excitation wavelength: 360 nm; emission wavelength: 420 nm) at 1 min intervals for 90 min by a microplate reader.

#### Immunofluorescence assay

PC-3 cells in logarithmic phase were harvested and seeded onto 12-well cell plates with round coverslip on the bottom of each well at a density of 3 × 10^4^ cells per well, and were cultured for 24 h. Then compounds **4k** or **5a** of different concentrations, colchicine or vehicle was added into the wells, and cells were incubated for another 24 h. The medium was removed, and cells were washed with PBS twice, fixed with 4% formaldehyde in PBS for 30 min, then the solution was removed. Cells were washed with PBS twice, permeabilized with 0.2% (v/v) Triton X-100 in PBS for 30 min, then the solution was removed. Cells were washed with PBS twice, blocking the cells in 5% bovine serum albumin (BSA) for 30 min. Then BSA was removed, and cells were incubated with the primary α-tubulin antibody (Santa Cruz, CA) containing 2% BSA (v/v, 1: 100) overnight at 4 °C.

The following steps were operated in dark environment. The antibody was removed, then cells were washed with PBST twice and were added FITC-conjugated anti-mouse secondary antibody diluted (1:100) with 2% BSA in PBS and incubated at room temperature for 4 h. The cells were then washed with PBST twice, stained with 4′,6-diamidino-2-phenylindole (DAPI) for 30 min at room temperature, and washed with PBS twice. The round coverslips at the bottom of wells were taken out, and the images were photographed by a confocal microscope.

#### Cell cycle analysis

PC-3 cells in logarithmic phase were harvested and seeded onto 6-well cell plates at a density of 2.5 × 10^5^ cells per well, and were cultured for 24 h. Then compounds **4k** or **5a** of different concentrations, colchicine or vehicle was added into the wells, and cells were incubated for another 24 h. The cells were digested using trypsin and the suspended cells were harvested. Then the cells were washed with PBS and fixed in 75% ice-cold ethanol at 4 °C overnight. The fixed cells were harvested by centrifugation and washed with PBS, resuspended in 535 μL buffer containing 10 μL RNase (50 ×) and 25 μL PI (20 ×). After incubation at 37 °C in the dark for 30 min, the samples were filtered by nylon screen and detected by FACScan flow cytometry (BectoneDickinson, Franklin Lakes, NJ, USA).

#### Cell apoptosis analysis

PC-3 cells in logarithmic phase were harvested and seeded onto 6-well cell plates at a density of 2.5 × 10^5^ cells per well, and were cultured for 24 h. Then compounds **4k** or **5a** of different concentrations, colchicine or vehicle was added into the wells, and cells were incubated for another 48 h. The cells were digested using trypsin and were harvested, washed twice with PBS, and resuspended in 500 μL binding buffer, followed by the addition of 100 μL of the cell suspension to 5 μL of Annexin V-FITC and 5 μL of PI. After stained for 15 min in a dark at room temperature, the samples were added 400 μL of binding buffer and detected by FACScan flow cytometry (BectoneDickinson, Franklin Lakes, NJ, USA).

#### Western blot assay

PC-3 cells in logarithmic phase were harvested and seeded onto culture plates, and were cultured for 24 h. Then compounds **4k** or **5a** of different concentrations, colchicine or vehicle was added into the wells, and cells were incubated for another 48 h. The proteins of PC-3 cells were extracted by RIPA buffer containing 1% PMSF (Beyotime Biotechnology, China). The protein concentration was measured by BCA Assay Kit (SEVEN Biotech, China). Equal amounts and concentration of protein were diluted in 5× loading buffer (Beyotime, China) and denatured by boiled for 5 min. Protein samples were separated on 12.5% SDS-PAGE gels (EpiZyme, China) and transferred to PVDF membranes (Millipore Corporation, Billerica, MA, USA). The membranes were blocked in QuickBlockTM Block Buffer (SEVEN Biotech, China) for 10 min at room temperature. Then the membranes were incubated with the primary antibodies overnight at 4 °C, including Cyclin B1 (1:2000, Proteintech, China), CDK1 (1:5000, Proteintech, China), BAX (1:5000, Proteintech, China), Bcl2 (1:1000, Proteintech, China), Caspase 9/p35/p10 (1:1000, Proteintech, China), and β-actin (1:20 000, Proteintech, China). The membranes were then incubated with HRP (1:10 000, Proteintech, China) secondary antibodies for 1 h. The protein signals were detected by using chemiluminescent reagents kit (NCM Biotech, China) and visualised with ChemiDocImaging System (Tanon 5200, China). Protein expressions was quantified by using Fiji/ImageJ software.

#### Wound healing assay

PC-3 cells in logarithmic phase were harvested and seeded onto 12-well cell plates at a density of 2.5 × 10^5^ cells per well, and were cultured for 24 h. Scratches were made in confluent monolayers using 200 μL pipette tip, and the cells were washing twice with PBS to remove all the non-adherent cells. Compounds **4k** or **5a** of different concentrations, colchicine or vehicle was added into the wells, and cells were incubated in FBS-free medium for another 24 h. Images of wounds were taken at 0 h and 24 h and analysed by Fiji/ImageJ software.

#### Transwell migration assay

PC-3 cells in logarithmic phase were harvested and seeded onto six-well cell plates at a density of 4 × 10^4^ cells per well, and were cultured for 24 h. Subsequently compounds **4k** or **5a** of different concentrations, colchicine or vehicle was added into the wells, and cells were incubated for another 24 h. Then these cells were harvested and seeded onto the top chambers of transwell filters (Corning) at a concentration of 4 × 10^4^ cells with 200 μL of FBS-free medium per well in 24-well culture plates, while the bottom chambers were added 600 μL of medium containing 10% FBS. The cells were cultured for additional 24 h, then the cells that migrated to the bottom of the top chambers were fixed with 4% paraformaldehyde and subsequently stained with crystal violet solution. Images were taken by an inverted microscope and analysed by Fiji/ImageJ software.

#### Colony formation assay

PC-3 cells in logarithmic phase were harvested and seeded onto 12-well cell plates at a density of 1 × 10^3^ cells per well. After culture for 24 h, the cells were treated with compounds **4k** or **5a** of different concentrations, colchicine or vehicle, and were cultured for another 24 h. Then the medium was removed, and cells were washed with PBS and added fresh medium containing 10% FBS. The cells were continued to culture for one week. The medium was removed, and the cells were fixed with 4% paraformaldehyde and subsequently stained with crystal violet solution. Images were taken and analysed by Fiji/ImageJ software.

#### Molecular docking

The crystal structure of tubulin was downloaded from the Protein Data Bank (PDB: 1SA0, http://www.rcsb.org/). Maestro 11.5 software was used in molecular docking. Protein was imported, preprocessed and refined by protein preparation wizard. Ligand molecule was prepared using LigPrep. The grids were generated with Glide. Docking of conformational ensembles was performed in standard precision modes with Glide.

#### Molecular dynamic simulation study

Molecular dynamic simulation study was carried out on Maestro 13.9 software. Desmond application was used for the docked complex between tubulin (PDB: 1SA0, A and B chains) and three ligands (**4k**, **5a**, and DAMA-colchicine). The OPLS4 forcefield was used, and an orthorhombic shaped boundary box was applied. The simple point charge (SPC) solvent model was employed and 27 Na^+^ ions were added by replacing water molecules to neutralise the overall charge. Subsequently, each binding model was energy minimised. NPT ensemble was set, then the temperature and pressure were set to 300.0 K and 1.01325 bar. Finally, molecular dynamic simulation was performed with simulation time of 100 ns and recording trajectory interval of 100.0 ps. Plots were obtained by using simulation interactions diagram task.

## Supplementary Material

Supplemental material_Revised_Anonymous.docx

## Data Availability

Data will be made available from the corresponding author on reasonable request.
