# Supplementary material for: Design, synthesis, and bioevaluation of pyrazole-containing tubulin polymerisation inhibitors based on conformational constraint strategy
Source: J Enzyme Inhib Med Chem. 2025 Sep 23;40(1):2545004. doi: 10.1080/14756366.2025.2545004 (PMC12459184; doi:10.1080/14756366.2025.2545004)
Supplement: Supplemental material_Revised_Anonymous.docx [file IENZ_A_2545004_SM6049.docx]

**Design, synthesis and bioevaluation of pyrazole-containing tubulin polymerization inhibitors based on conformational constraint strategy**

**Content**  **Page**

^1^H NMR and ^13^C NMR spectra of **4a**−**4k S2**−**S12**

^1^H NMR and ^13^C NMR spectra of **5a**−**5h S13**−**S20**

^1^H NMR and ^13^C NMR spectra of **6a**−**6h S21**−**S28**

HRMS spectra of **4k** and **5a** **S29**

**Contents: ^1^H NMR and ^13^C NMR spectra of 4a**−**4k**


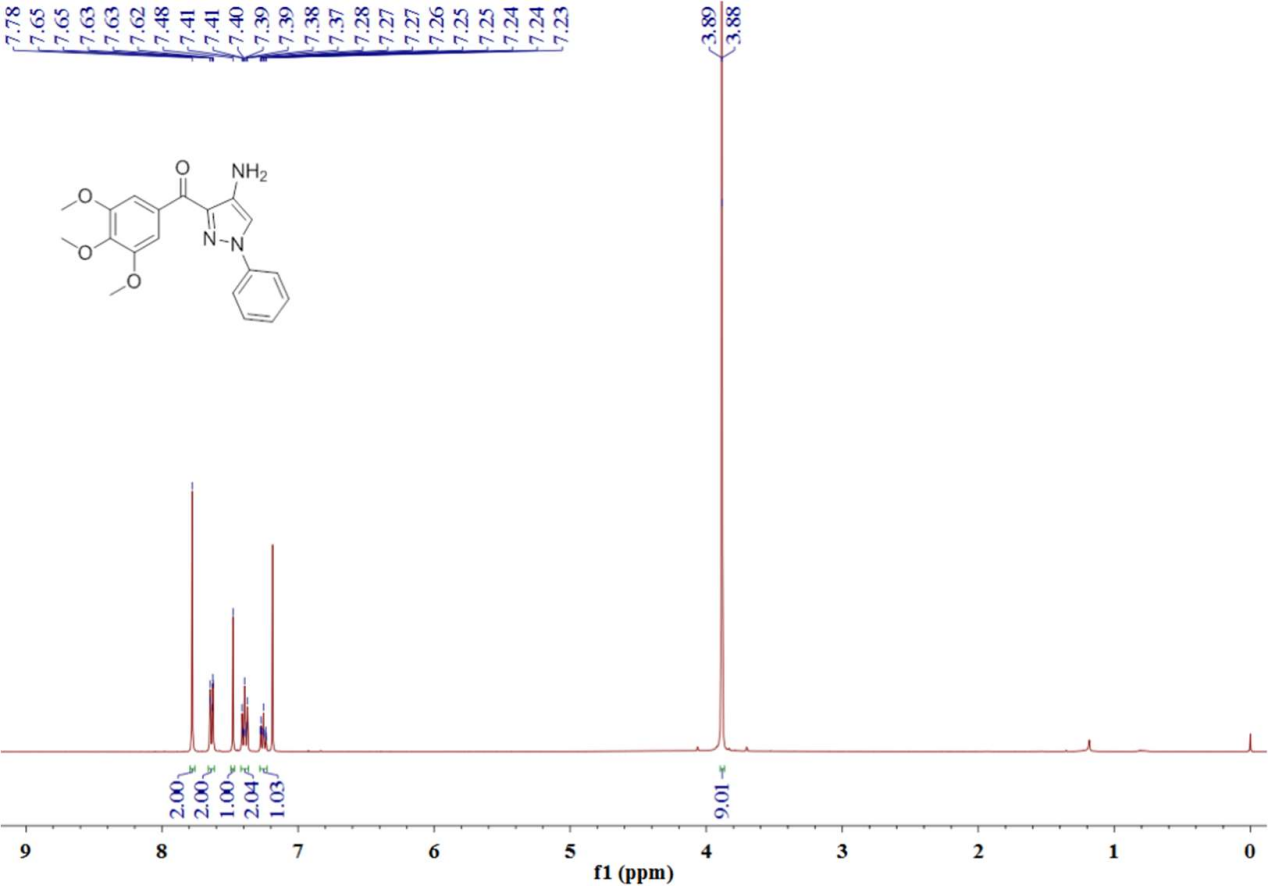


Fig. S1. ^1^H-NMR spectrum of **4a**.


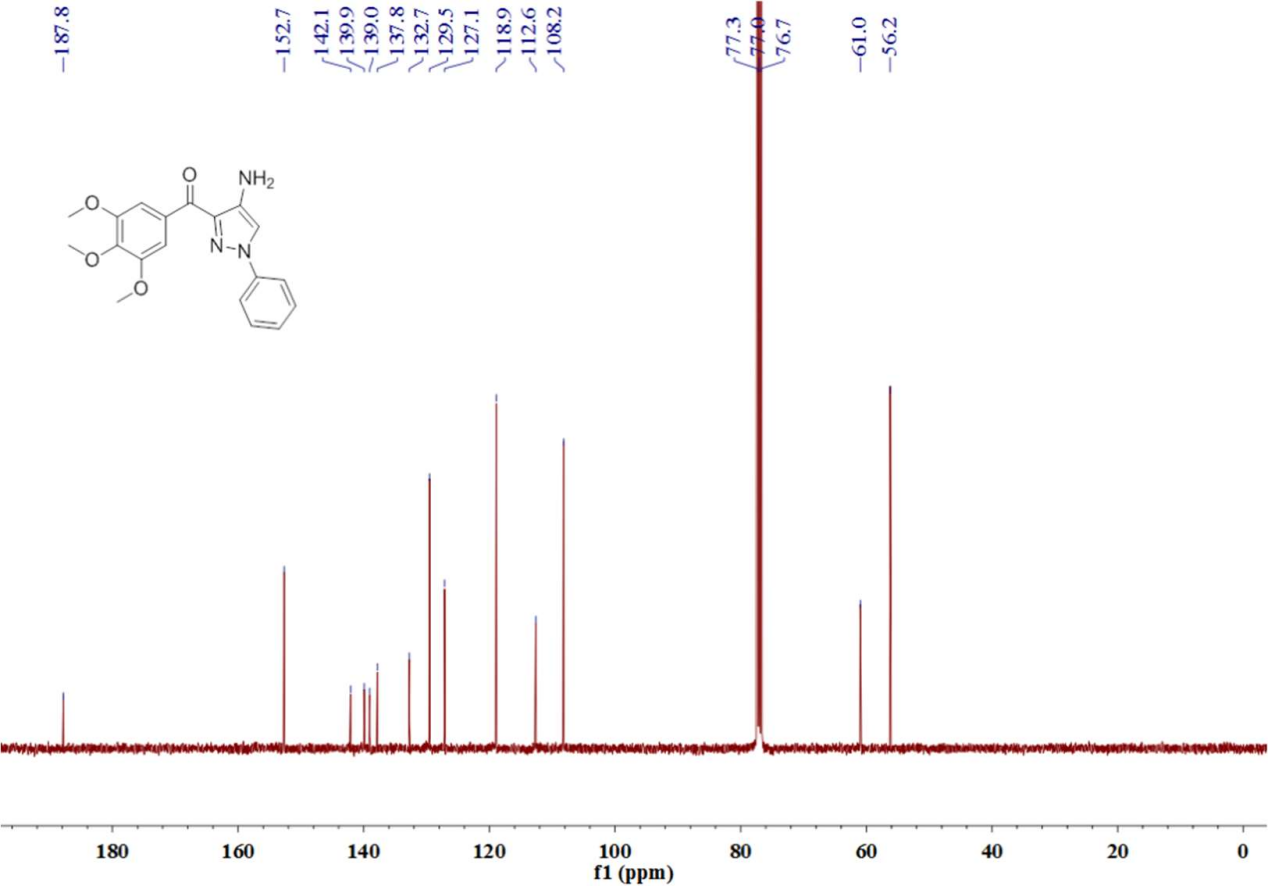


Fig. S2. ^13^C-NMR spectrum of **4a**.


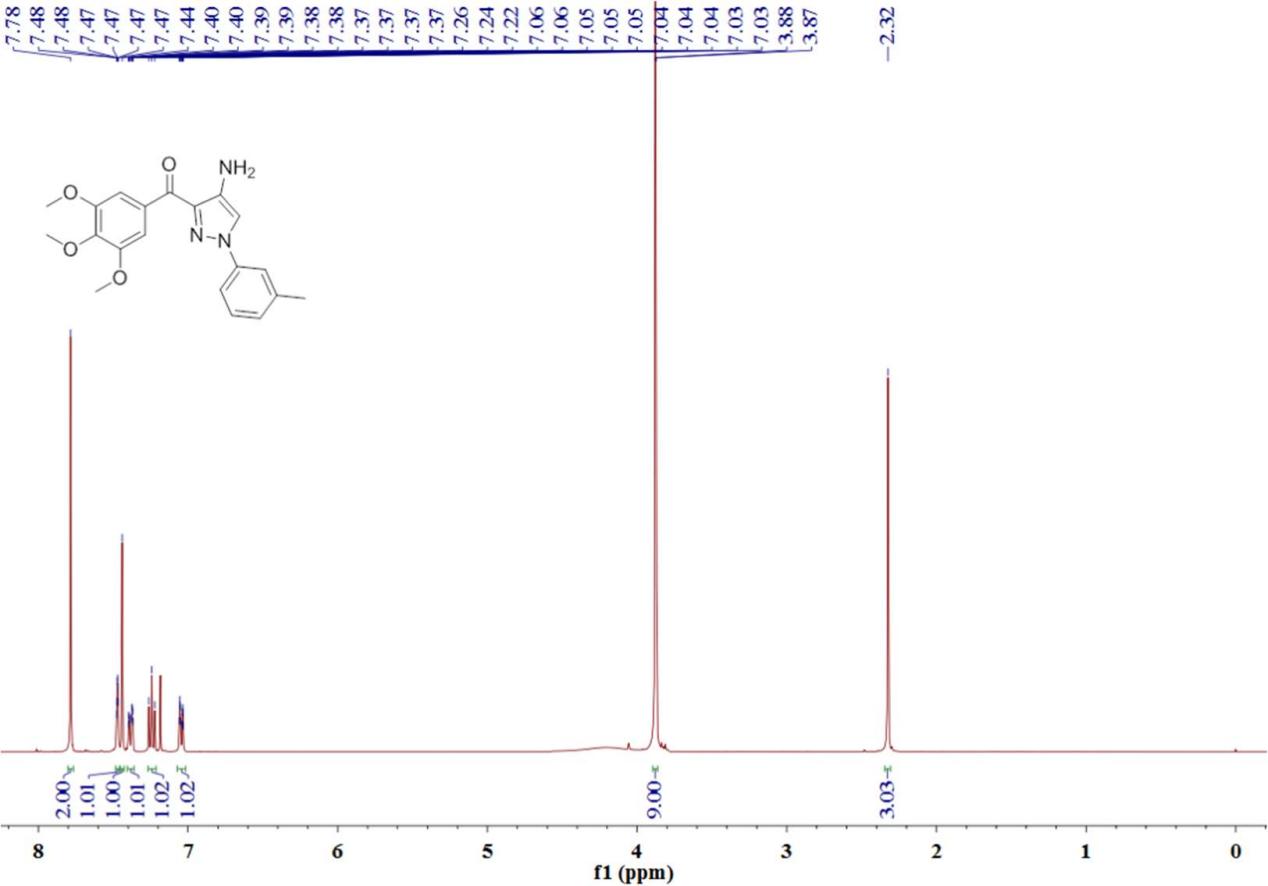


Fig. S3. ^1^H-NMR spectrum of **4b**.


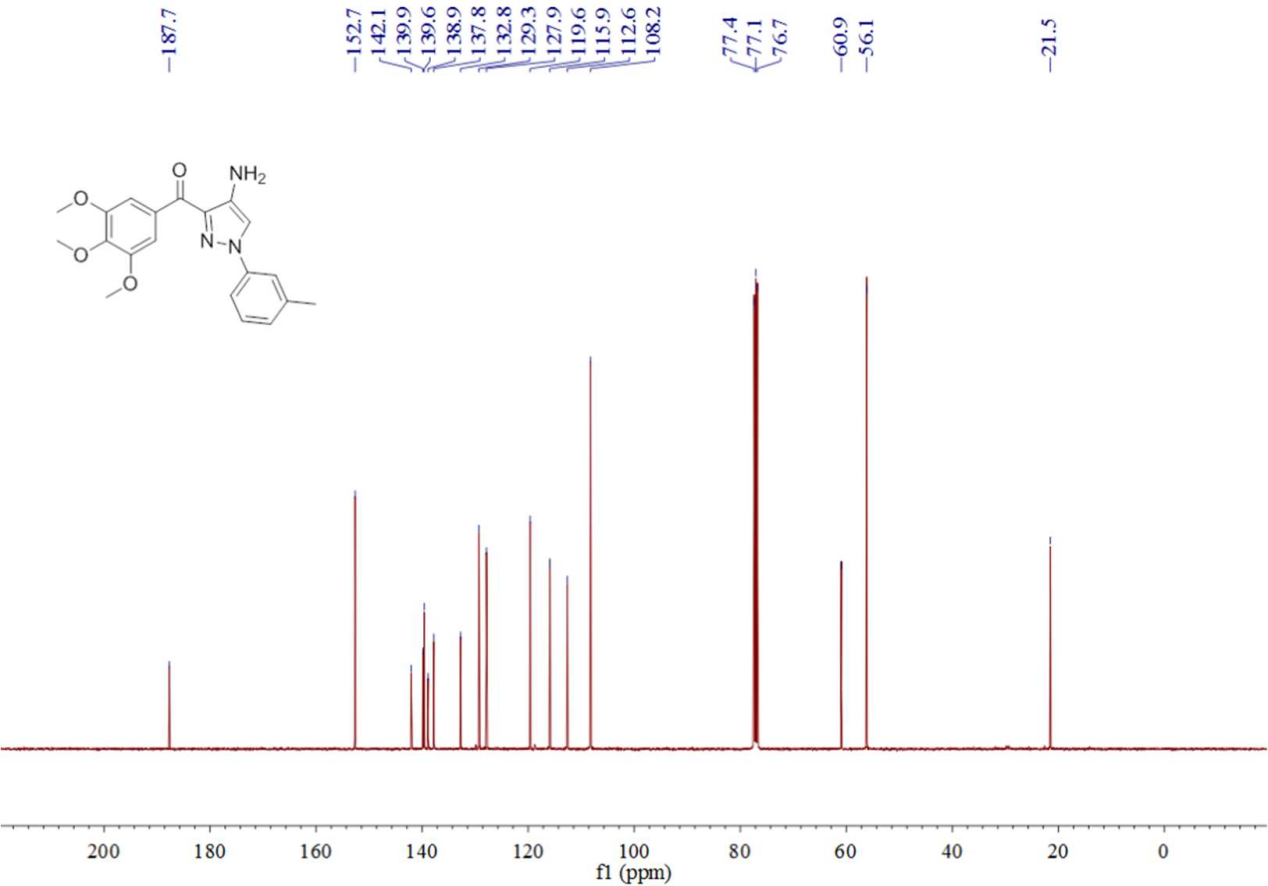


Fig. S4. ^13^C-NMR spectrum of **4b**.


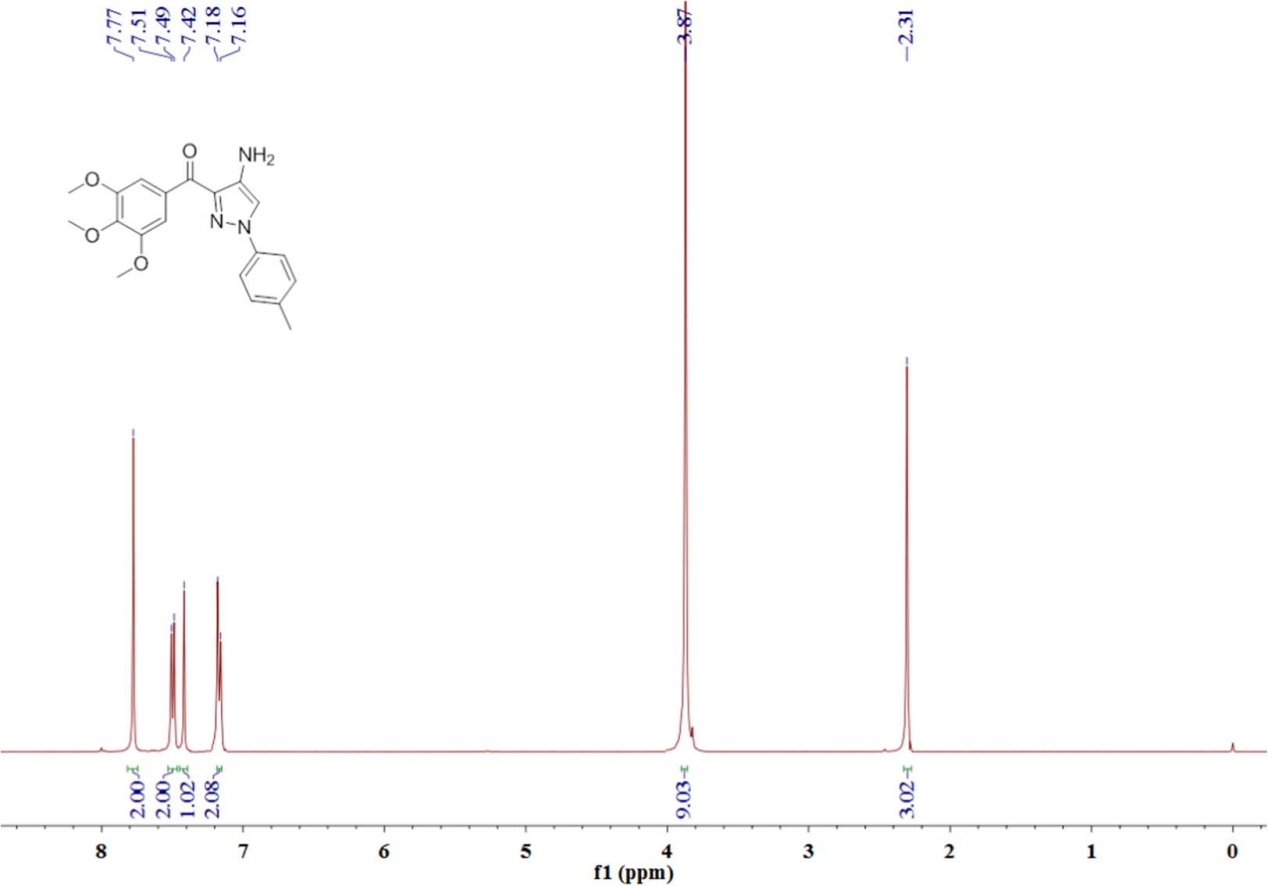


Fig. S5. ^1^H-NMR spectrum of **4c**.


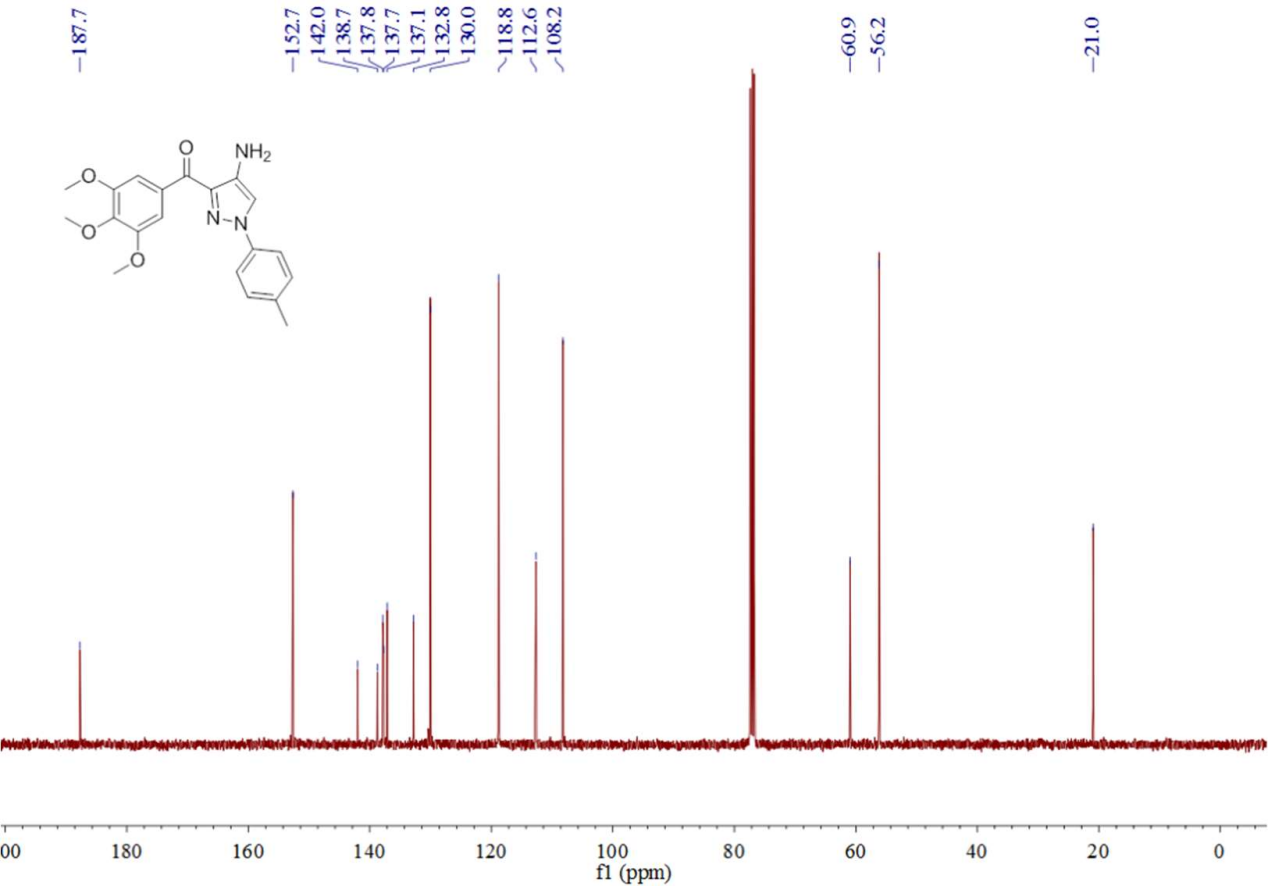


Fig. S6. ^13^C-NMR spectrum of **4c**.


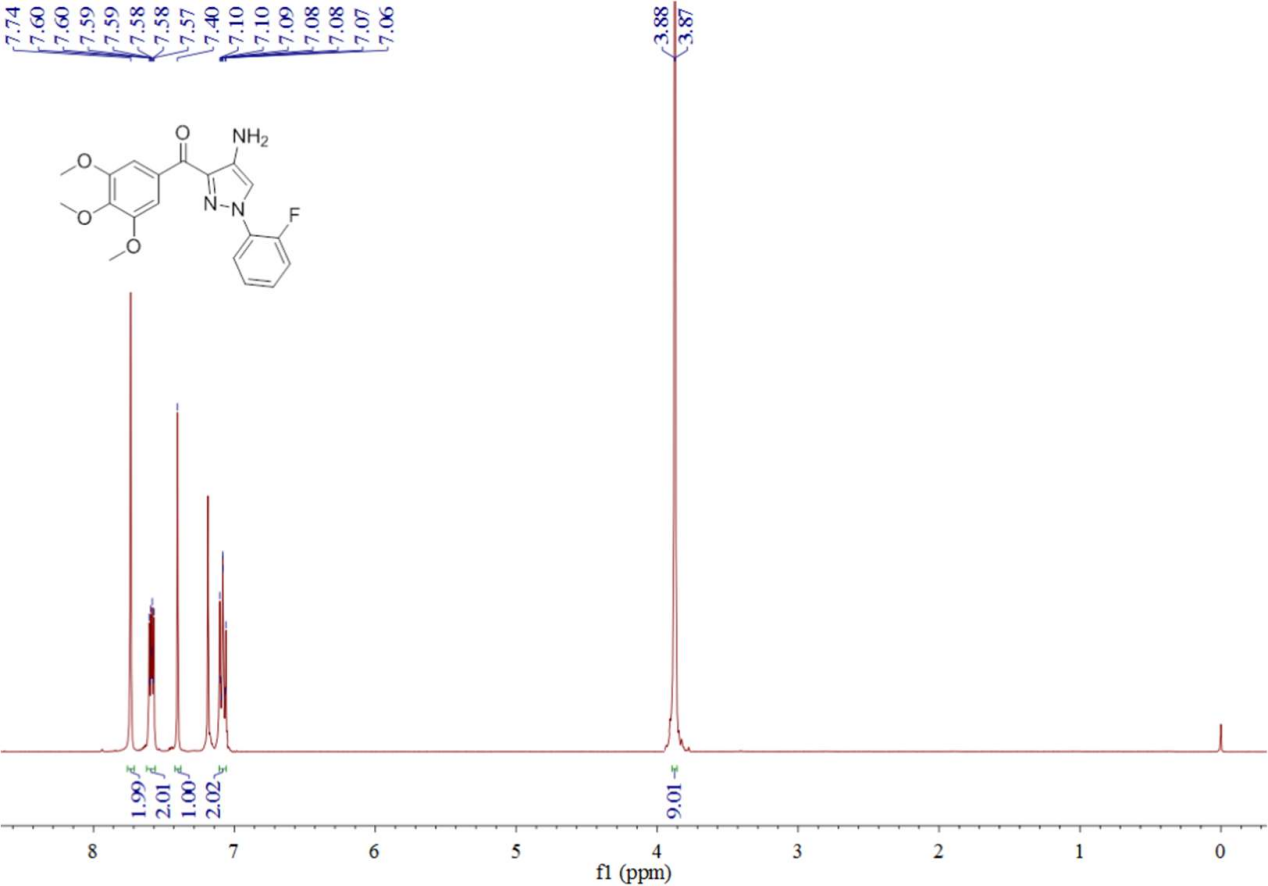


Fig. S7. ^1^H-NMR spectrum of **4d**.


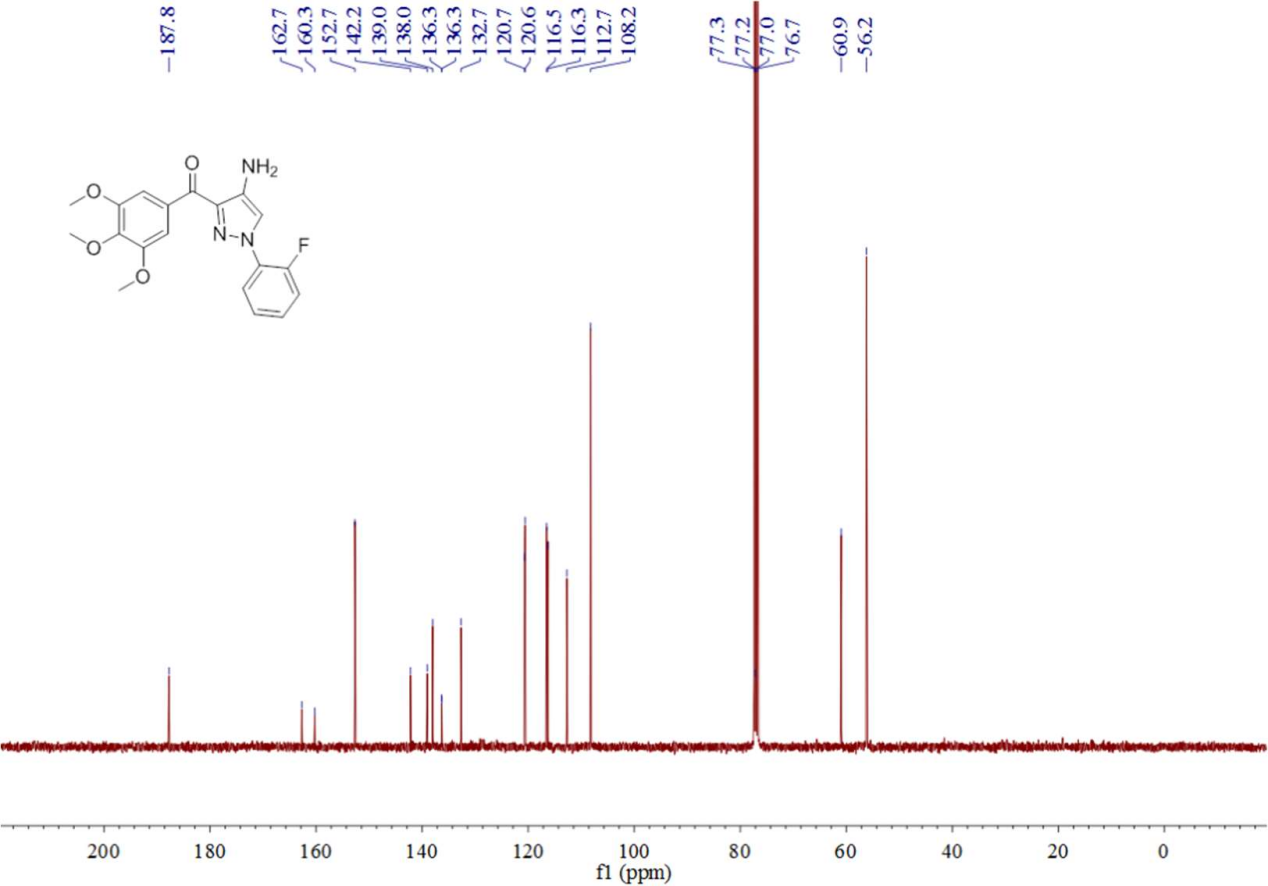


Fig. S8. ^13^C-NMR spectrum of **4d**.


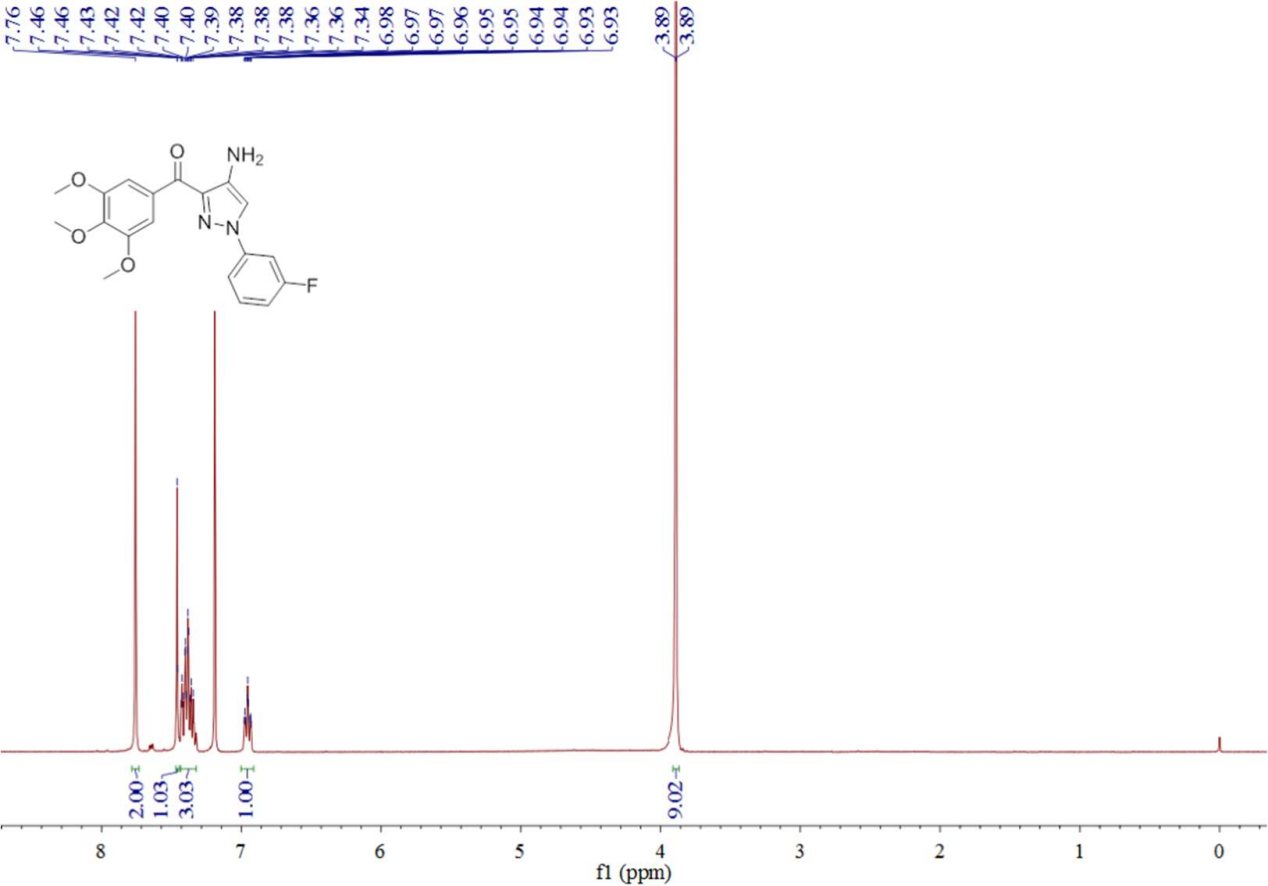


Fig. S9. ^1^H-NMR spectrum of **4e**.


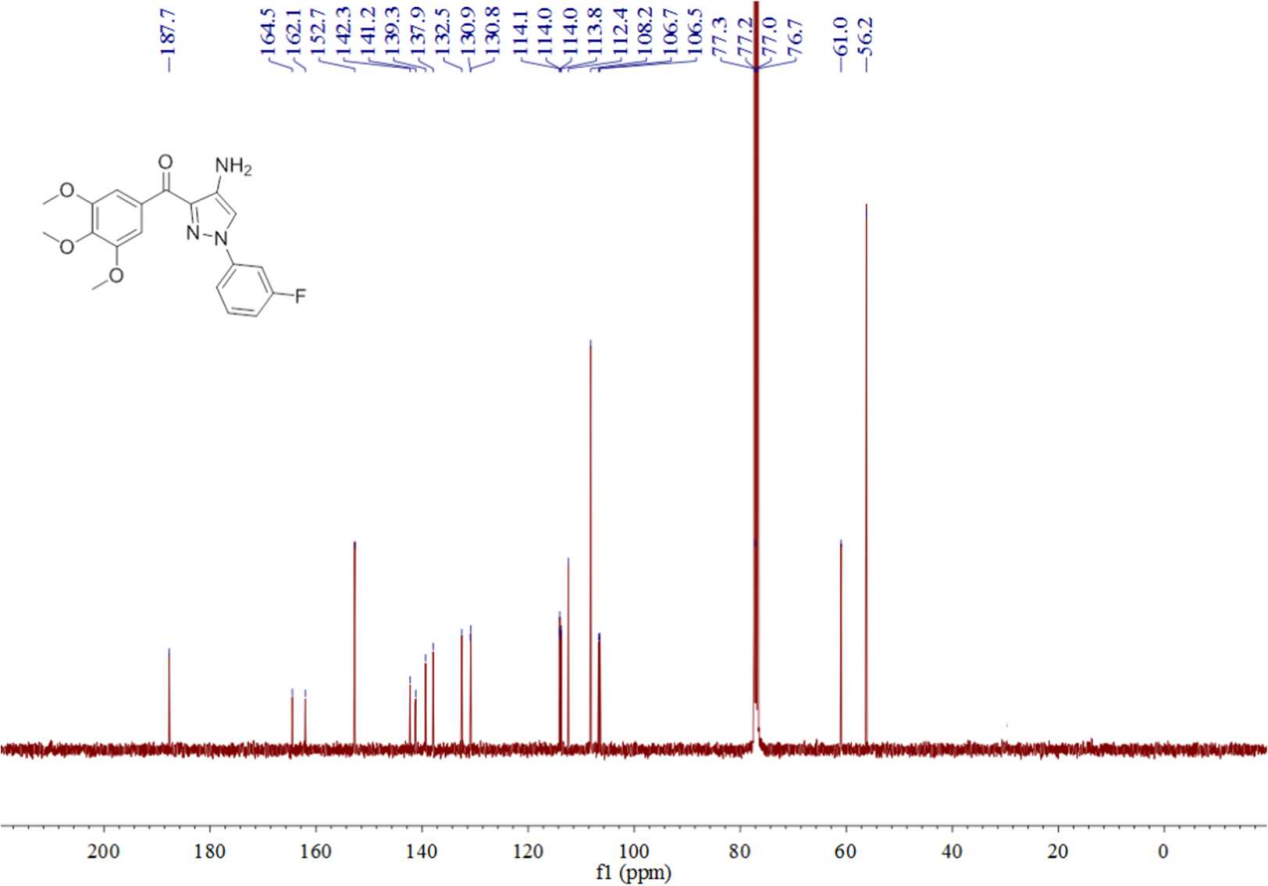


Fig. S10. ^13^C-NMR spectrum of **4e**.


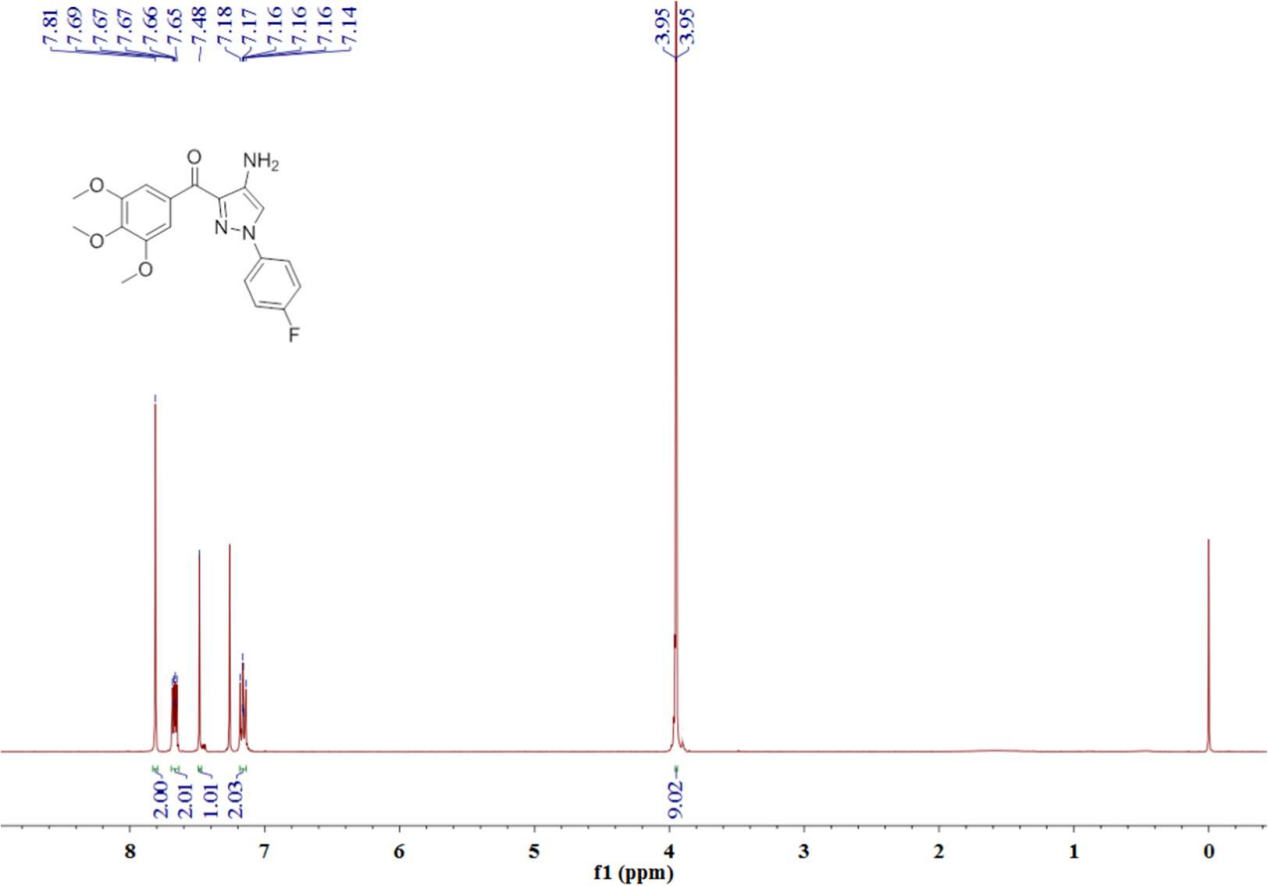


Fig. S11. ^1^H-NMR spectrum of **4f**.


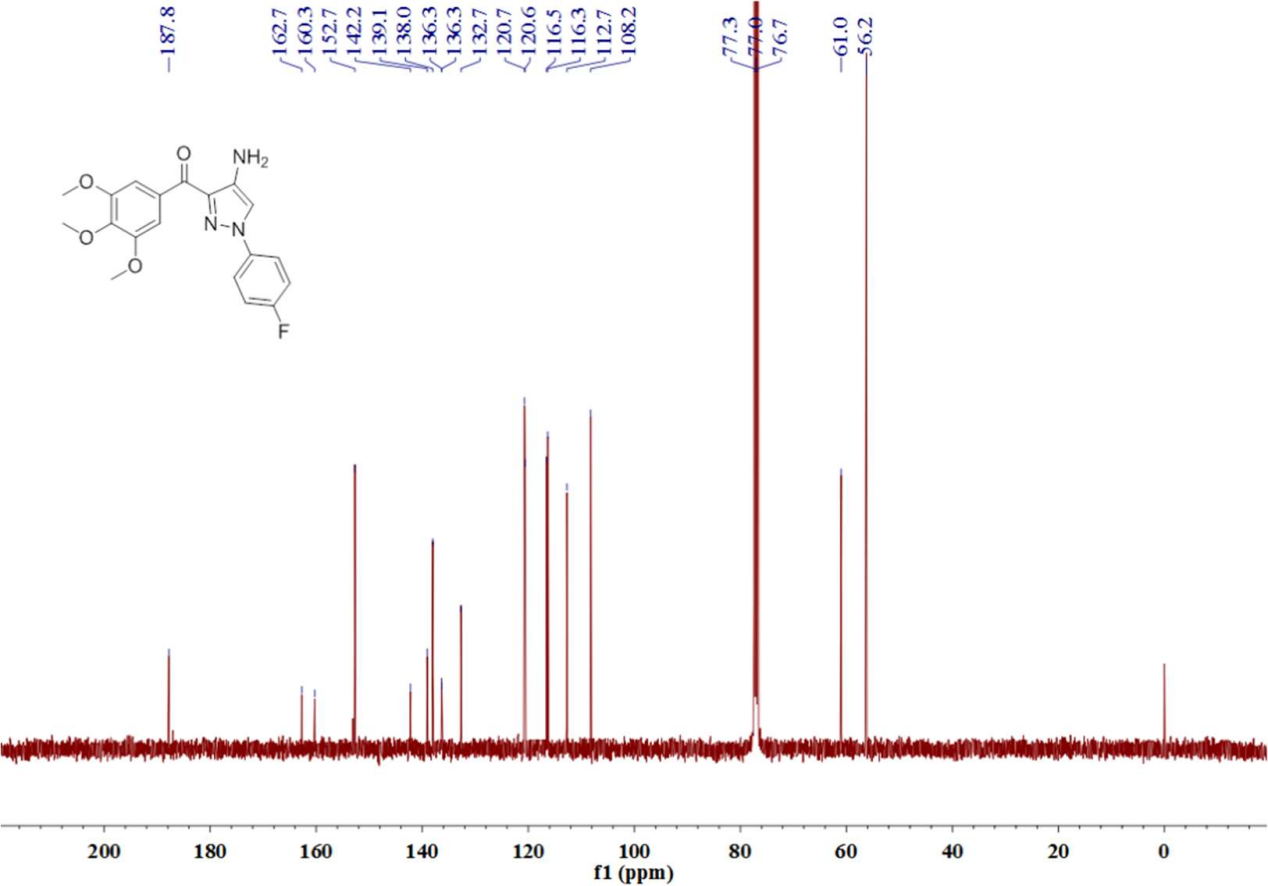


Fig. S12. ^13^C-NMR spectrum of **4f**.


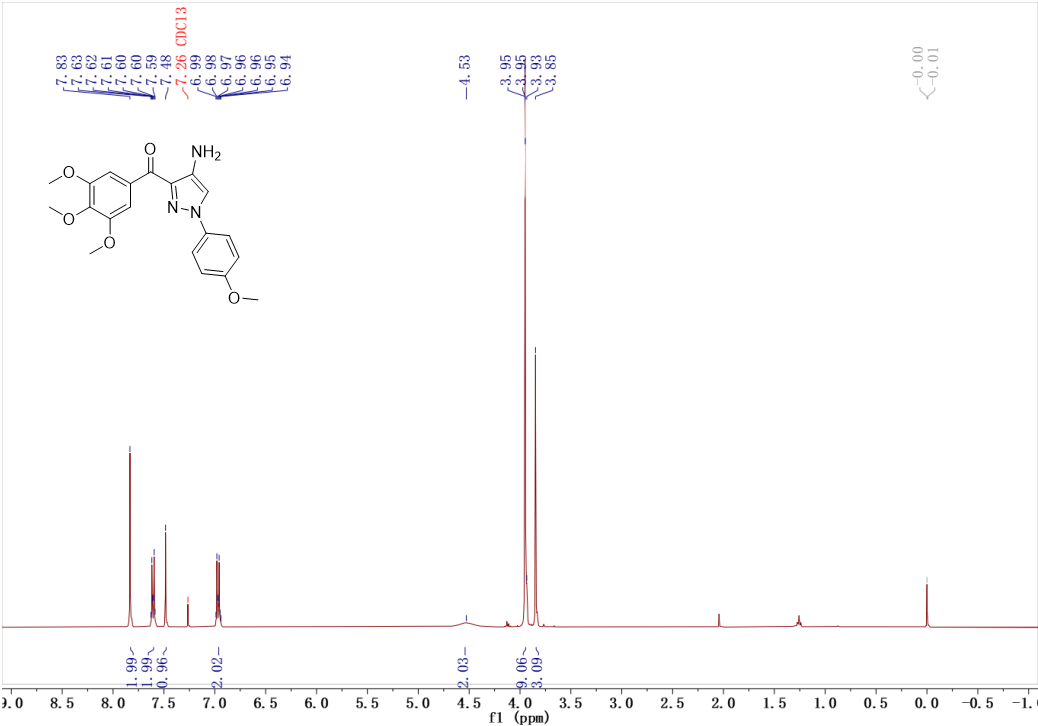


Fig. S13. ^1^H-NMR spectrum of **4g**.


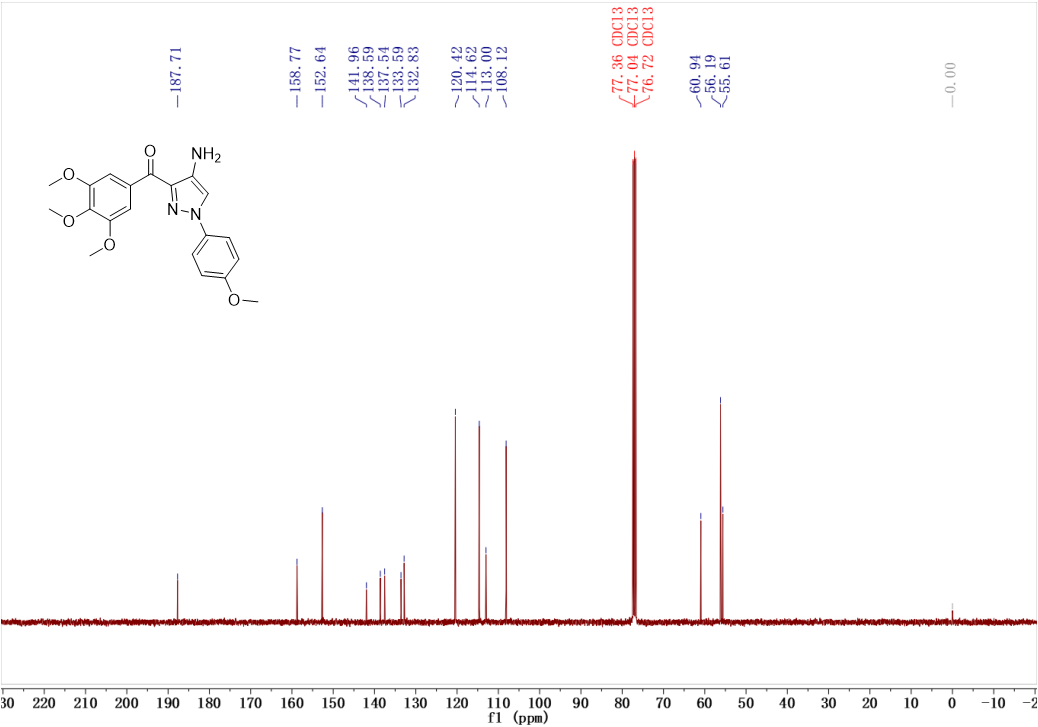


Fig. S14. ^13^C-NMR spectrum of **4g**.


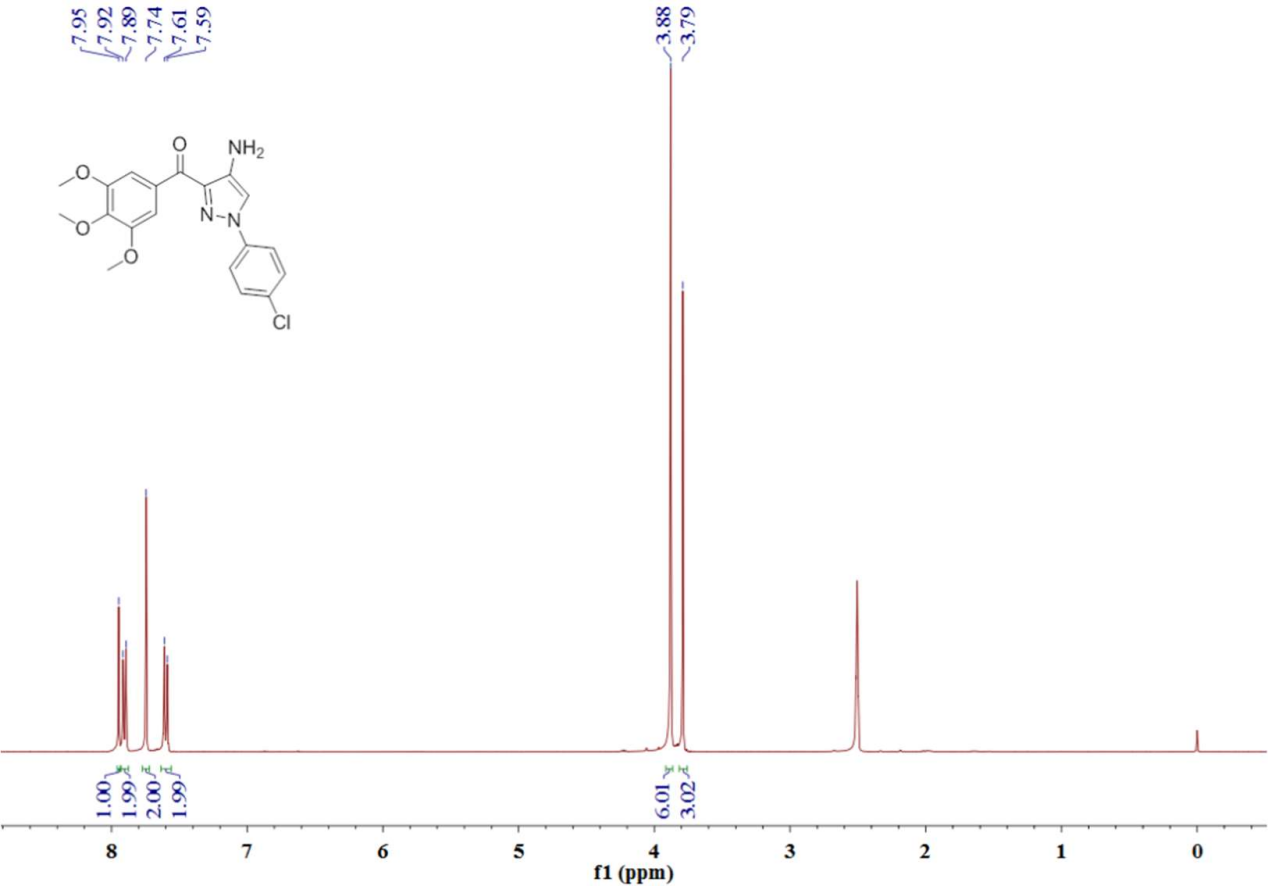


Fig. S15. ^1^H-NMR spectrum of **4h**.


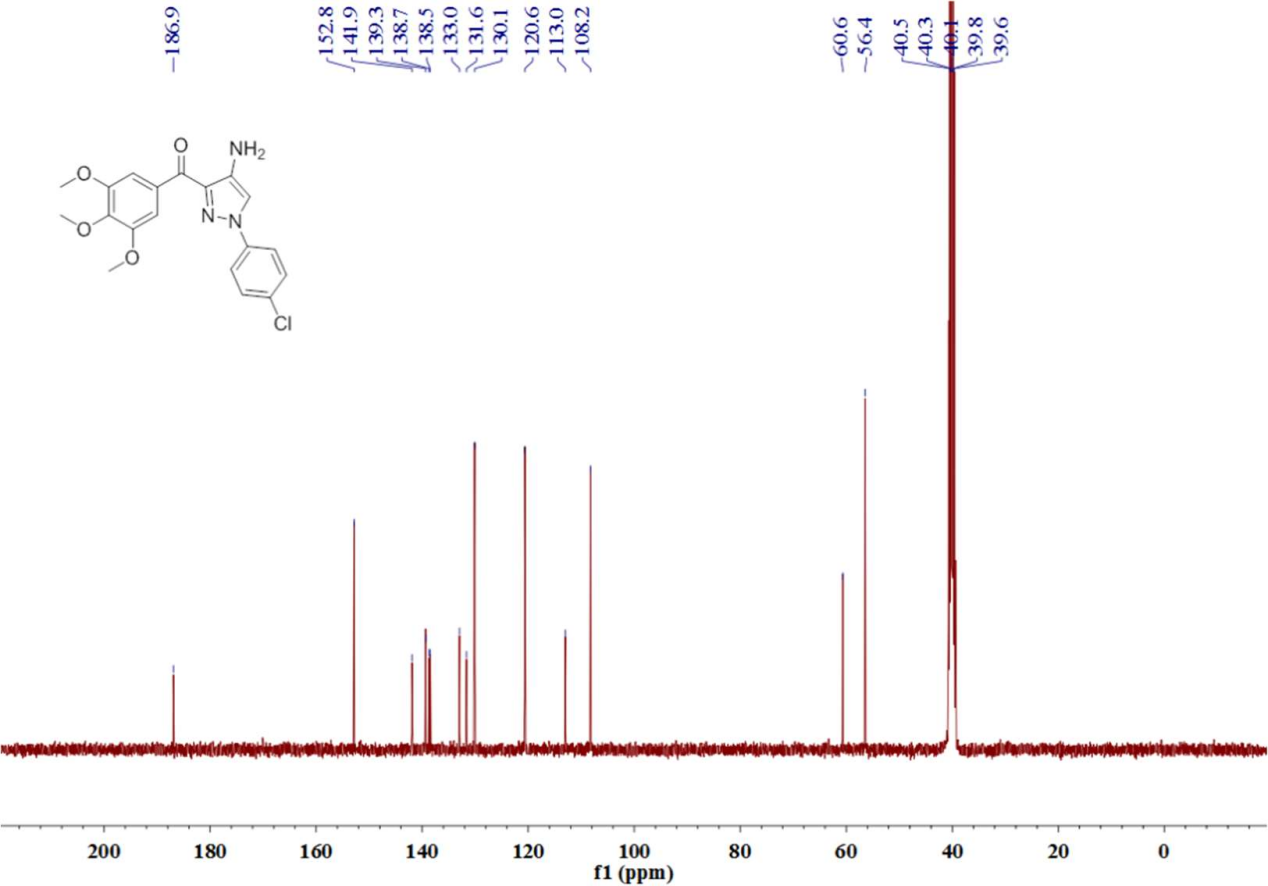


Fig. S16. ^13^C-NMR spectrum of **4h**.


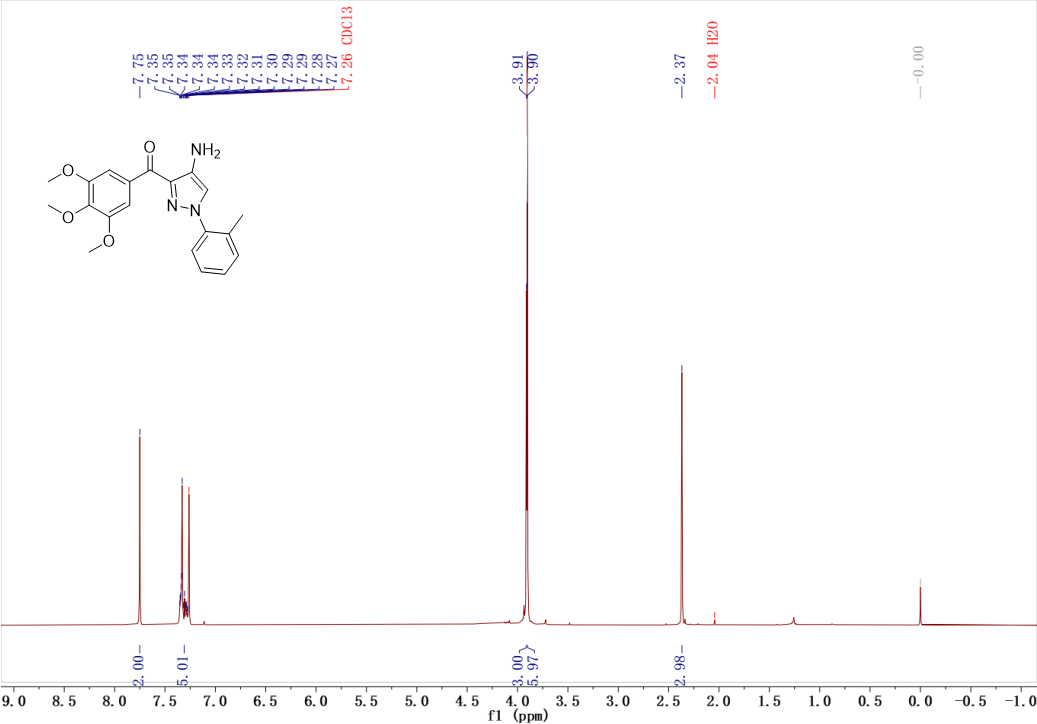


Fig. S17. ^1^H-NMR spectrum of **4i**.


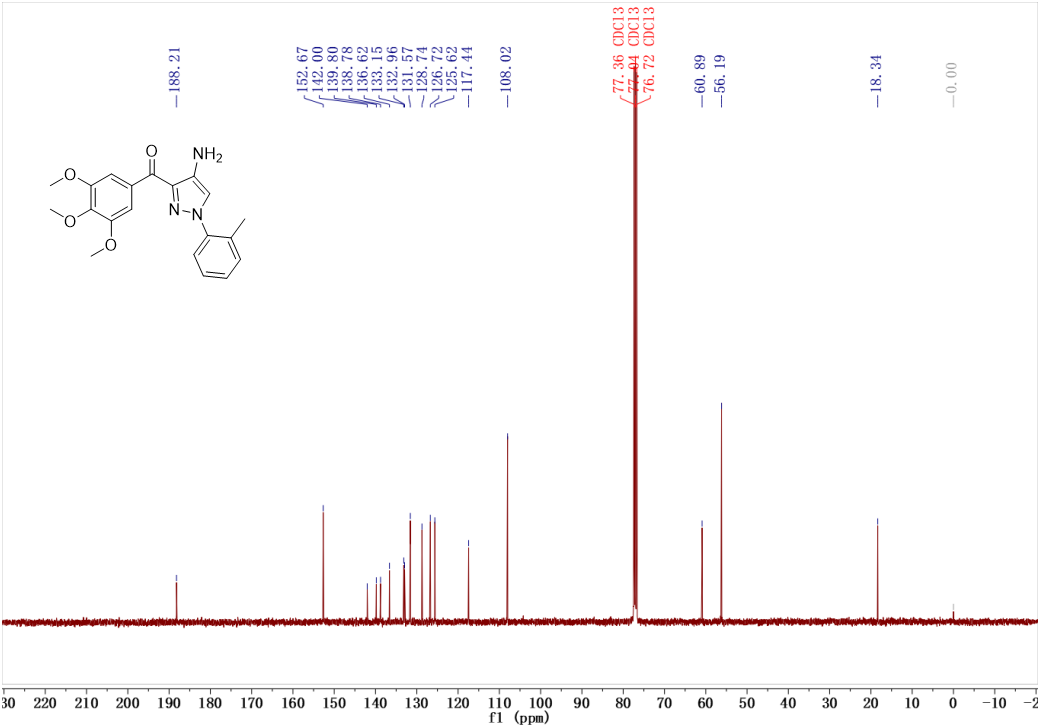


Fig. S18. ^13^C-NMR spectrum of **4i**.


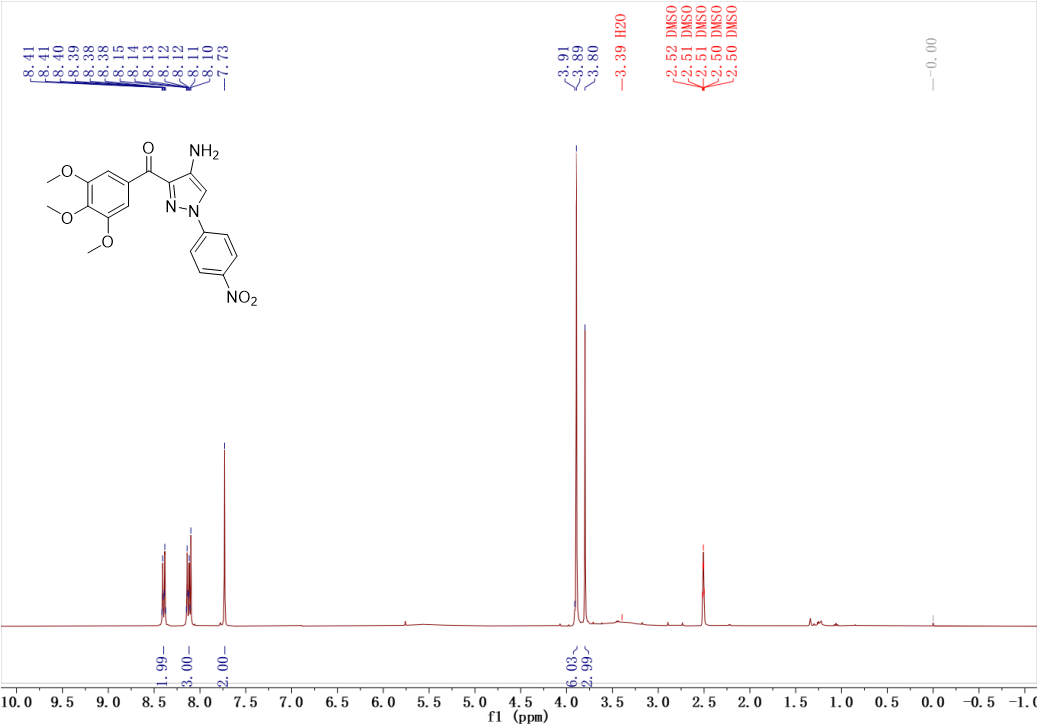


Fig. S19. ^1^H-NMR spectrum of **4j**.


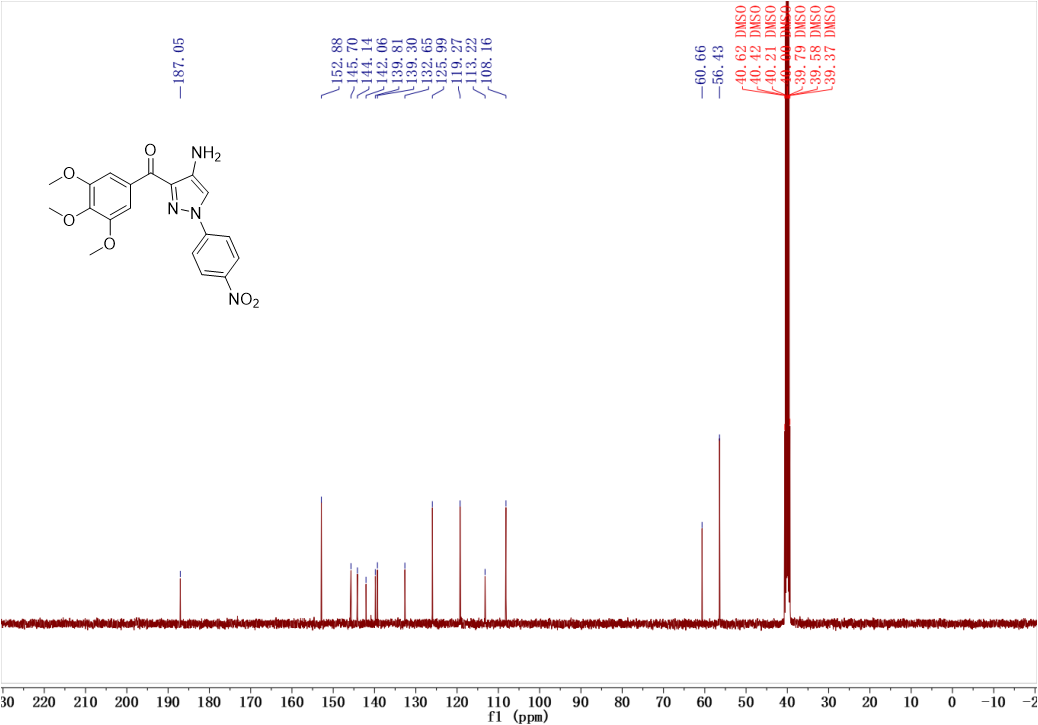


Fig. S20. ^13^C-NMR spectrum of **4j**.


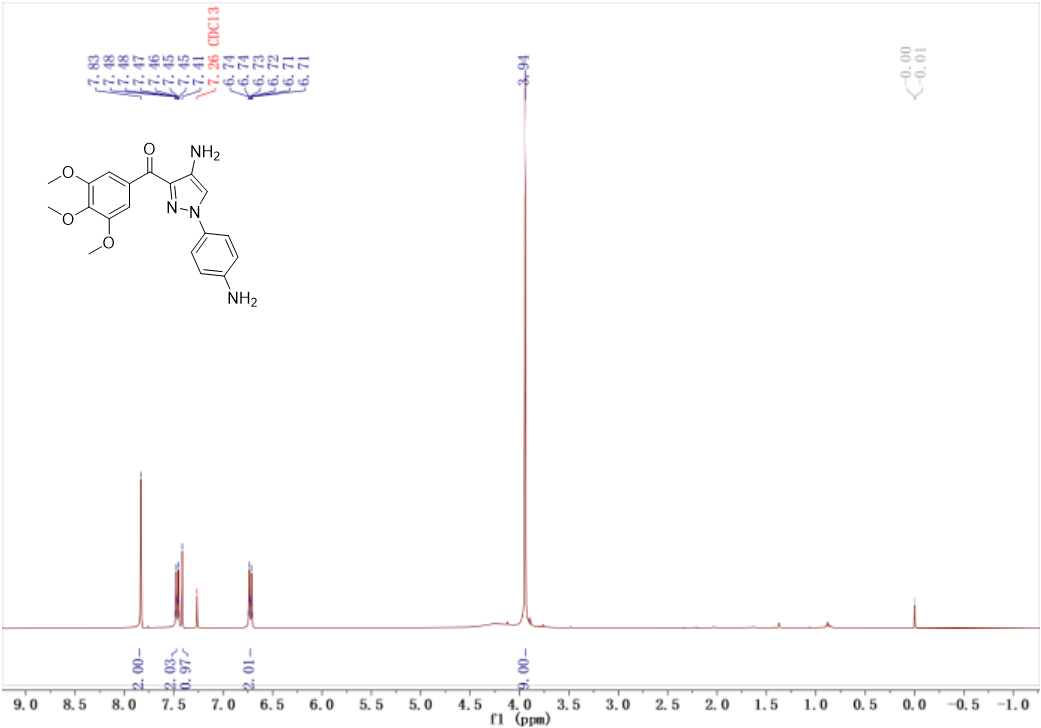


Fig. S21. ^1^H-NMR spectrum of **4k**.


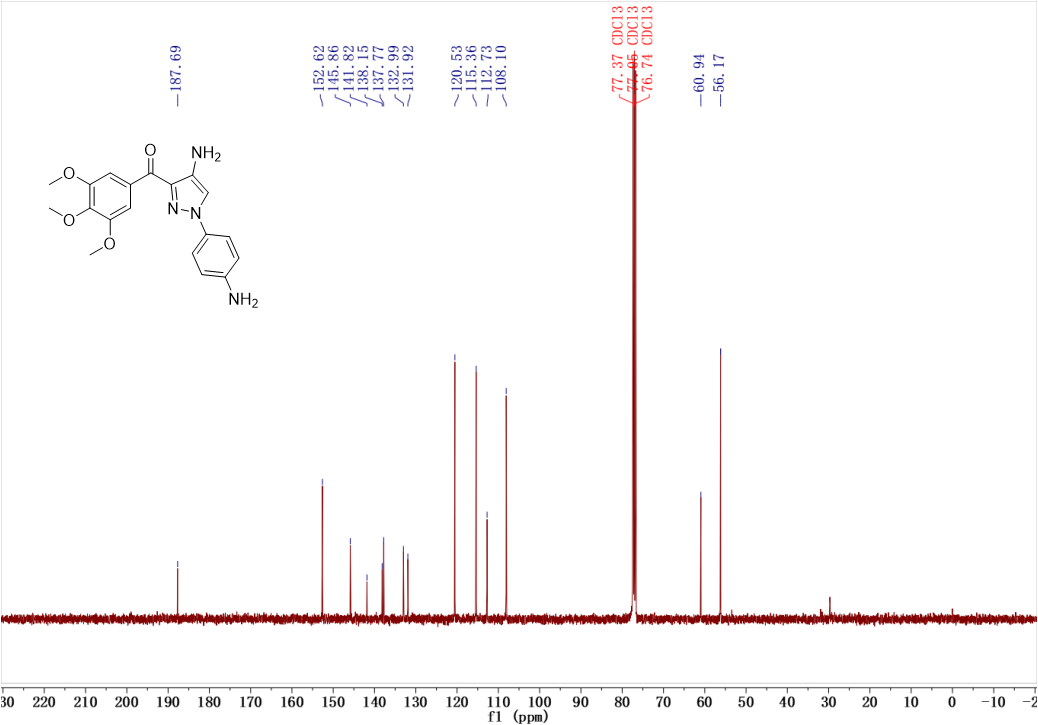


Fig. S22. ^13^C-NMR spectrum of **4k**.

**Contents: ^1^H NMR and ^13^C NMR spectra of 5a**−**5h**


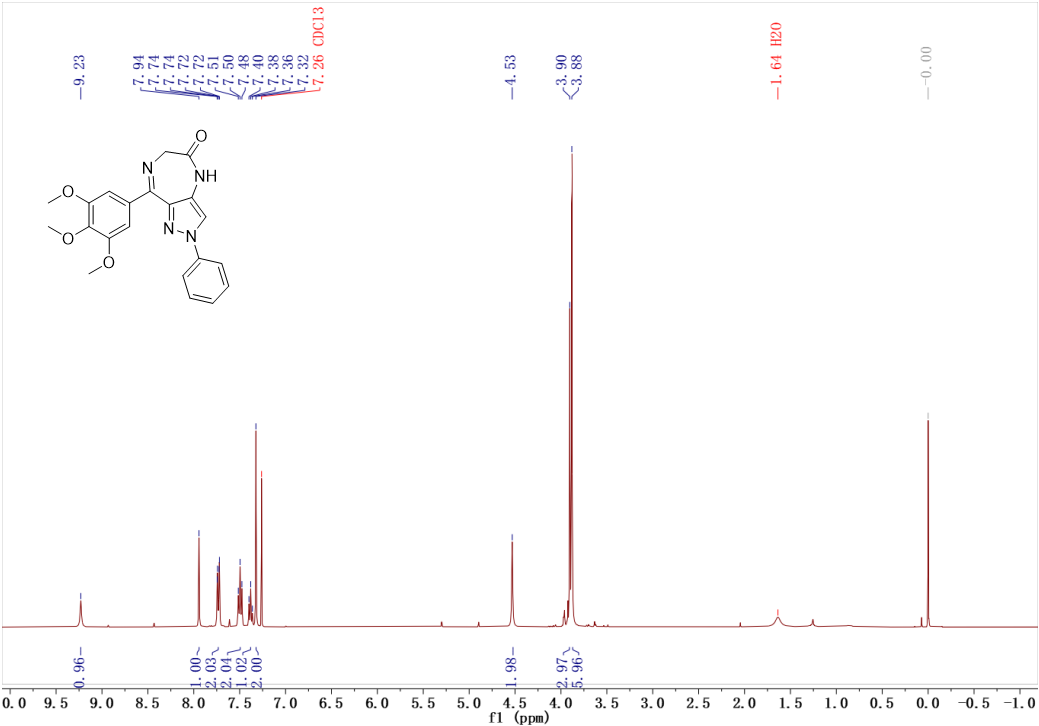


Fig. S23. ^1^H-NMR spectrum of **5a**.


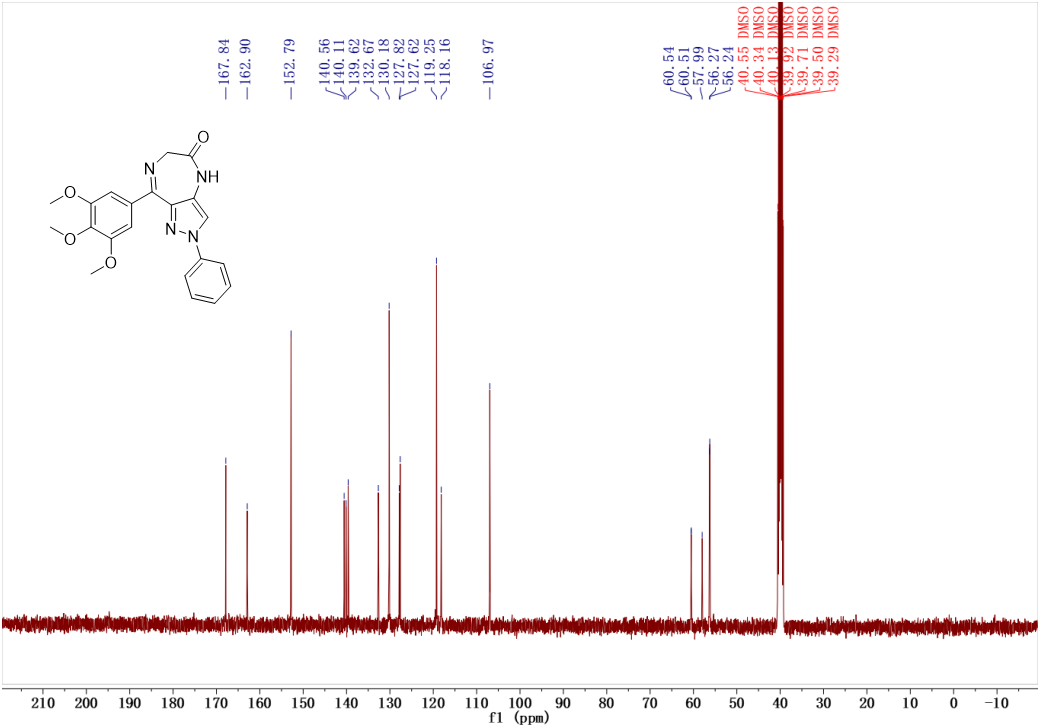


Fig. S24. ^13^C-NMR spectrum of **5a**.


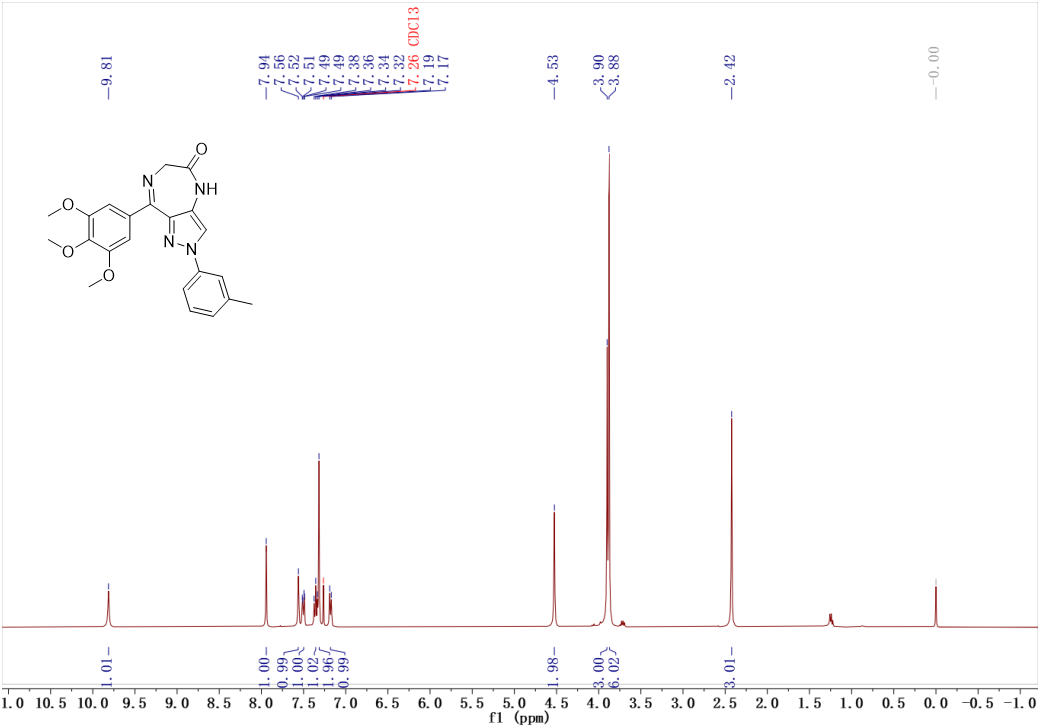


Fig. S25. ^1^H-NMR spectrum of **5b**.


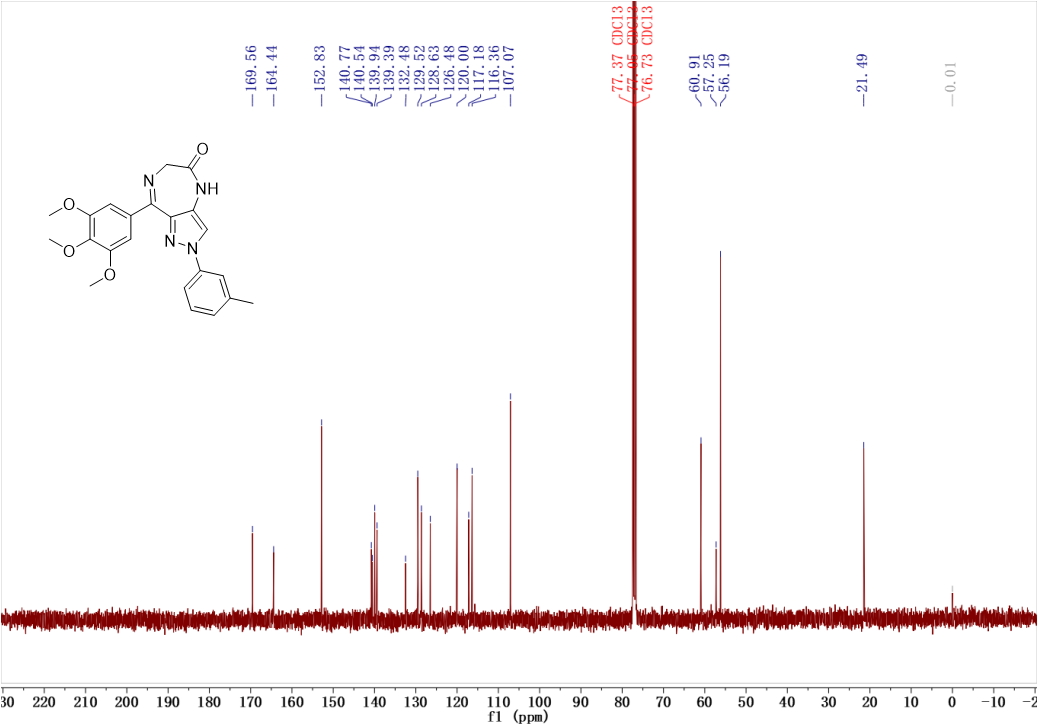


Fig. S26. ^13^C-NMR spectrum of **5b**.


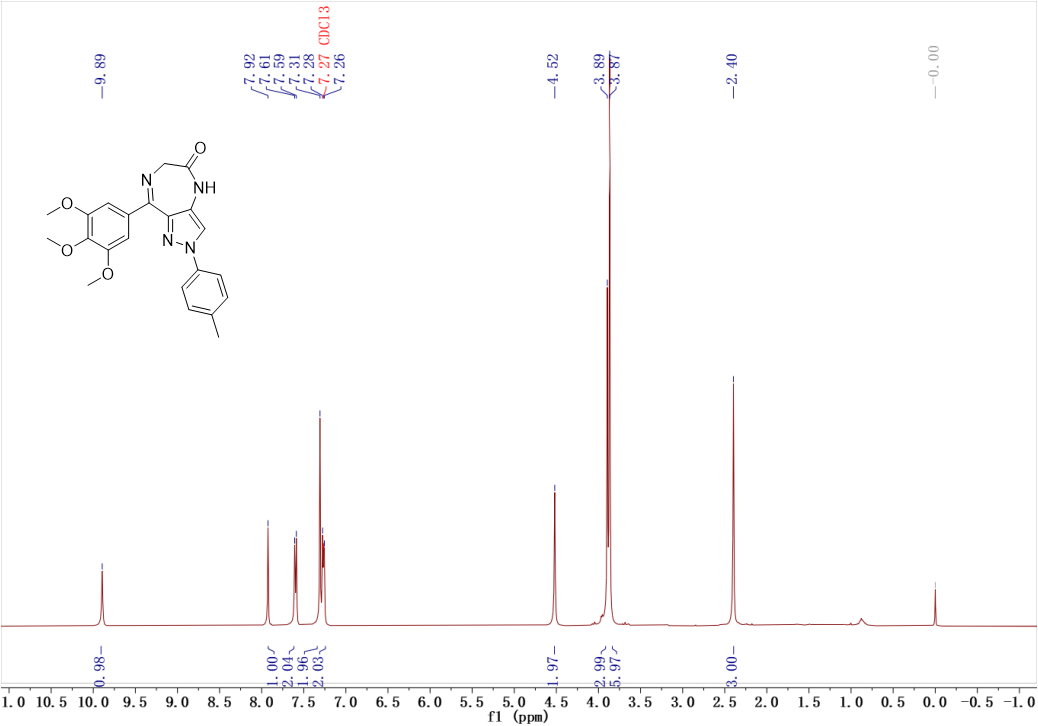


Fig. S27. ^1^H-NMR spectrum of **5c**.


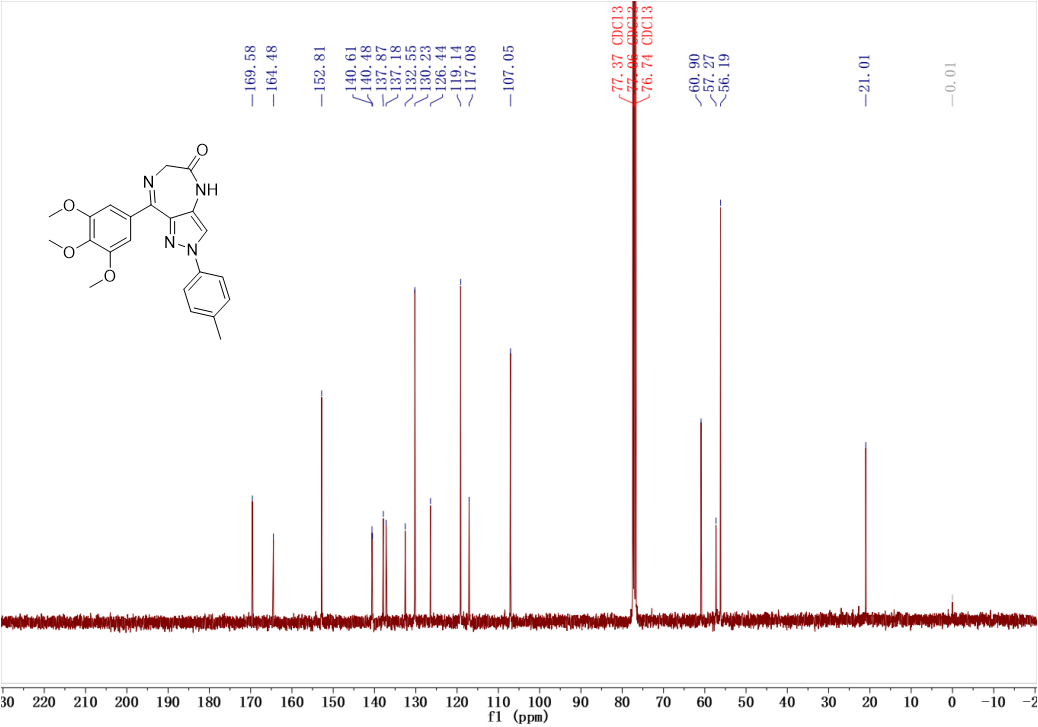


Fig. S28. ^13^C-NMR spectrum of **5c**.


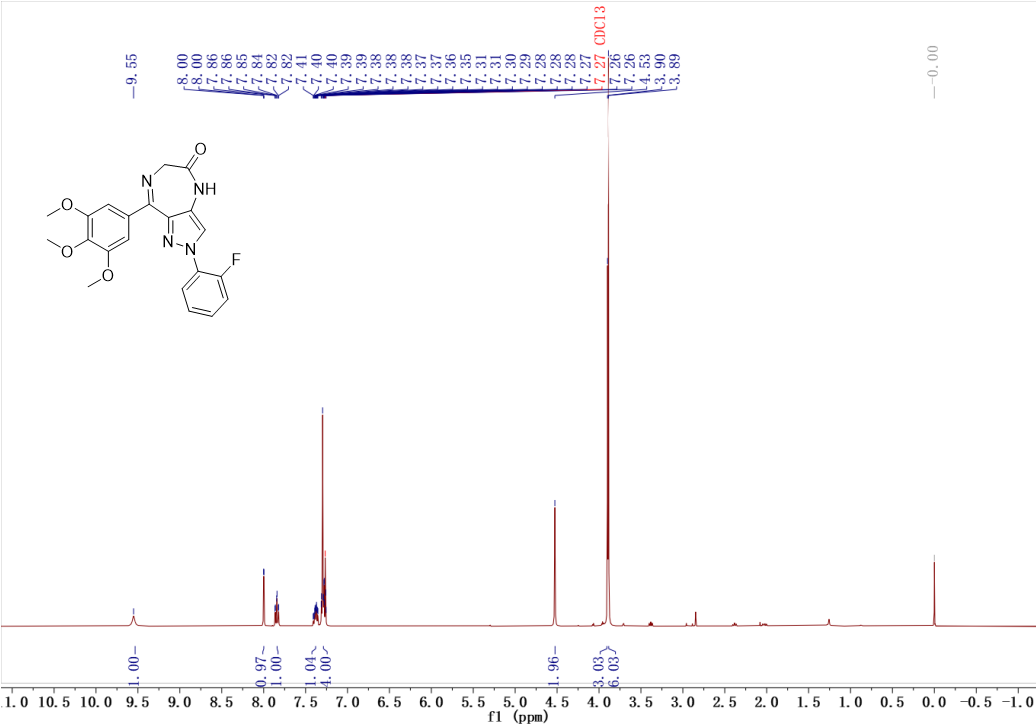


Fig. S29. ^1^H-NMR spectrum of **5d**.


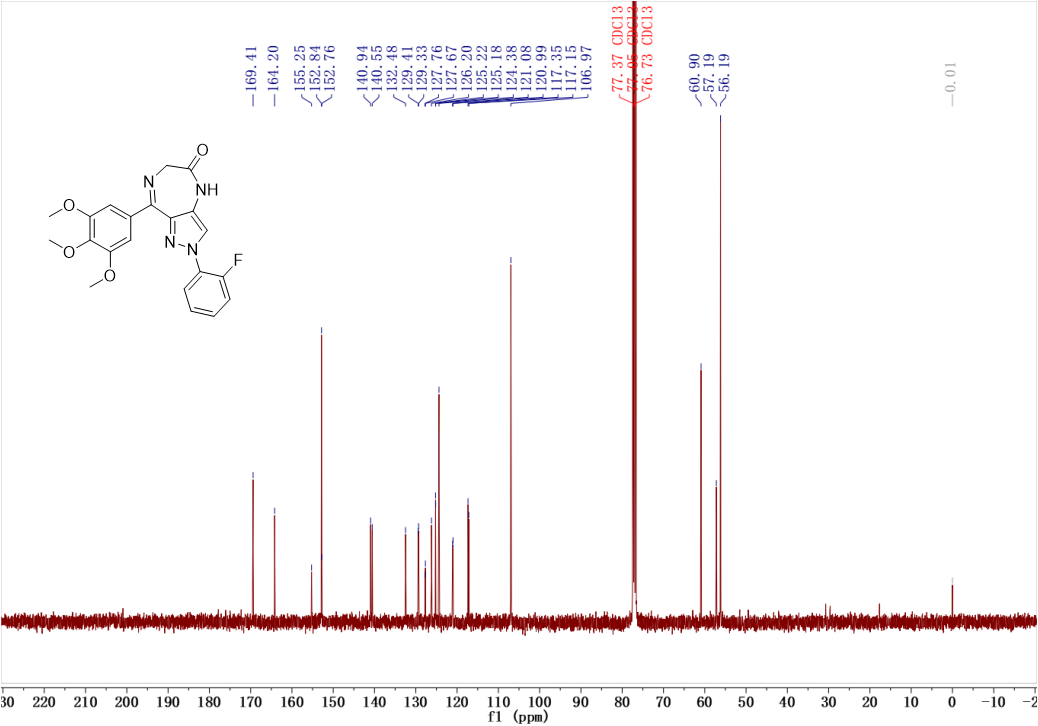


Fig. S30. ^13^C-NMR spectrum of **5d**.


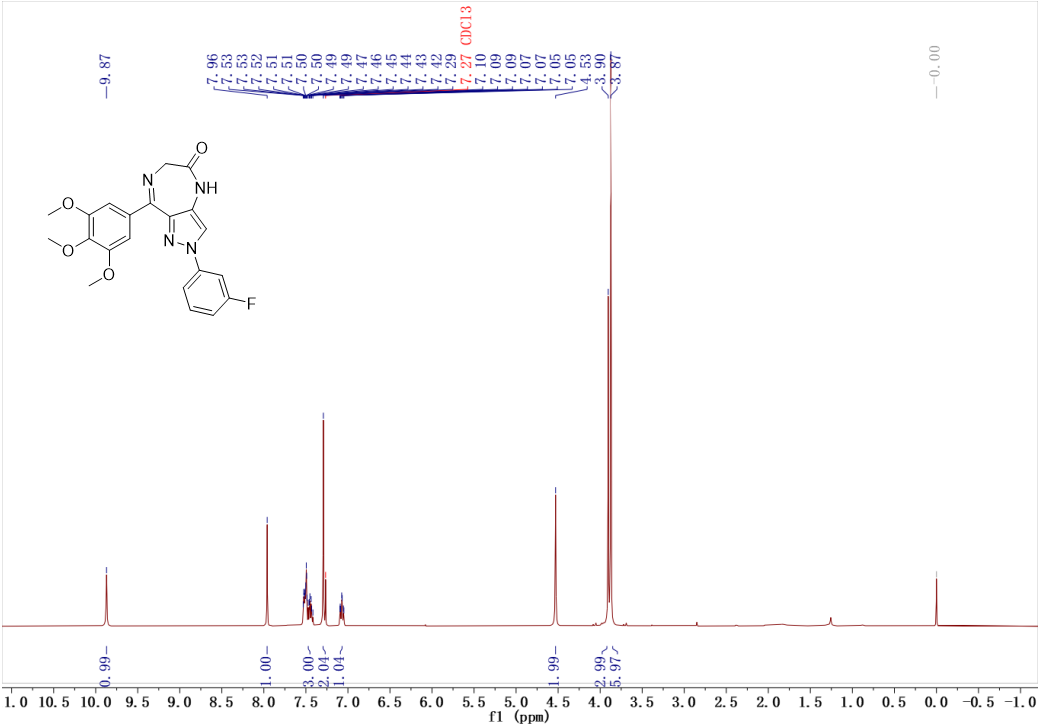


Fig. S31. ^1^H-NMR spectrum of **5e**.


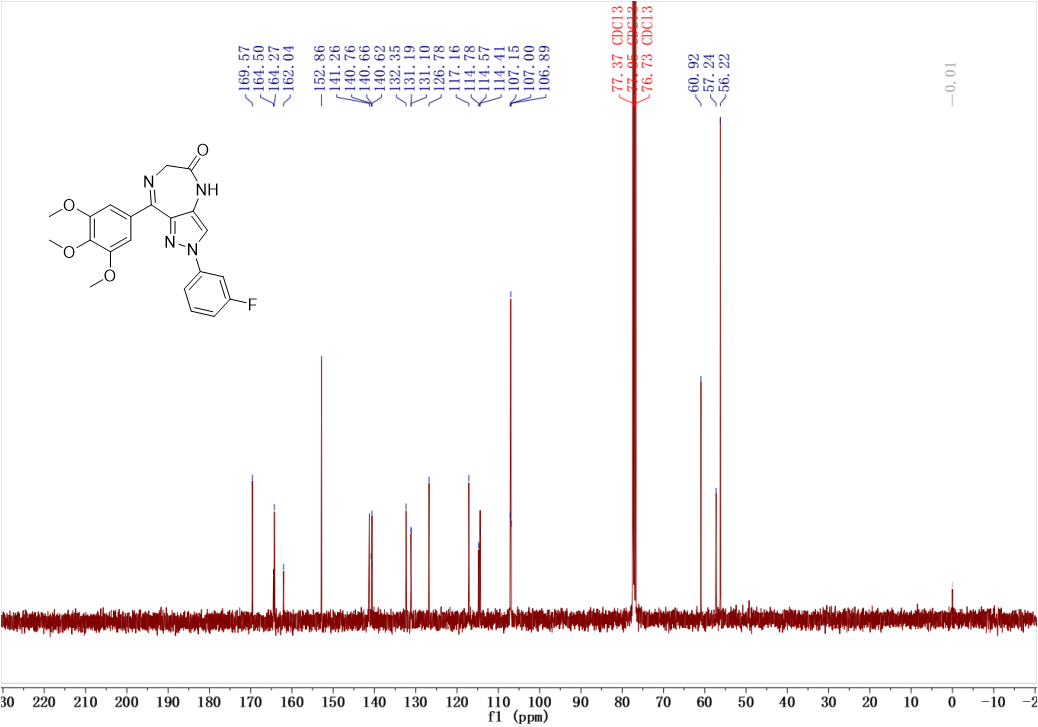


Fig. S32. ^13^C-NMR spectrum of **5e**.


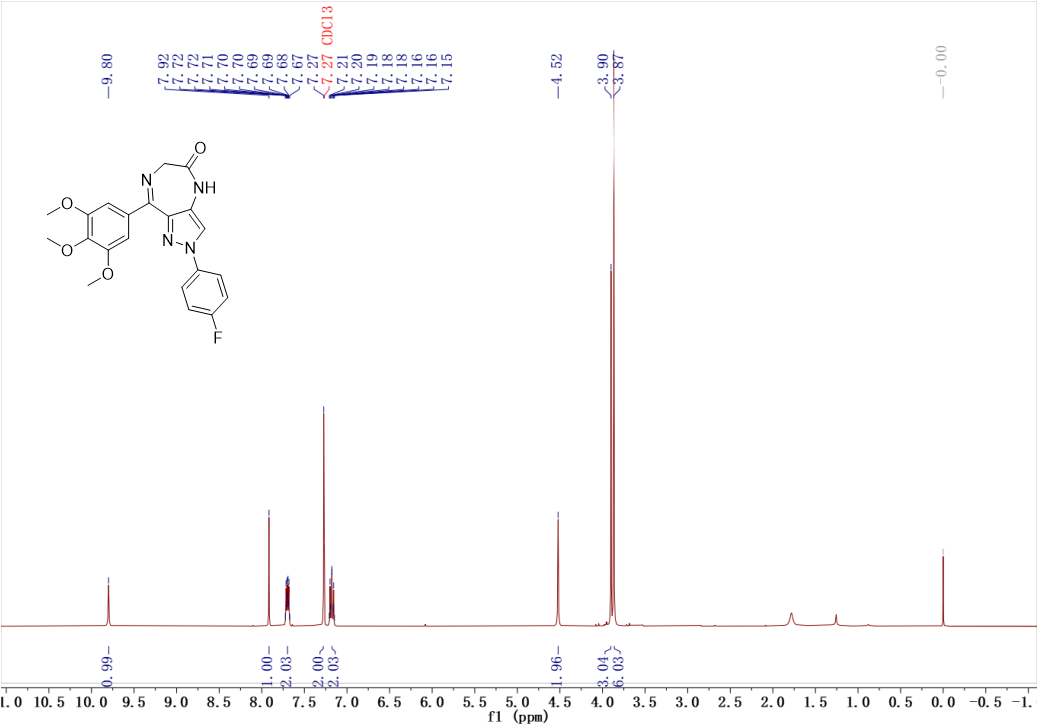


Fig. S33. ^1^H-NMR spectrum of **5f**.


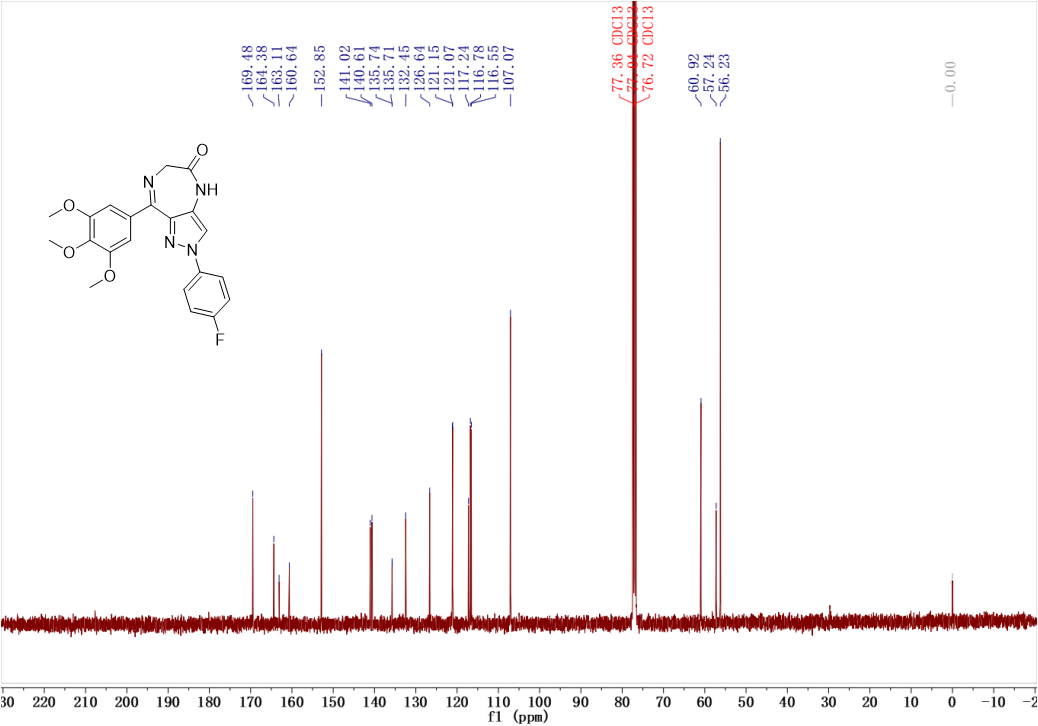


Fig. S34. ^13^C-NMR spectrum of **5f**.


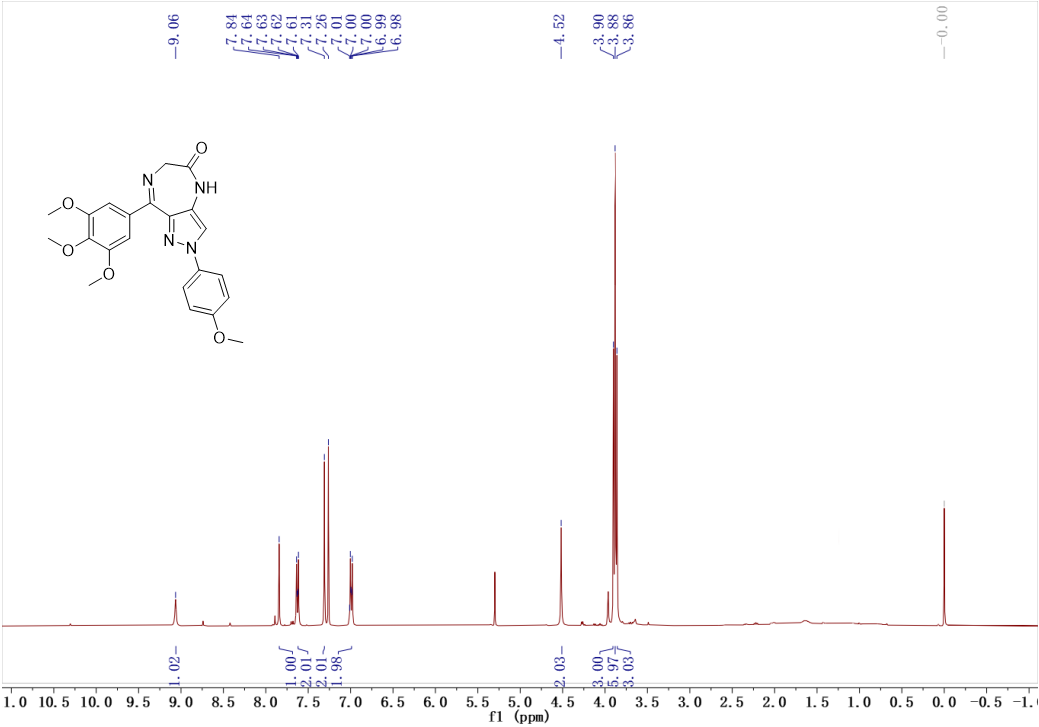


Fig. S35. ^1^H-NMR spectrum of **5g**.


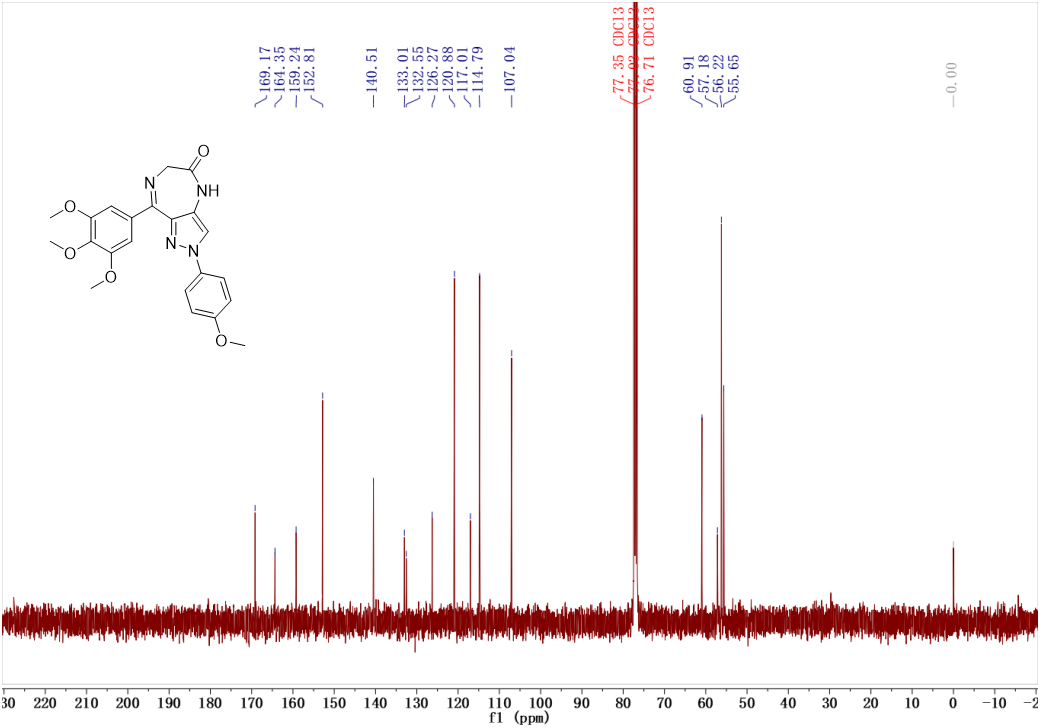


Fig. S36. ^13^C-NMR spectrum of **5g**.


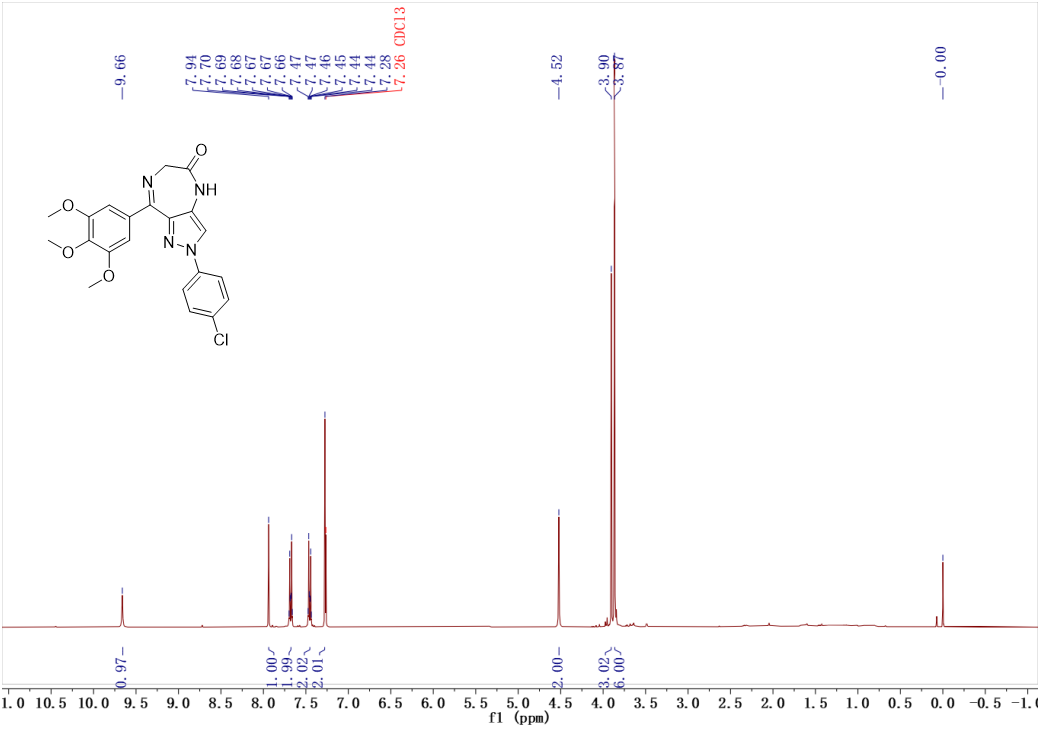


Fig. S37. ^1^H-NMR spectrum of **5h**.


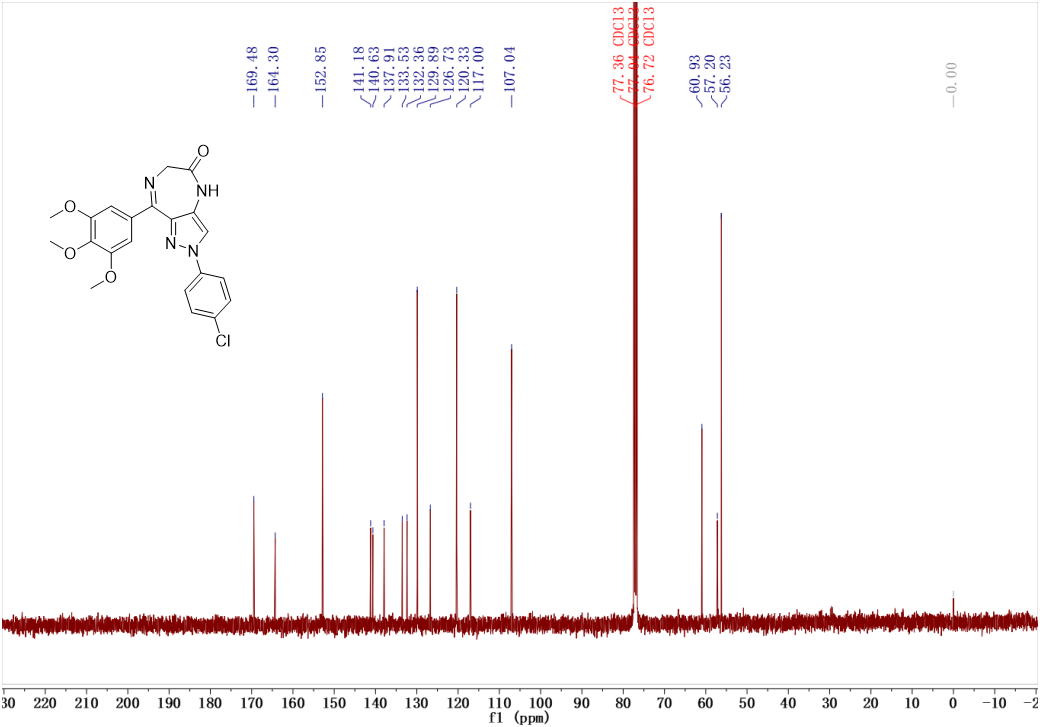


Fig. S38. ^13^C-NMR spectrum of **5h**.

**Contents: ^1^H NMR and ^13^C NMR spectra of 6a**−**6h**


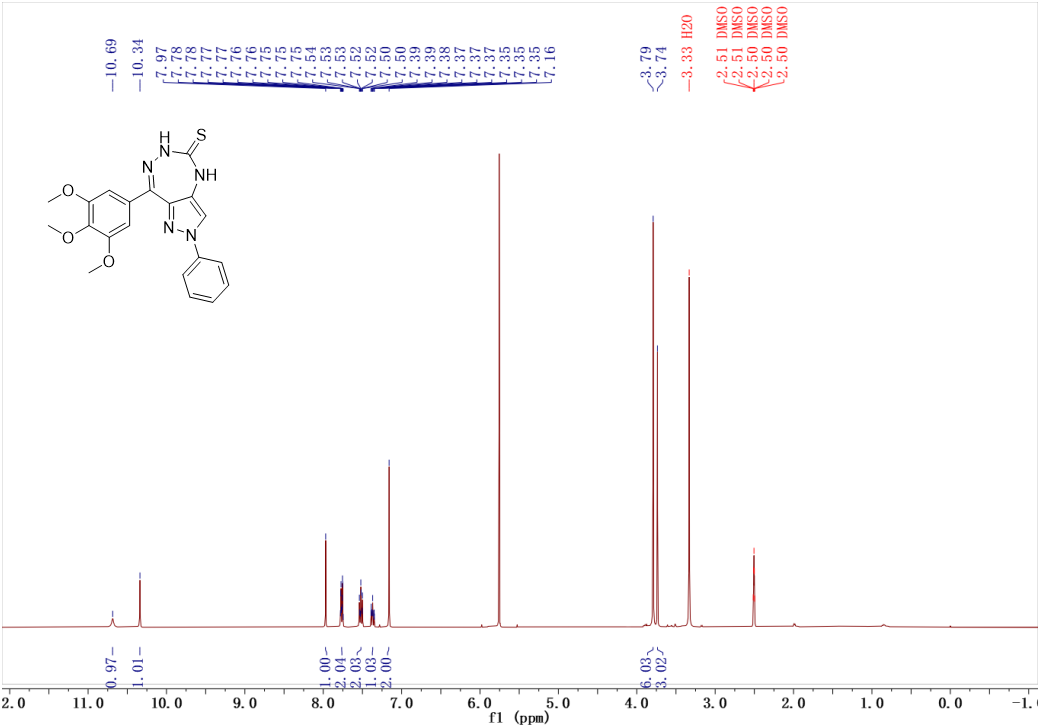


Fig. S39. ^1^H-NMR spectrum of **6a**.


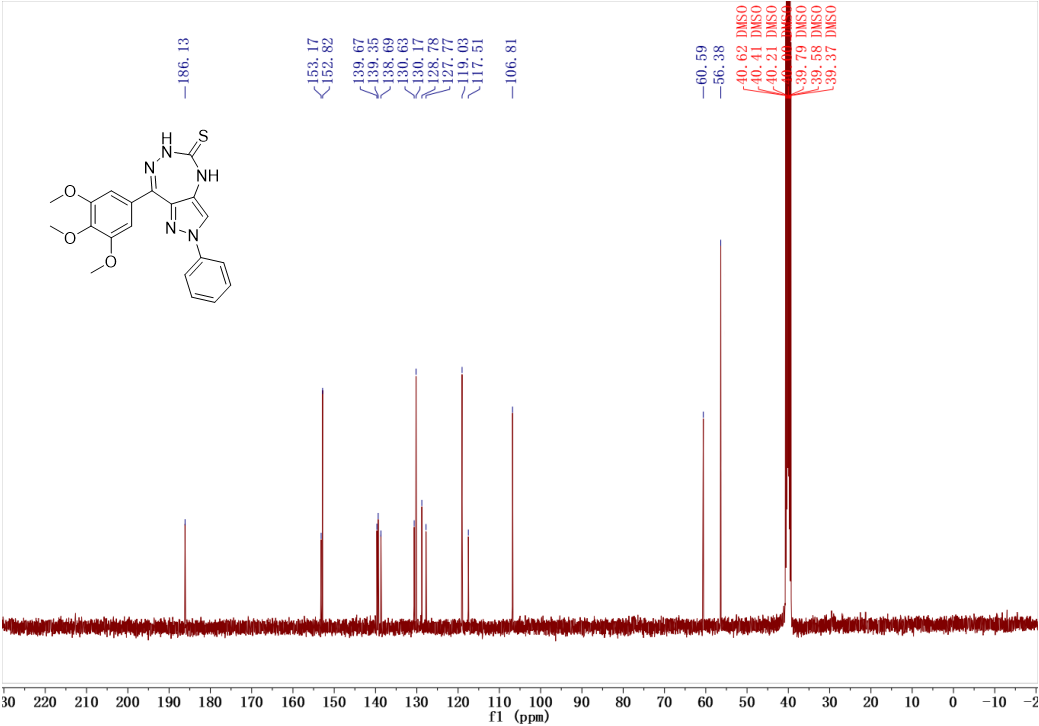


Fig. S40. ^13^C-NMR spectrum of **6a**.


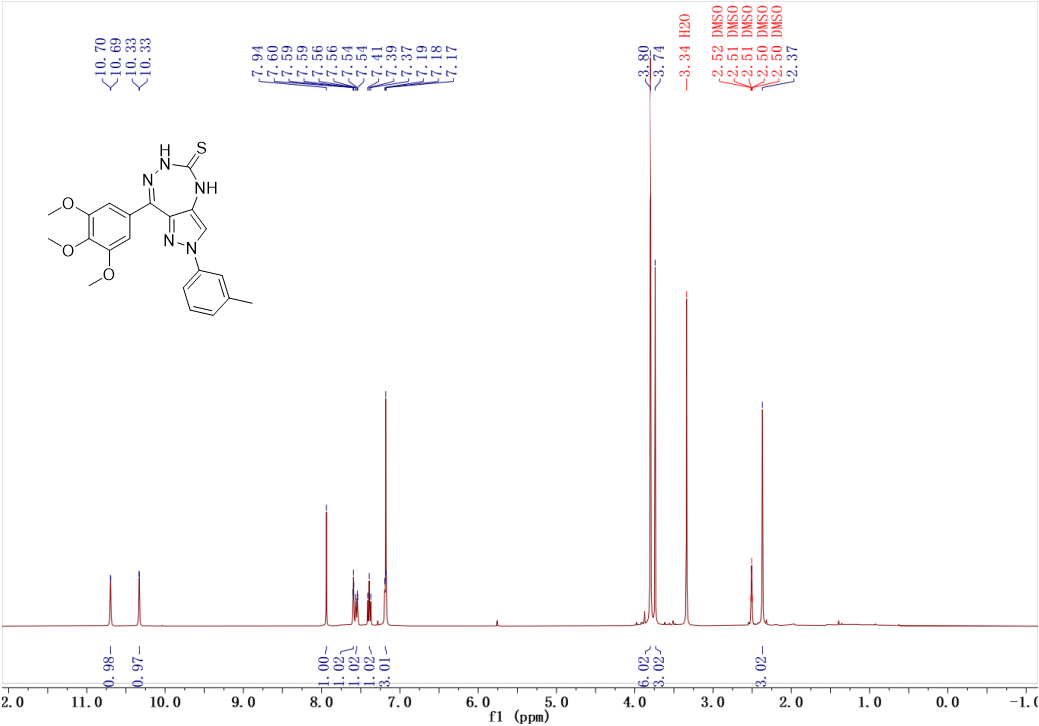


Fig. S41. ^1^H-NMR spectrum of **6b**.


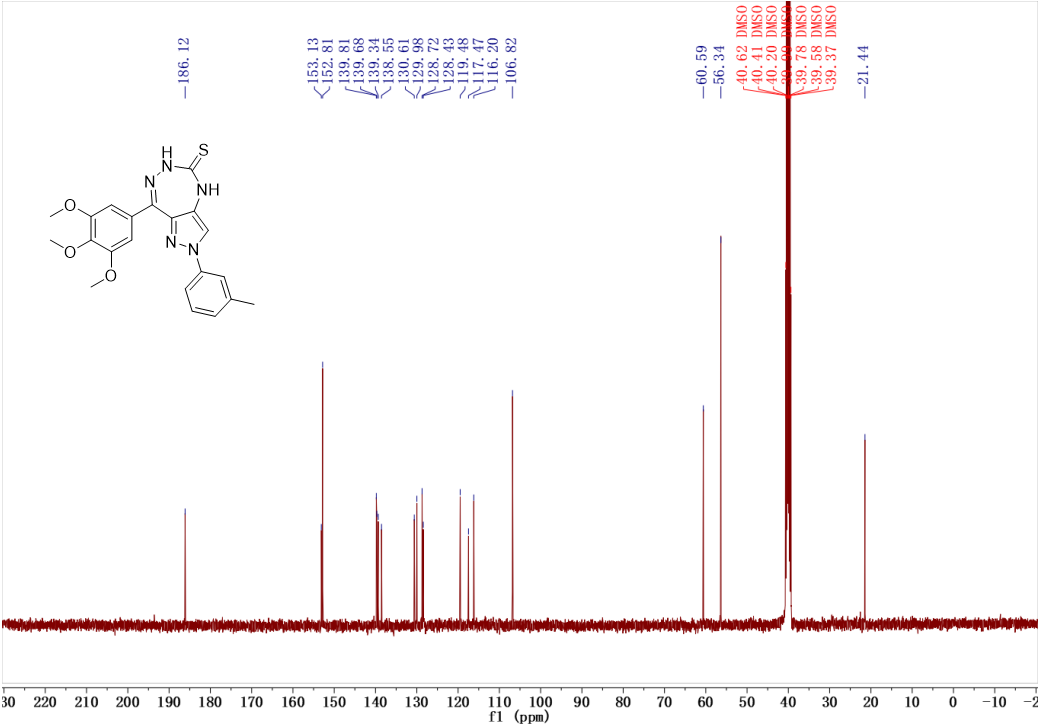


Fig. S42. ^13^C-NMR spectrum of **6b**.


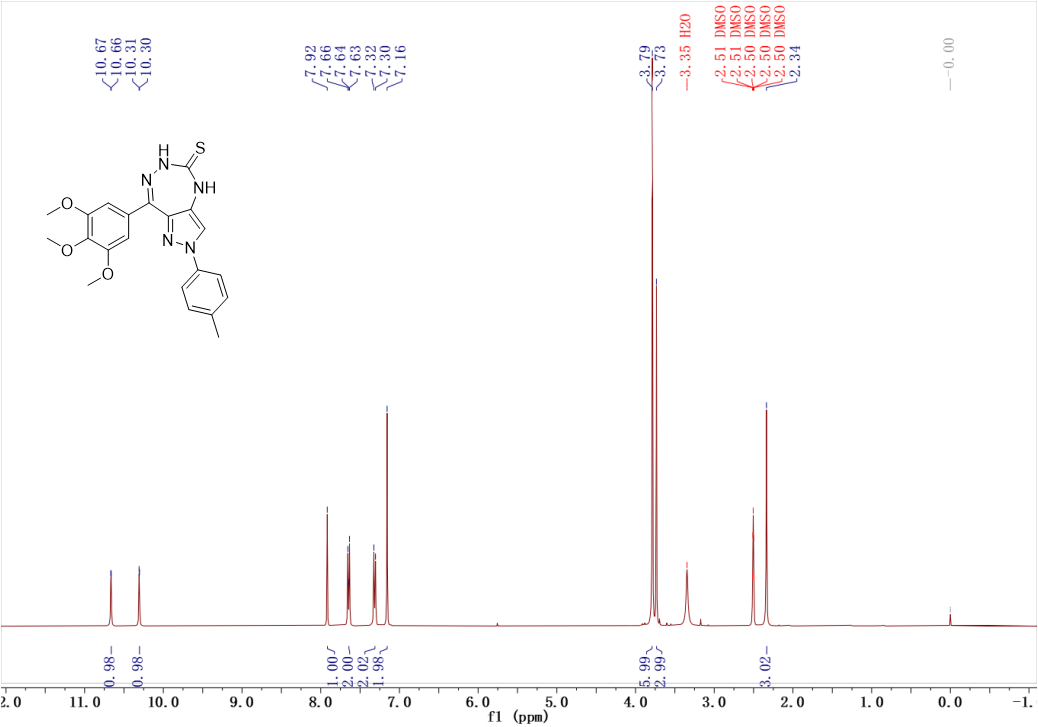


Fig. S43. ^1^H-NMR spectrum of **6c**.


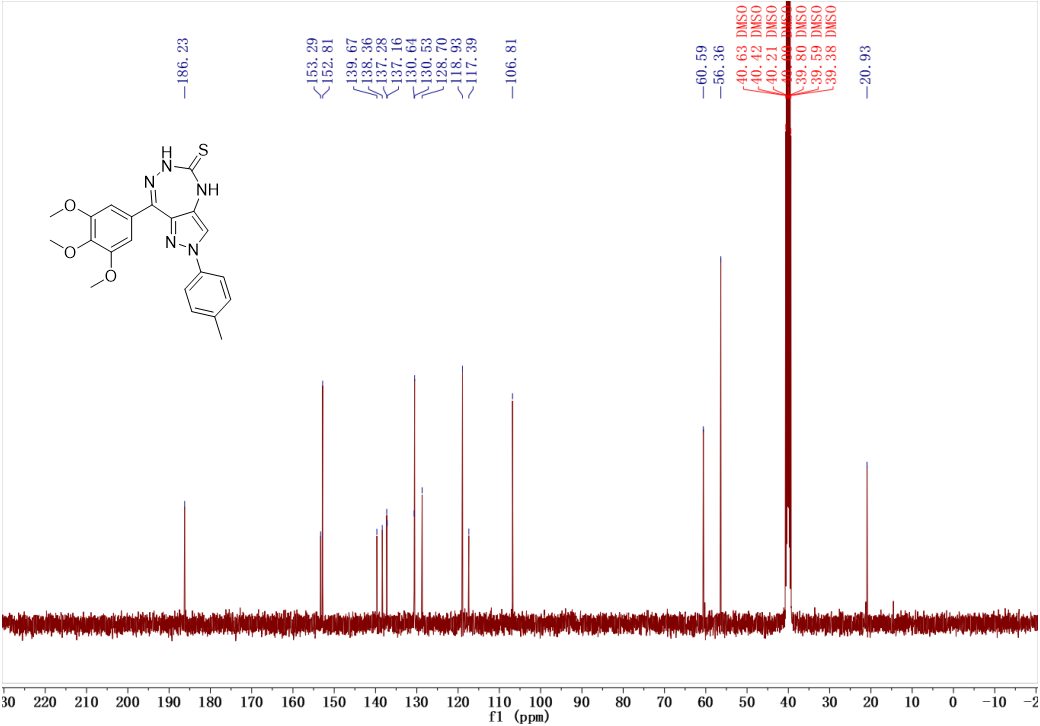


Fig. S44. ^13^C-NMR spectrum of **6c**.


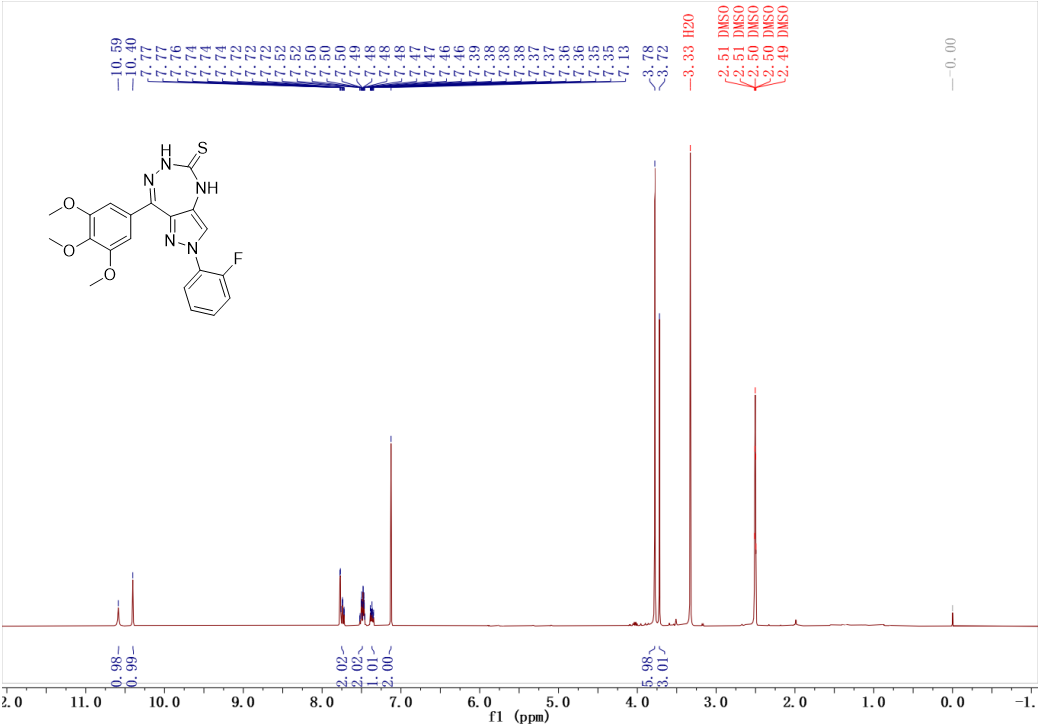


Fig. S45. ^1^H-NMR spectrum of **6d**.


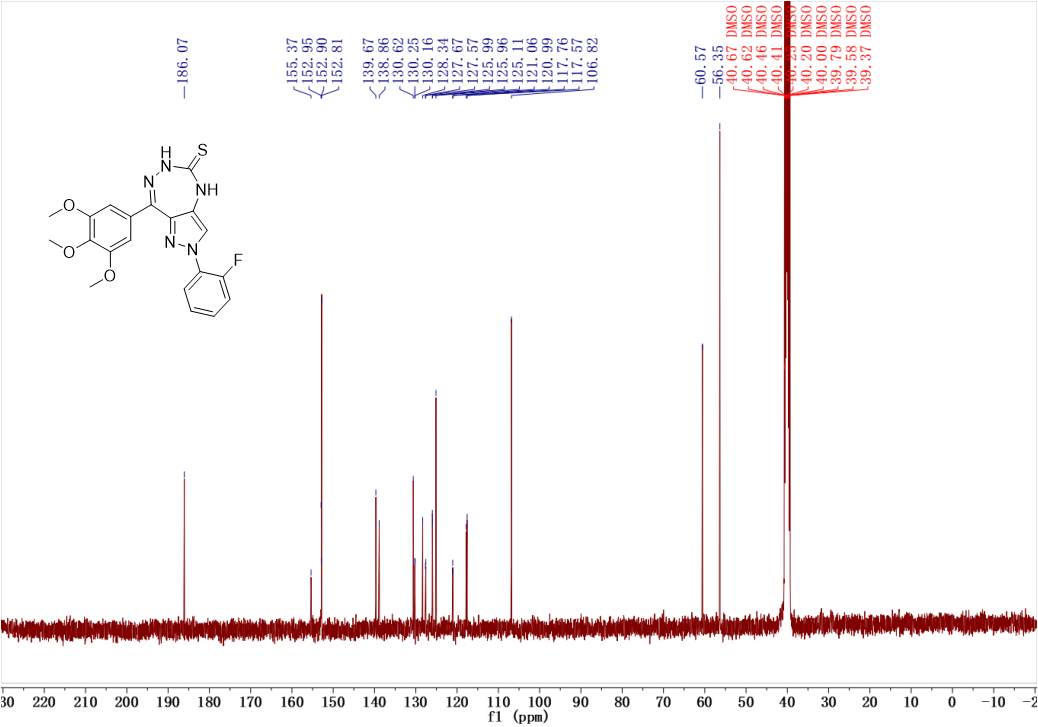


Fig. S46. ^13^C-NMR spectrum of **6d**.


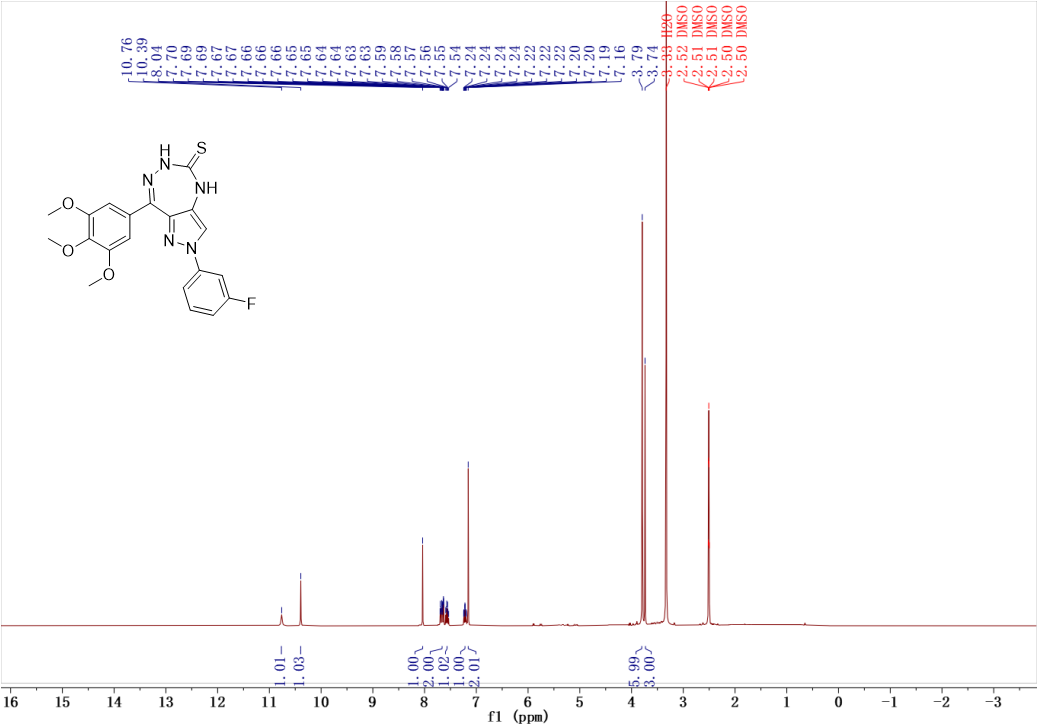


Fig. S47. ^1^H-NMR spectrum of **6e**.


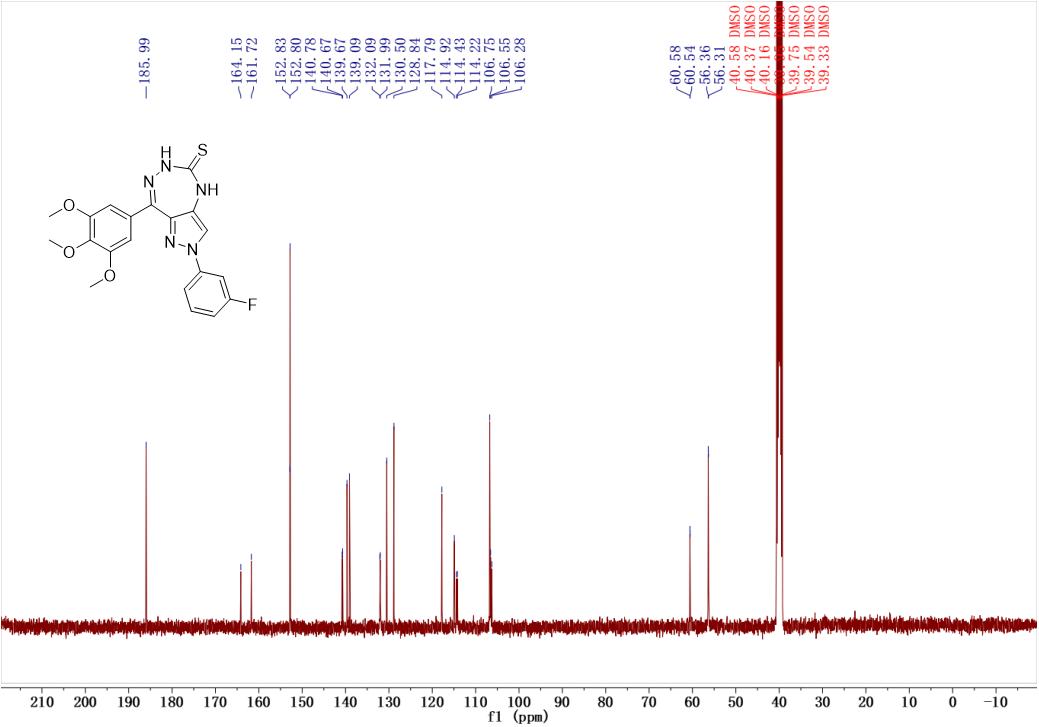


Fig. S48. ^13^C-NMR spectrum of **6e**.


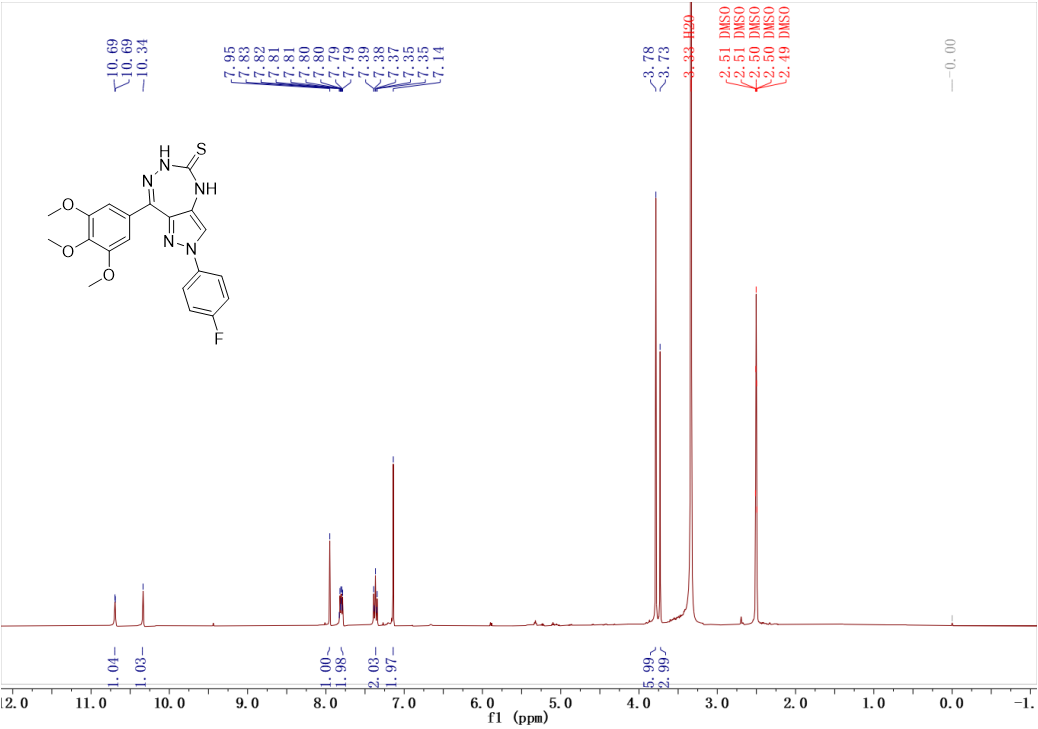


Fig. S49. ^1^H-NMR spectrum of **6f**.


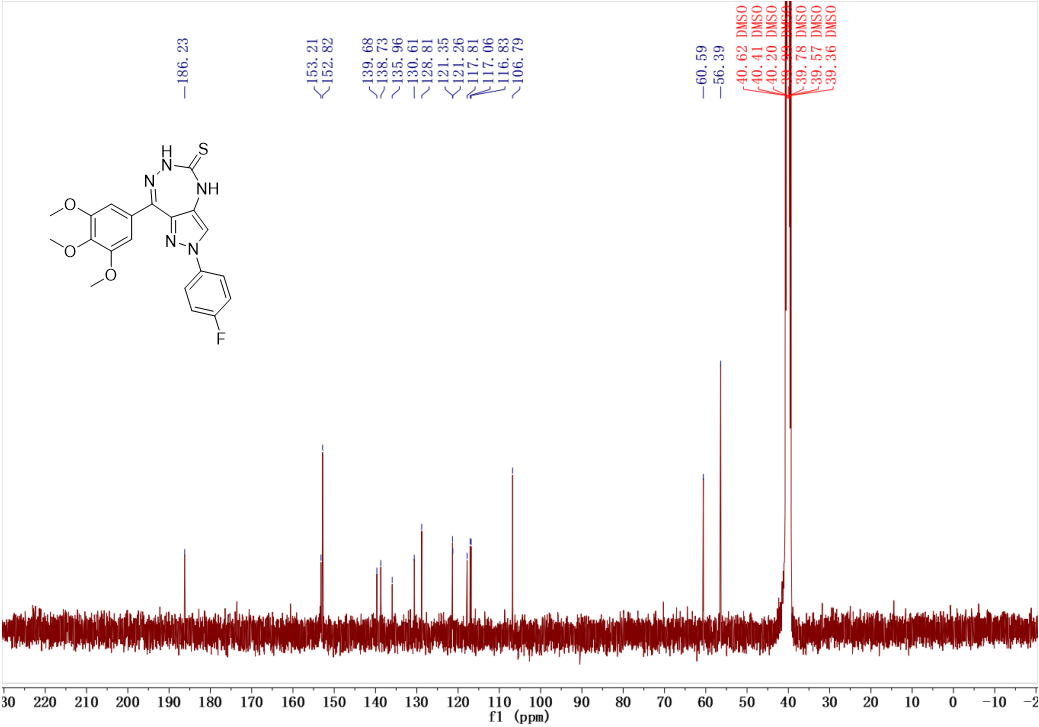


Fig. S50. ^13^C-NMR spectrum of **6f**.


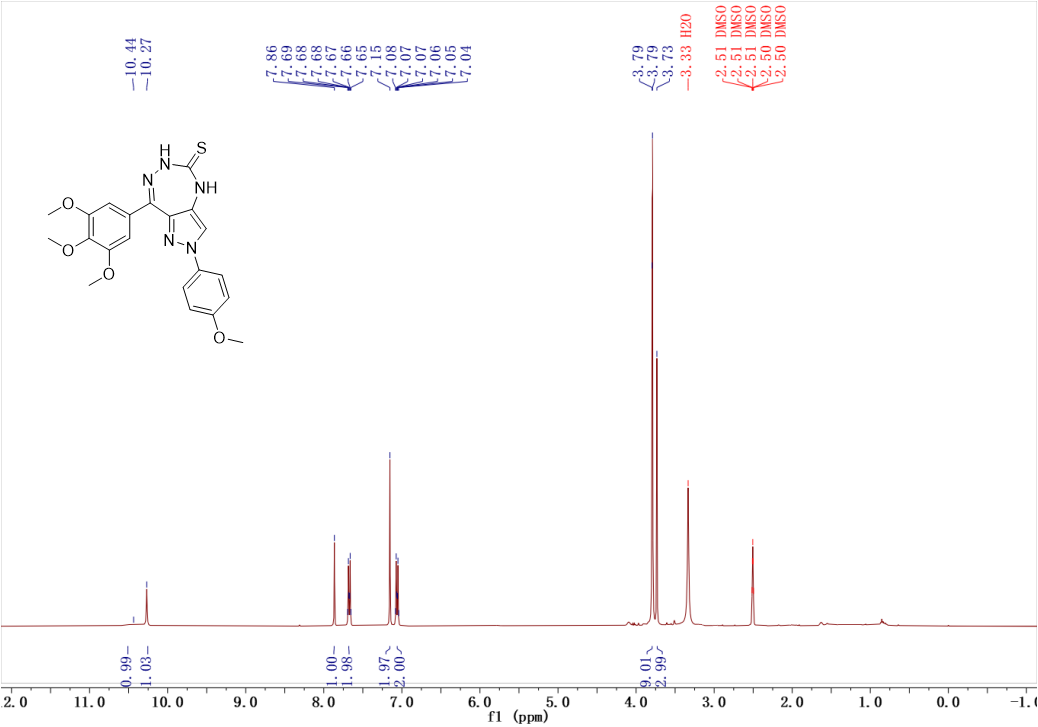


Fig. S51. ^1^H-NMR spectrum of **6g**.


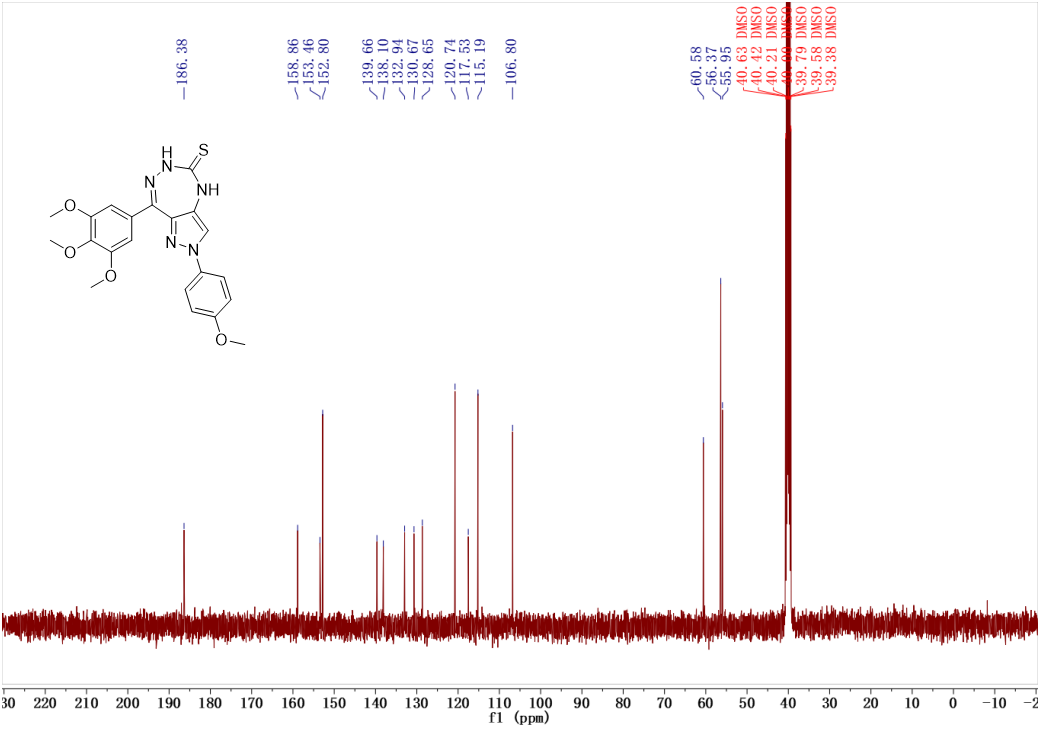


Fig. S52. ^13^C-NMR spectrum of **6g**.


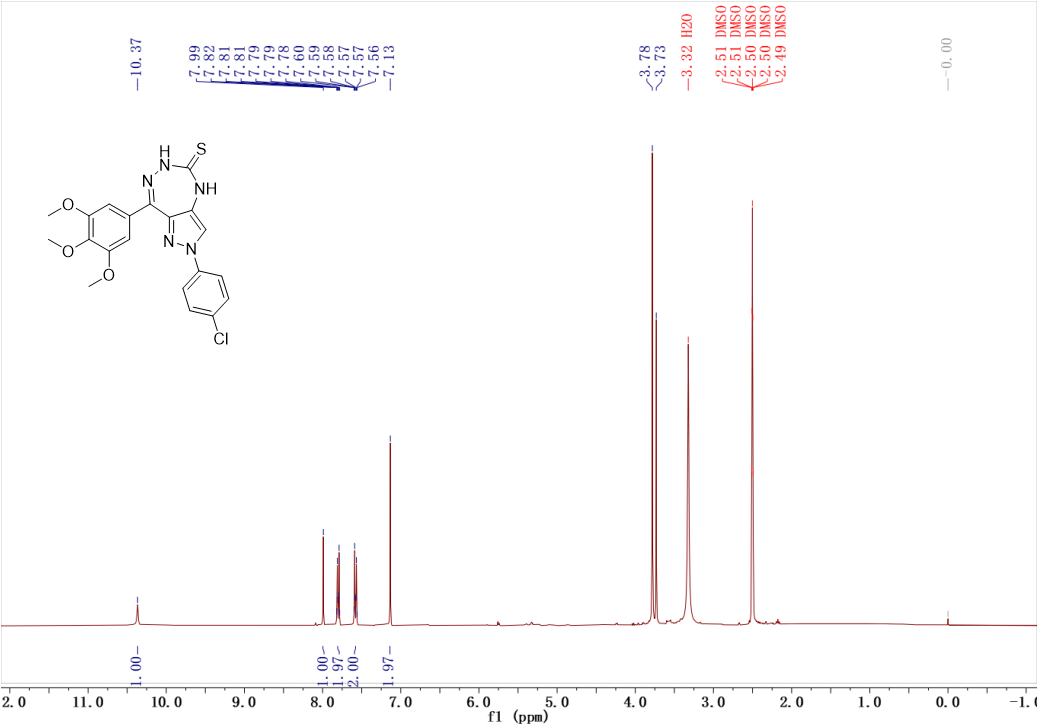


Fig. S53. ^1^H-NMR spectrum of **6h**.


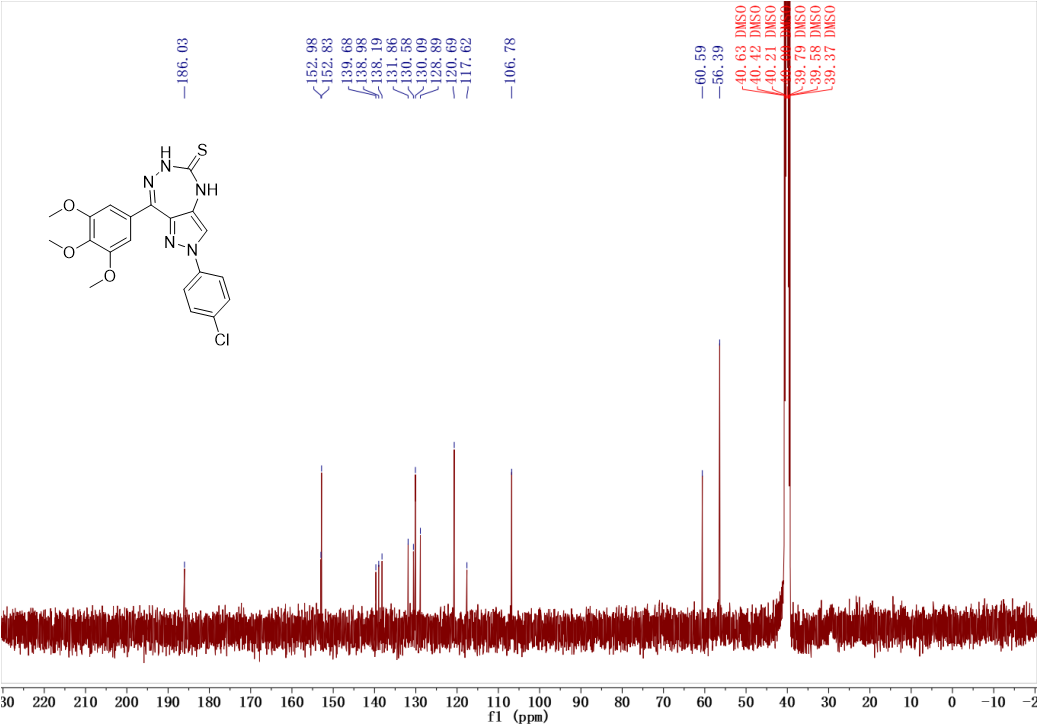


Fig. S54. ^13^C-NMR spectrum of **6h**.


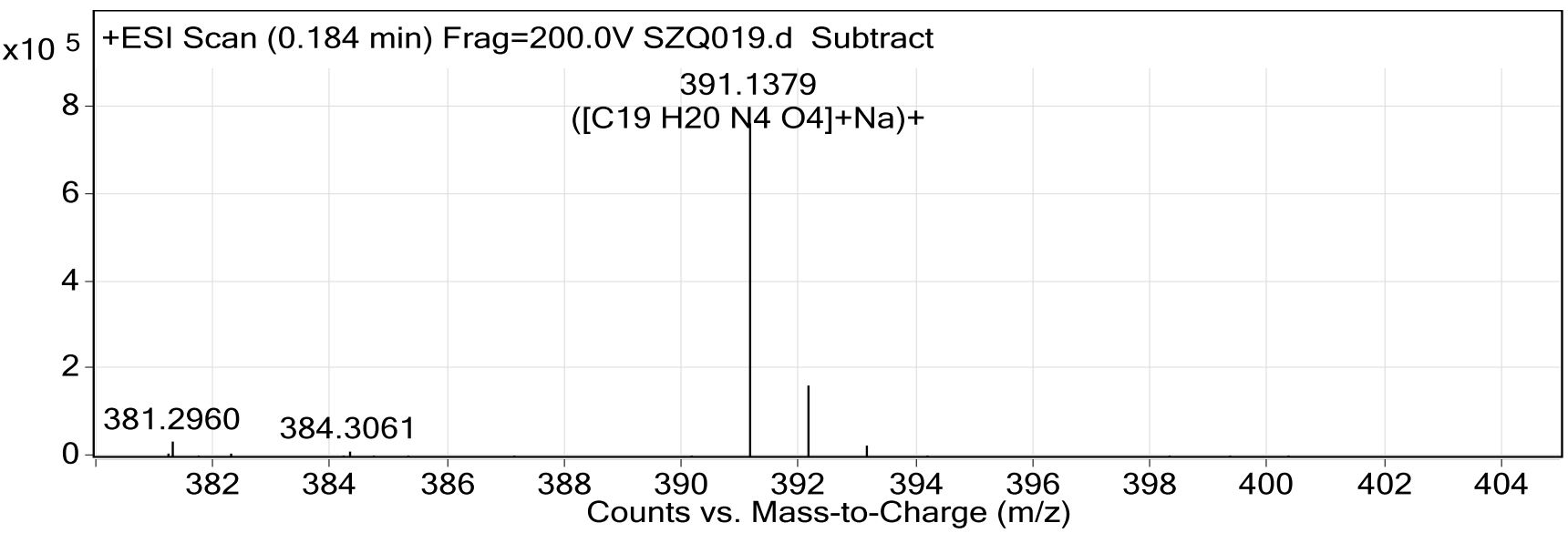


Fig. S55. HRMS spectrum of **4k**.


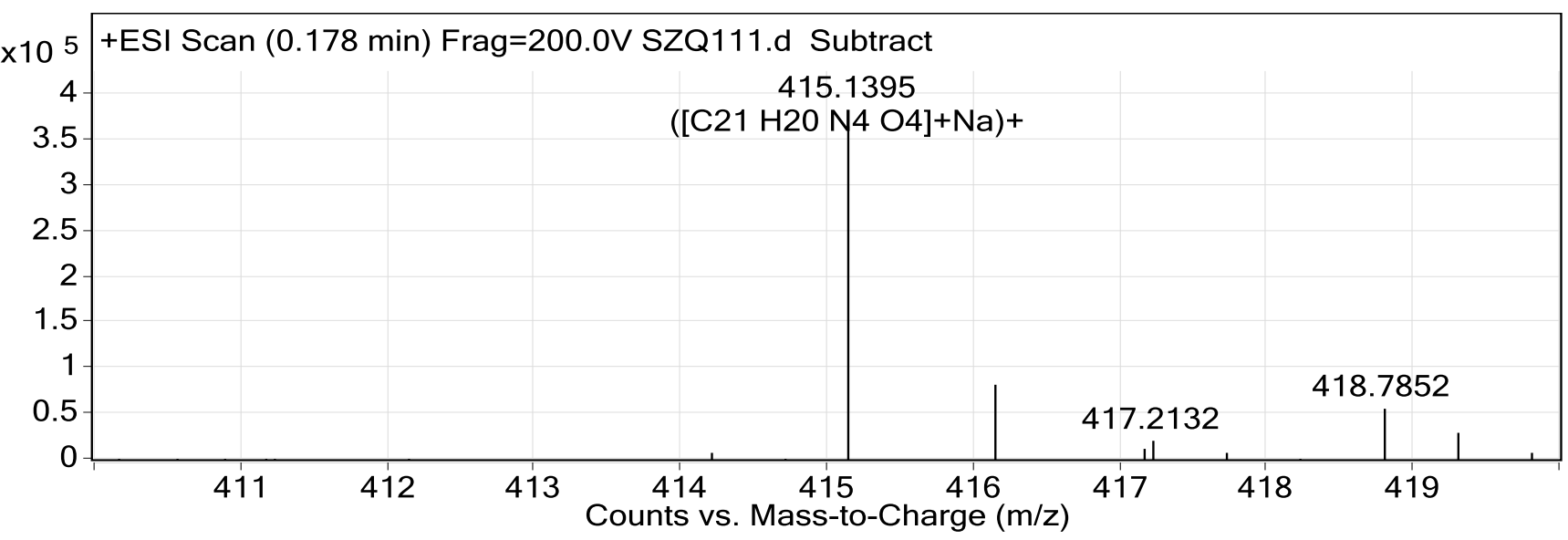


Fig. S56. HRMS spectrum of **5a**.
